# Laccase Directed Lignification Is One of the Major Processes Associated With the Defense Response Against *Pythium ultimum* Infection in Apple Roots

**DOI:** 10.3389/fpls.2021.629776

**Published:** 2021-09-07

**Authors:** Yanmin Zhu, Guanliang Li, Jugpreet Singh, Awais Khan, Gennaro Fazio, Melody Saltzgiver, Rui Xia

**Affiliations:** ^1^Tree Fruit Research Laboratory, USDA-ARS, Wenatchee, WA, United States; ^2^College of Horticulture, South China Agricultural University, Guangzhou, China; ^3^Plant Pathology and Plant-Microbe Biology Section, Cornell University, Geneva, NY, United States; ^4^Plant Genetic Resources Unit, USDA-ARS, Geneva, NY, United States

**Keywords:** apple root, soilborne pathogens, defense activation, plant resistance, small RNA profiling, degradome sequencing, post-transcriptional regulation

## Abstract

Apple replant disease (ARD), incited by a pathogen complex including *Pythium ultimum*, causes stunted growth or death of newly planted trees at replant sites. Development and deployment of resistant or tolerant rootstocks offers a cost-effective, ecologically friendly, and durable approach for ARD management. Maximized exploitation of natural resistance requires integrated efforts to identify key regulatory mechanisms underlying resistance traits in apple. In this study, miRNA profiling and degradome sequencing identified major miRNA pathways and candidate genes using six apple rootstock genotypes with contrasting phenotypes to *P. ultimum* infection. The comprehensive RNA-seq dataset offered an expansive view of post-transcriptional regulation of apple root defense activation in response to infection from *P. ultimum*. Several pairs of miRNA families and their corresponding targets were identified for their roles in defense response in apple roots, including miR397-laccase, miR398-superoxide dismutase, miR10986-polyphenol oxidase, miR482-resistance genes, and miR160-auxin response factor. Of these families, the genotype-specific expression patterns of miR397 indicated its fundamental role in developing defense response patterns to *P. ultimum* infection. Combined with other identified copper proteins, the importance of cellular fortification, such as lignification of root tissues by the action of laccase, may critically contribute to genotype-specific resistance traits. Our findings suggest that quick and enhanced lignification of apple roots may significantly impede pathogen penetration and minimize the disruption of effective defense activation in roots of resistant genotypes. The identified target miRNA species and target genes consist of a valuable resource for subsequent functional analysis of their roles during interaction between apple roots and *P. ultimum*.

## Introduction

Apple replant disease (ARD) is defined by the stunted growth or possible death of newly planted trees in replant sites, where continuous cultivation of apples or closely related species leads to pathogen inoculum accumulation. ARD form a soilborne pathogen complex that includes necrotrophic soilborne oomycetes (*Phytophthora* and *Pythium*) and fungi (*Ilyonectria* and *Rhizoctonia*) (Jaffee et al., 1982; Mazzola, 1998), has been a serious threat for the establishment of economically viable orchards. *Pythium ultimum* is a major component of this pathogen complex in orchard soils worldwide (Mazzola, 1997; Tewoldemedhin et al., 2011). Management of ARD depends almost exclusively on pre-plant fumigation of orchard soils to eradicate ARD pathogens, which also inadvertently eliminates other beneficial soil microbiota. Use of these chemical fumigants is under increasing regulatory restriction due to environmental and human health concerns. Maximized exploitation of host resistance through investigation of the molecular regulation controlling defense responses of apple roots, and thereafter development and deployment of resistant apple rootstocks can offer a cost-effective, environment friendly and durable strategy for ARD management (Zhu et al., 2014; Zhu and Saltzgiver, 2020).

To uncover the molecular mechanisms regulating apple roots resistance responses toward *P. ultimum* infection, two previous transcriptome analyses have provided the first panoramic description of genome-wide transcriptional networks during defense activation (Shin et al., 2016b; Zhu et al., 2017, 2019). The peak transcriptomic changes in apple roots in response to *P. ultimum* infection were shown to be at 48 hpi (hour post inoculation) when about 2% of the protein-encoding genes were differentially regulated (Shin et al., 2016b). A recent comparative transcriptome analysis identified a large number of differentially expressed genes between a *P. ultimum* resistant G.935^®^ and a susceptible B.9 rootstock genotype (Zhu et al., 2019). The candidate genes, which may crucially contribute to resistance traits, include those with annotated function of pathogen perception, hormone signaling, secondary metabolite production, and resistance proteins (Zhu et al., 2017, 2019). Overall, it appears that an earlier and stronger molecular-level defense activation occur in the roots of resistant genotypes as they are challenged by *P. ultimum*. In contrast, deficient detoxification, weakened effector-triggered immunity (ETI), or existence of a susceptibility (*S*) gene may contribute to a disrupted defense activation process in the roots of the susceptible genotype. Parallel to these transcriptome analyses, a systematic phenotyping effort has recently identified a panel of apple rootstock germplasm with contrasting and reliable resistance traits against *P. ultimum* infection (Shin et al., 2016a; Zhu et al., 2018a,b). Besides the expression patterns of the protein coding genes, elucidating post-transcriptional control of genotype-specific defense responses may facilitate the identification of key genes underpinning apple roots resistance traits.

Plant endogenous small RNAs (sRNAs) are a class of short (∼21–25 nucleotides) non-coding single-stranded RNAs, which are ubiquitous in most cellular processes including plant-microbe interactions (Bartel, 2004; He and Hannon, 2004; Eckardt, 2012; Borges and Martienssen, 2015; Weiberg and Jin, 2015). sRNAs can be divided into two major categories based on their distinct biogenesis pathways, i.e., microRNAs (miRNAs) and small interfering RNAs (siRNAs) (Axtell, 2013). sRNAs are generated by DICER or DICER-like (DCL) endoribonucleases, and the mature sRNAs are loaded into Argonaute (AGO) proteins to induce silencing of genes with complementary sequences (Rogers and Chen, 2013; Borges and Martienssen, 2015), which is generally referred to as RNA interference (RNAi). By base-pairing to the target mRNAs of protein coding genes, sRNAs modulate gene expression through either translational repression or post-transcriptional silencing. Accumulating evidence indicates that plant miRNAs play a pivotal role in controlling plant immune responses to pathogen infection through numerous cellular processes (Katiyar-Agarwal and Jin, 2010; Fei et al., 2013, 2016; Bologna and Voinnet, 2014; Chen et al., 2018). For example, the NBS-LRR-miR482/2118 regulatory network has been demonstrated to have a role in fine-tuning the timing and intensity of defense responses in tomato-*Phytophthora infestans* pathosystem (Jiang et al., 2018; Hong et al., 2019). On the other hand, pathogens can hijack the host RNA silencing system to reduce plant defense responses (Weiberg and Jin, 2015; de Vries et al., 2018b, 2019). Profiling miRNAs during interaction between apple roots and necrotrophic oomycete pathogen *P. ultimum*, as well as identifying their corresponding target genes by degradome sequencing (German et al., 2008), should provide critical insights into regulation of resistance in apple roots to this pathogen.

The objective of this study was to identify key miRNA families and their specific target genes in apple roots during interaction with *P. ultimum* using high-throughput RNA sequencing analysis. Six apple rootstock genotypes showing contrasting phenotypes against *P*. *ultimum* infection were included in this study to identify genotype-specific patterns in miRNA expression and the identities of miRNA-targeted genes.

## Materials and Methods

### Apple Rootstock Genotypes and Plant Maintenance

Based on the results from a systematic phenotyping study (Zhu et al., 2018b), six apple rootstock genotypes, i.e., three resistant (or R group: R-#58, R-#161, and R-#164) and three susceptible genotypes (or S group: S-#106, S-#115, and S-#132), were identified and included in this study. These genotypes originated from an “Ottawa 3” × “Robusta 5” (O3R5) apple rootstock F1 population developed in the mid-1970s. Individual plants for each apple rootstock genotype and mock-inoculation controls were produced using synchronized plant tissue culture or micro-propagation procedures, as described previously (Zhu et al., 2018a). Shoot propagation and root induction/elongation required 6 weeks and 4 weeks, respectively. Individual plants with enough root system were transferred to pots containing autoclaved artificial soil consisting of construction sand, vermiculite and perlite in 1:1:1 ratio for a 1-week “in-soil acclimation” before pathogen infection assays and root tissue collection. For tissue culture generated root system, the in-soil acclimation is a critical step to allow further differentiation of root tissues and to fully express the inherent resistance traits. To minimize the effects of transplanting shock on plants, especially in roots from culture medium to soils in pots, a transparent 7′ vented humidity dome (Greenhouse Megastore, Danville, IL, United States) was used to cover the flat tray holding the pots to retain humidity. The temperature in the growth room was approximately 22 ± 1°C at night and 25 ± 1°C during daytime with 12 h light/dark photoperiod regime.

### Inoculation of Apple Roots With *Pythium ultimum* and Root Tissue Collection

The *Pythium ultimum* isolate (#1062) used in this study was originally isolated from the roots of “Gala”/M.26 apple grown at Moxee, WA, United States. Inoculum of *P. ultimum* was prepared as previously described (Shin et al., 2016b). Briefly, the inoculum of *P. ultimum* was prepared by cultivation in potato-carrot broth (20 g of carrots and 20 g of peeled potatoes in 1 L of water boiled for 30 min) with two drops of wheat germ oil added per L of medium. The *P. ultimum* cultures were grown in potato-carrot broth in 9-cm petri dishes at 22°C for 4–6 weeks. Oospores and mycelium from the resultant hyphae mat were collected and ground in 0.5% methyl cellulose solution using a household electric blender for 30 s. The oospores and hyphal fragments were resuspended in 0.5% methyl cellulose to give a final concentration of approximately 2,000 oospores per milliliter (mL). The inoculation of seedlings with *P. ultimum* was performed by dipping the root system in the inoculum solution for 5 s. Inoculated plants were immediately transplanted into pots with autoclaved soils and watered thoroughly. Control plants were mock inoculated with 0.5% methyl cellulose solution and maintained similarly to the *P. ultimum* inoculated plants. All plants were maintained in an environmental growth room as specified above. Root tissues were collected at 48 hpi (hour post inoculation) by excising the roots after excavating plant from pot and quick rinsing off attached soil medium. Three independent biological replicates were set for each sample (genotype/treatment), and each replicate included the pooled root tissues of five plants. Tissues were store in −80°C freezer until RNA isolation.

### Plant Survival Rate and Microscopic Root Necrotic Patterns

A detailed description of the resistance phenotypes for included six genotypes has been reported previously (Zhu et al., 2018b). Briefly, inoculated plants were allowed to grow for 4 weeks in autoclaved potting mix. Plant survival rate for each genotype was recorded at 3, 7, 10, 14, and 28 dpi (days post inoculation), although overall plant survival rate for most of the tested genotypes was stabilized after 7 dpi. Infection assays were repeated at least three times. For microscopic observation, plants were carefully excavated from the soil to minimize mechanical damage to the roots from 2 to 7 dpi. Residual soil along the root branches was gently removed under running tap water. Roots for both mock-inoculated control and *P. ultimum* inoculated plants were kept separately in 100-mL beakers filled with water until microscopic examination within 2 h. Individual root branches were carefully separated from each other, and a glass slide was used to hold the roots in petri dish filled with autoclaved water. A minimum of six plants per genotype from control and *P. ultimum* treated were examined with the assistance of a dissecting microscope (Olympus SXZ12). Images of healthy or necrotic root tissues were obtained using a DP73 digital camera installed on Olympus SXZ12 and the associated software suite of Celsense (Olympus, Center Valley, PA, United States). Digital images were only processed for resizing, cropping and adjusting overall brightness using a publicly available software FastStone Image Viewer 5.5.^1^

### RNA Isolation, Sequencing, Target Genes Identification and phasiRNA Analysis

Total RNA was isolated as previously described (Zhou et al., 2018). Thirty-six libraries were constructed to represent six genotypes, two treatments (mock-inoculation and *P. ultimum* inoculation), and three independent biological replicates for each sample (genotype/treatment). Each biological replicate included the pooled root tissues from five plants. Frozen root tissue samples were ground to a fine powder in liquid nitrogen, and RNA quantity was determined using a NanoDrop spectrophotometer (ND-1000, Thermo Fisher Scientific). The quality of total RNA used for RNA-seq passed the required standard of RNA integrity number (RIN) *x* ≥ 8, which was evaluated using an Agilent 2100 Bioanalyzer. Total RNAs (∼2 μg for each sample) were gel purified and fragments within the 18–30 nucleotide length range were selected to construct a library. Thirty-six sRNA libraries were prepared and sequenced at the Center for Genome Research and Biocomputing in Washington State University using an Illumina HiSeq2500^TM^ (Illumina Inc., San Diego, CA, United States). Clean sequence reads were obtained after removal of the low-quality tags, 3′ adapter null, insert null, 5′ adapter contaminants, poly A reads and reads shorter than 18 nt. The apple miRNAs were annotated as described previously (Xia et al., 2012, 2015). The total number of reads in a given library that perfectly matched the *Malus × domestica* genome was used for the normalization of read abundance. Each miRNA was normalized to 10 million reads. *Malus × domestica* genome sequences (*Malus* × *domestica* HFTH1 Whole Genome v1.0) were downloaded from the GDR.^2^ The microRNA sequence data was deposited in SRA (Sequence Read Archive) at the NCBI website under the accession number SRP295189.^3^ Statistical analysis of measured expression values between genotypes or treatments was performed using Mann–Whitney *U* test.

To identify which specific apple genes are targeted by miRNA-directed degradation after *P. ultimum* infection, three degradome libraries were sequenced and resulted sequence tags were annotated and quantified (German et al., 2008). First library (L1) was constructed using pooled total RNAs from roots of all mock-inoculation controls, second library (L2) from pooled total RNAs from infected root tissues of all three resistant genotypes, and third library (L3) from pooled total RNAs from root tissues of all three susceptible genotypes. Identification of miRNA target genes was conducted by analyzing three degradome libraries with CleaveLand (version 4) pipeline for degradome analysis (Addo-Quaye et al., 2009). Identification of PHAS loci is based on *P*-value calculation, which was developed and modified in previous studies (De Paoli et al., 2009; Xia et al., 2013). PHASMinerCLI from an in-house software “sRNAminer” was used to predict PHAS loci and TargetSoPipe from sRNAminer was used to identify the trigger miRNA of PHAS loci. The degradome *p*-adjusted value < 0.05 is adopted in assigning the identity of target genes.

The expression patterns of three laccase genes were examined using RT-qPCR as previously described (Zhu et al., 2019). The same set of total RNA samples for small RNA-seq were used for RT-qPCR analysis. The total RNA was treated with DNase I (Qiagen, Valencia, CA, United States) and then purified with RNeasy cleanup columns (Qiagen, Valencia, CA, United States). Two micrograms of DNase-treated RNA were used to synthesize first-strand cDNA using SuperScript II reverse transcriptase (Invitrogen, Grand Island, NY, United States) and poly dT (Operon, Huntsville, AL, United States) as the primer. The expression values of target genes were normalized to that of a previously validated internal reference gene (MDP0000095375) specific for gene expression analysis in apple roots 2^–ΔΔCT^ method (the comparative Ct method) (Livak and Schmittgen, 2001). The gene sequences were retrieved from GDR.^4^ Forward and reverse primers were designed using web-based Primer3Plus^5^ and IDT OligoAnalyzer.^6^ Where possible, an optimum annealing temperature of 60°C, a GC content of 40–60%, an amplicon length of 150–180 bp, and a primer length of 20 bp were applied. The primer sequences of three laccase genes and a reference gene were listed in Table 1.

**TABLE 1:** Primer sequences of laccase and reference genes used for qRT-PCR analysis to study their expression patterns in apple roots in response to *Pythium ultimum* infection.

Gene IDs	Gene description	Primers F/R (5′-3′)
MD03G1056400	Laccase-3	F-5′ CAACCCCAGAACAGATCCAG 3′ R-5′ AAACCCAGGAAGAGATGTGC 3′
MD01G1159400	Laccase-5	F-5′ TGGGCAGTCATTCGATTTGT 3′ R-5′ AACAAAGAGGCAGATCCACC 3′
MD16G1147100	Laccase-7	F-5′ TAATCCGCAAGTACGCAACA 3′ R-5′ CAGATCAAGTGGTGGTGGAG 3′
MD02G1221400	Reference gene	F-5′ ATGGAGAGATGGAATGGCAAAG 3′ R-5′ GTGAGCATCGGATCCCATTTAG 3′

### Genomic Location of miR397a and Variant Analysis

A *de novo* genome of “Robusta 5” parent was assembled using long-read PacBio sequences to identify the genomic location of miR397a precursors. The raw sequence quality cleaning and genome assembly was performed as per Canu assembly pipeline parameters with default settings (Koren et al., 2017). The miR397 sequences were aligned to the assembled “Robusta 5” genome to identify corresponding matching sequences using “Blastn” in the National Center of Biotechnology Information (NCBI) Blast software (Johnson et al., 2008). A 20 kb sequence around miR397a genomic locus on matching “Robusta 5” contigs was extracted using a custom script. Coding regions across these sequences were annotated by comparing them with the Golden Delicious double haploid (GDDH13 v1.1) genome (Daccord et al., 2017). In addition, a nucleotide blast search was conducted using online NCBI Blast tool (Johnson et al., 2008) to further annotate the miR397 matching sequences of “Robusta 5.” To identify nucleotide variants across the miR397a matching regions, sequence reads from “Ottawa 3” parent, and six *P. ultimum* susceptible and resistant genotypes were used for SNP (Single Nucleotide Polymorphism) variant calling as described earlier (Singh and Khan, 2019). First, sequence reads from individual genotypes were quality cleaned using the Trimmomatic software (Bolger et al., 2014) with parameters: leading:20, trailing:20, slidingwindow:4:15, avgqual:20, minlen:25. Read sequences below threshold quality of 20 were also removed. Remaining high-quality reads were aligned against the 20 kb miR397 matching sequences of “Robusta 5” as a reference using burrows-wheeler aligner (BWA) with default parameters (Li and Durbin, 2009). The alignment files were used as input to call SNP variants using Genome Analysis Toolkit (GATK version 3.8.0) (McKenna et al., 2010). SNP variant files for each sample were recorded in genotype variant call format (gVCF) with the HaplotypeCaller plugin in GATK. The sample-specific gVCF files were combined to obtain total SNP variants across all samples using the Genotype GVCF plugin in GATK. A final set of SNP variants supported by minimum 10 read sequences were retained using VCFtools software (Danecek et al., 2011).

## Results

### Resistance Phenotypes and Overall Experimental Design

**FIGURE 1:**
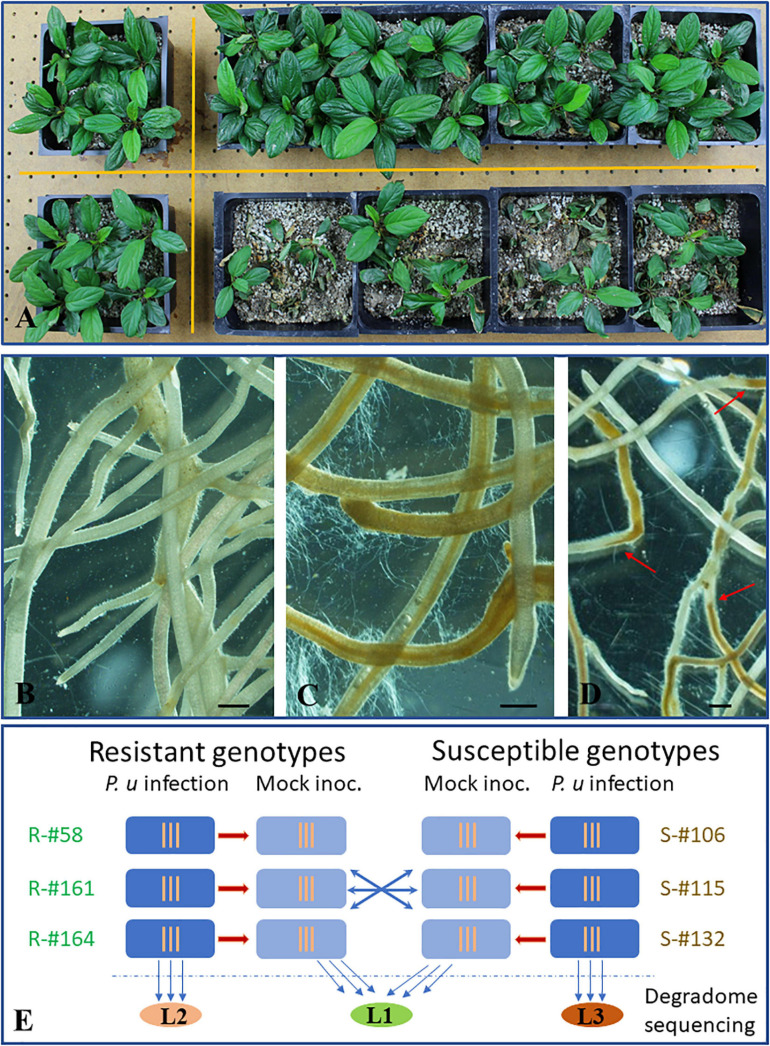
Resistance phenotypes of apple rootstock in response to infection from *Pythium ultimum* and the experimental design for miRNA profiling and target gene identification. **(A)** Plant survival rates between a resistant (top, R-#58) and a susceptible (bottom, S-#106) genotype which were simultaneously inoculated with the same preparation of pathogen inoculum. Plants in the left column were the mock-inoculated control for each genotype. **(B)** Representing image of a mock-inoculated apple root system at 48 hpi; **(C)** Representing image of apple root branches of a susceptible rootstock genotype (S-#132) inoculated with *P. ultimum* at 48 hpi, showing profuse hyphae growth and widespread tissue necrosis and collapses. **(D)** A representing image of apple root branches for a susceptible rootstock genotype (R-#161) inoculated with *P. ultimum* at 48 hpi, showing abrupt necrosis arrest, with distinct lines separating healthy and necrotic sections along infected roots. Bars represent 300 μm. **(E)** Experimental design for microRNA profiling analysis and degradome sequencing. Root tissues at 48 hpi from three resistant (R-#58, -#161, and -#164) and susceptible genotypes (S-#106, -#115, and -#132) were used for small RNA library construction and subsequent high-throughput sequencing. Normalized read counts were compared within and between genotypes. Three vertical lines indicate three biological replicates for each sample (specific genotype/treatment combination). Three colored oval shapes represent three pooled samples for degradome libraries and sequencing analysis, where L1 stands for combined mock-inoculated root tissues (from both resistant and susceptible genotypes), L2 for combined root tissues consisting of infected resistant genotypes, and L3 for combined root tissues consisting of infected susceptible genotypes. Red arrows indicate the defined lines separating healthy and necrotic sections.

Six apple rootstock genotypes included in this study were previously identified to have contrasting resistance traits based on repeated root infection assays with *P. ultimum*, as shown in Figure 1A (Zhu et al., 2018a, b). In addition to remarkable differences of plant survivability, microscopic observation revealed necrosis progression patterns and the intensity of pathogen hyphae growth along infected root that were distinguishable between resistant and susceptible apple rootstock genotypes. Compared to the white and intact tissue of mock-inoculated apple roots (Figure 1B), the sweeping spread of necrotic tissues and profuse growth of pathogen hyphae along the infected roots was frequently associated with the root system of inoculated susceptible genotypes (Figure 1C). In contrast, within the infected root system of the resistant genotypes, the defined lines separating healthy and necrotic sections (Figure 1D, red arrows) were commonly observed, suggesting an effective impediment of pathogen progression. For genotype-specific analysis of miRNA expression profiles and identification of miRNA target genes, three resistant (or R group: R-#58, R-#161, and R-#164) and three susceptible genotypes (or S groups: S-#106, S-#115, and S-#132) were directly compared at the critical stage of defense activation at 48 hpi (hour post inoculation) (Figure 1E).

### Overview of miRNA Sequencing Statistics in Apple Roots in Response to *P. ultimum* Infection

**FIGURE 2:**
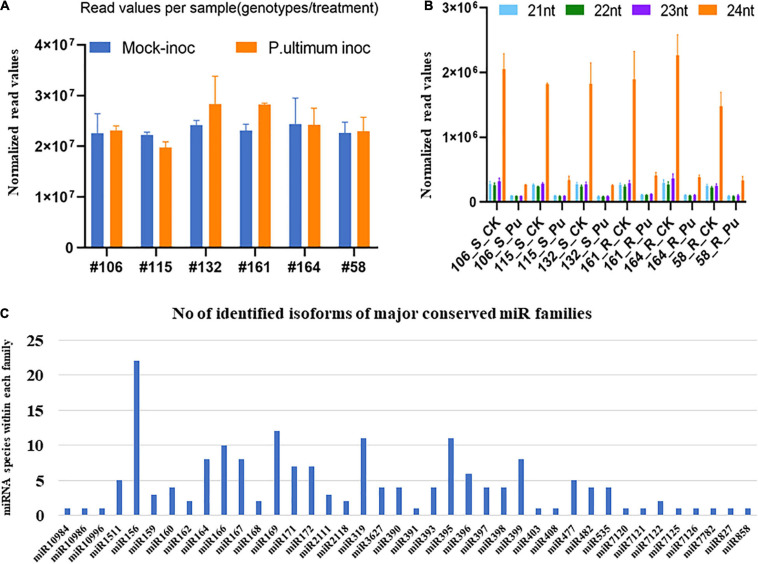
Overall statistics of identified miRNAs in apple roots. **(A)** The average numbers of sequence reads from three replicates for each sample (genotype/treatment). **(B)** Size distribution of small RNAs based on number of unique sequence reads. **(C)** Size of family of major miRNA in apple roots.

A total of ∼2.8 billion reads were generated by Illumina HiSeq2500 from 36 sequenced small RNA libraries. An average of 47% of clean reads among the thirty-six libraries were mapped to the apple genome (Zhang et al., 2019)^7^ according to an established protocol (Xia et al., 2012; Supplementary Table 1). The relative similarity of total read numbers from each sample indicated that neither sampling procedure, library construction, nor sequencing processes created significant bias or errors (Figure 2A). A similar distribution pattern of the size of identified sRNA from thirty-six libraries was observed, with 24 nt (nucleotide) sRNAs being the most abundant, followed by 23, 21, and 22 nt (Figure 2B). A total of 233 (including 50 novel) candidate miRNA species, belonging to 48 known and 39 novel miRNA families were mapped to the apple genome (Zhang et al., 2019; Figure 2C). The complementary miRNA^∗^ sequences for each novel candidate miRNA were also detected, even though most were present at lower copy levels than their corresponding mature miRNAs. The family size ranged from 1 to 22 species among identified miRNA families. Unsurprisingly, the expression levels, as expressed by reads per 10 million, showed vast differences from single digits to 10^6^ of normalized read counts among miRNA species (Supplementary Table 1).

### Expression Patterns of miRNA Families in Response to *P. ultimum* Infection

Among 43 major known miRNA (miR) families, most demonstrated downregulated expression in response to *P. ultimum* infection for all six genotypes, with rare exceptions, while only five miRNA families were upregulated in both resistant and susceptible genotypes, with a few more families specifically in infected root tissue of resistant genotypes (Table 2). Three upregulated miRNA families, miR160, miR167, and miR393 directly participate in auxin signaling, which may represent one of the significant transitions of cellular pathways from normal growth to defense responses in response to *P. ultimum* infection. Seven miRNA families (Table 2) showed differential regulation patterns between resistant and susceptible genotype groups, and five of them were upregulated in resistant genotypes; only the miR3627 family showed the opposite. Majority of the 43 known miRNA families exhibited low to medium levels of expression across the six genotypes, as judged arbitrarily by the normalized read counts at the level of 2–4 digits (Table 2). The remaining six families were highly expressed at the level of 10^5^ to 10^6^ normalized read counts.

**TABLE 2:** Genotype-specificity of miRNA regulation in apple roots in response to *Pythium ultimum* infection.

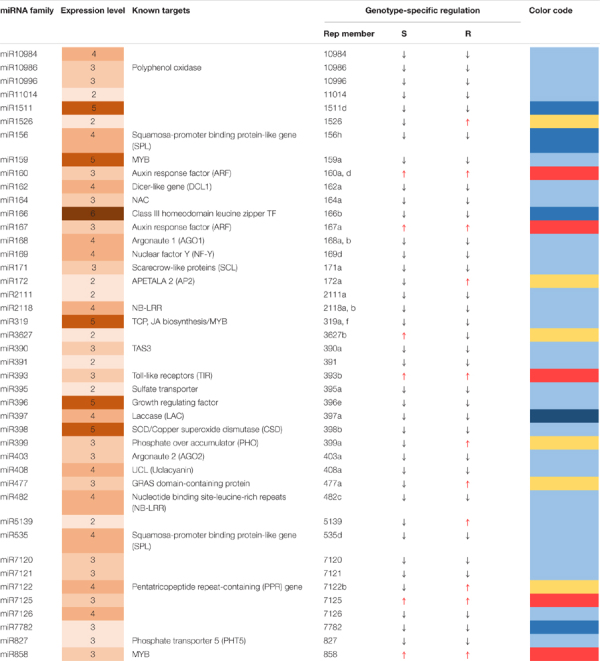

*Column 1 lists known miRNA families identified from current RNA-seq analysis; Column 2 shows the relative expression levels expressed by a heatmap based on the normalized read values per 10 million reads, the numbers indicate the highest exponent of detected maximum count values among the samples (genotype/treatment); Column 3 denotes referred target gene with annotated functions; Column 4 indicates the representing (rep) member from an individual family; Columns 5 and 6 demonstrate the regulation direction due to *P. ultimum* infection, based on the values of corresponding controls and infected tissues, for a specific miRNA family (as exampled by the selected); Column 7 exhibits the visual representation of genotype-specific regulation patterns using “color code”: red for upregulation in *P. ultimum* infected tissues, yellow for differential expression between S and R group (opposite directions); light blue for uniformly downregulation with less than 1x difference comparing to values of control tissue, and darker blue for those with uniformly downregulated expression but with larger than 1x downregulation comparing to values of control tissue. Lower case letters denote various species in the same microRNA families.*

### miRNA Families Regulating Apple Root Immune Responses to *P. ultimum* Infection

**FIGURE 3:**
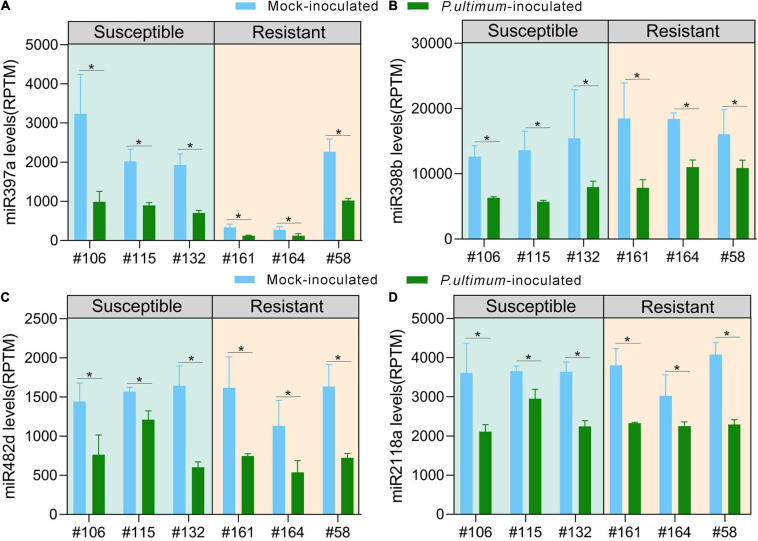
Genotype-specific expression patterns for representing members in selected miRNA families potentially participating in apple roots resistance responses. **(A)** miR397a; **(B)** miR398b; **(C)** miR482d; **(D)** miR2118a. The numbers on *Y* axis represent the average values of the normalized read counts from three biological replicates. Genotype/treatment are aligned along *X* axis; with CK representing mock-inoculation control and “Pu” representing root samples inoculated using *P. ultimum*. Error bars are the standard deviation (SD) of count values within three biological replicates. Error bars represent SE of three independent biological replicates. Asterisks denote statistically significant differences in a one-tailed Mann–Whitney *U* test (*P < 0.05).

Several miRNA families appeared to be directly linked to plant immunity based on their target genes. Although these miRNA families all showed downregulated expression in response to *P. ultimum* infection, subtle variations in the level of downregulation existed between resistant and susceptible groups. Laccase-targeting miR397 showed the consistent and substantial levels of reduced expression. For example, compared to values of mock-inoculation controls, infection from *P. ultimum* resulted in significant reduction of detected read counts for miR397a across all genotypes (Figure 3A). More interestingly, even between the mock-inoculated controls, the resistant genotypes (except R#58) demonstrated strikingly lower levels of miR397a expression compared to those of susceptible genotypes. This observation suggests that a high level of laccase activity (from lower miRNA level) may also function as a part of the pre-formed defense system even before pathogen challenge. The targets of miR398 family include the genes encoding superoxide dismutase (SOD). Together with laccase and other groups, SODs belong to copper proteins, and their roles in early immune responses have been well elucidated (Pilon, 2017; Hong et al., 2019). The expression of miR398 in apple roots were uniformly downregulated upon pathogen inoculation (Figure 3B). However, miR398 had a slightly higher expression level in mock-inoculated tissues of resistant genotypes and a slightly stronger downregulation in infected root tissues of the susceptible genotypes. The regulatory mechanisms of miR398-SOD and their contribution to resistance traits of apple roots deserve further investigation.

Members of the miR482/miR2118 super family are well-documented for their role in targeting specific NBS-LRR encoding genes (Fei et al., 2016; Islam et al., 2018). The entire miR482/miR2118 super family showed uniform downregulation in response to *P. ultimum* infection (Figures 3C,D and Supplementary Table 1), but a closer examination, both miR482a/b as the examples, indicated a subtle variation at the levels of downregulation between resistant and susceptible genotypes. A more substantial (>1x) downregulation was observed for all resistant genotypes, but in comparison, only one out of three susceptible genotypes showed a comparable level in response to pathogen infection. The minor variation, observed at miRNA level, could lead to amplified effect, through phasiRNA generation, on genotype-specific resistance trait toward *P. ultimum* infection.

### miRNA Families Targeting Plant Hormone Signaling and TFs Involved in Defense Activation

Three miRNA families (miR160, miR167, and miR393) that target auxin signaling showed mostly upregulated expression upon *P. ultimum* infection (Table 3). Some members of miR160 family demonstrated differential expression patterns between R and S genotypes. Compared to the consistent upregulation of miR160b in all three susceptible genotypes, its downregulation was observed for two out of three resistant genotypes (R-#58 and R-#164) due to pathogen infection. miR172a, which targets *AP2* and therefore potentially affects ethylene signaling, also demonstrated differential expression between resistant and susceptible genotypes, though its overall expression level was low. While miR172a was downregulated in the roots of all three susceptible genotypes, two out of three resistant genotypes (R-#58 and R-#161) showed upregulated expression. Five miRNA families, i.e., miR396, miR319, miR159, miR858, and miR164, are known to target various TF families including WRKY, TCP, MYB, and NAC that are implicated in JA biosynthesis and secondary metabolism under biotic or abiotic stress conditions (Table 3; Curaba et al., 2014; Liu and Axtell, 2015; Zhang, 2015). However, the overall comparability of their regulation patterns between R and S genotype groups suggested that their post-transcriptional regulations are unlikely the critical factor differentiating resistance and susceptibility, though they are the essential components of defense responses in apple roots. Notably, the targets of these miRNA families, such as those for TFs and hormone signaling, were the commonly identified genes from previous transcriptome analyses (Shin et al., 2016b; Zhu et al., 2019).

**TABLE 3:** Genotype-specific expression patterns for miRNA families regulating hormone signaling and TFs in secondary metabolisms in apple roots in response to *Pythium ultimum* infection.

miRNA family	No of members	Primary target	Representing member	*P. ultimum-inoculation* vs. mock-inoculation					
				S-106	S-115	S-132	R-58	R-161	R-164
160	4	Auxin response factor (ARF)	160b	**1.4**	**1.7**	**1.4**	0.7	**1.2**	0.9
167	8	Auxin response factor (ARF)	167a	**1.8**	**1.7**	**1.4**	**1.5**	**2.5**	**1.8**
393	4	Toll-like receptor (TLR)	393b	**1.1**	**1.1**	0.5	0.9	**1.3**	**1.3**
396	6	Growth regulation factor	396b	0.8	0.8	0.8	0.7	0.8	0.6
319	11	TCP/MYB, JA biosynthesis	319a	0.4	0.7	0.4	0.5	0.5	0.5
172	7	Apetala 2 (AP2)	172a	0.9	0.5	0.3	**1.9**	**1.7**	0.4
159	3	MYB	159a	0.5	0.8	0.4	0.5	0.5	0.4
858	1	MYB	858	**2**	**1.4**	0.8	**1.4**	**1.3**	**1.4**
164	8	NAC	164a	0.5	0.3	0.6	0.3	0.5	0.4

*Expression patterns were expressed by the ratio of normalized read counts of *P. ultimum* infected root tissues over those of corresponding mock-inoculated tissues. The values of larger than 1.0 denote the upregulated expression in response to pathogen infection; Conversely, the values smaller than 1.0 indicate the downregulation due to pathogen infection. The absolute values of the ratio reflect the level of upregulation or downregulation per genotype and miRNA family. Those with upregulated patterns are in bold. The values were calculated using the averages of three biological replicates. Lower case letters denote various species in the same microRNA families.*

### Degradome Sequencing Identified Key Target Genes Due to *P. ultimum* Infection

Degradome sequencing identified a relatively wide, but generally expected, range of categories of target genes specific to this pathosystem between apple roots and *P. ultimum* (Table 4 and Supplementary Table 2). Most of the identified target genes fell into the typical spectrum of reported gene targets for a known miRNA family, although novel targets for several miRNA families were also identified. For example, “betaine aldehyde dehydrogenase” and “cation/calcium exchanger” were identified as the targets of miR156, in addition to its primary known targets of SPLs for this miRNA family. Similarly, “glucan endo-1,3-beta-glucosidase” was among the targets beyond the commonly predicated targets of “homeobox-leucine zipper protein” encoding genes for miR166, and “probable pectate lyase 22” as the target for miR395 (Table 3 and Supplementary Table 2). More practically, this experimental approach allowed the pinpointing of a small number of specific genes from a large gene family, which are specific to this pathosystem. For example, only five NBS-LRR encoding genes out of potentially a few hundred in the apple genome were identified as targets for miR482 from this pathosystem. On the other hand, only two ARF genes were shown to be the targets for miR160 out of several dozens of likely ARF family members. Great variation of the detected “tag abundance” existed per gene/miRNA species/library, from a single digit to over a thousand (HF12099, polyphenol oxidase), although this value can be dependent on the expression levels of both the target gene and miRNA. For some genes, such as those encoding laccases, it appeared the cleavage activity was reduced in *P. ultimum* infected tissues for both resistant and susceptible genotypes (L2 and L3, respectively), as compared with the mock-inoculation control tissue (L1). For other genes such as those encoding MYBs targeted by miR858, an elevated cleavage activity was observed for most target genes. In many cases, more than one member of the same miRNA family were shown to target the same gene ID, such as those genes encoding “growth regulating factors” that were targeted by miR396 species and genes encoding “auxin response factor” targeted by miR167 species. In contrast, many other genes showed a strict one (miRNA species) to one (gene ID) interaction pattern, such as those genes encoding laccase and targeted by miR397 species and R genes targeted by miR482 species. These identified target genes consist of valuable candidates for subsequent functional analysis to define their specific contribution to apple roots resistance to *P. ultimum*.

**TABLE 4:** Identified target genes, their functional annotations and detected tag abundance in apple roots in response to *Pythium ultimum* infection.

Member(s) of miRNA	Target gene	Functional annotation of identified target genes	Tag abundance		
			L1	L2	L3
miR10984	HF07668	ASC1-like protein	N/A	5	N/A
miR10986	HF12097	Polyphenol oxidase, chloroplastic	92	N/A	N/A
	HF12099		1417	1426	724
	HF12100		1390	1493	739
	HF12105		146	N/A	43
	HF17936		105	139	154
	HF17941		3	N/A	N/A
miR156a, c, d, e,	HF26180	Squamosa promoter-binding-like protein 12	47	44	21
f, I, k, l, n, o, u, v	HF35297		56	36	N/A
	HF42804		N/A	46	N/A
	HF41242	Betaine aldehyde dehydrogenase 1, chloroplastic	N/A	N/A	12
	HF42782	U-box domain-containing protein 6	N/A	2	2
	HF42611	Optineurin	N/A	N/A	3
	HF05261	Probable polyribonucleotide nucleotidyltransferase 1	10	N/A	N/A
	HF30813	Cation/calcium exchanger 4	N/A	14	N/A
	HF26192	Teosinte glume architecture 1	75	77	27
miR159a-c	HF17403	Transcription factor GAMYB	6	N/A	N/A
	HF06666*		119	N/A	N/A
	HF34775	Patellin-3	N/A	13	N/A
	HF03195	Protein SPEAR1	32	N/A	11
	HF03914*		42	20	N/A
	HF12011	Receptor-like protein kinase HSL1	6	N/A	N/A
miR160a, d	HF06172*	Auxin response factor 18	47	N/A	6
	HF40525*		48	43	N/A
	HF01570		65	64	27
	HF04836		N/A	59	N/A
	HF24306*		N/A	47	15
	HF25001*		69	N/A	N/A
	HF04836		64	N/A	34
	HF44485*	Auxin response factor 17	N/A	818	N/A
	HF07133*		N/A	869	426
	HF00444	Solute carrier family 25 member 44	12	N/A	N/A
miR162a, b	HF05621*	Endoribonuclease Dicer homolog 1	N/A	66	27
	HF20945	ATP-dependent zinc metalloprotease FTSH	N/A	7	N/A
miR164a, d, h	HF09293*	NAC domain-containing protein 100	653	N/A	112
	HF24823*		642	488	N/A
	HF27440	Methionine aminopeptidase 1D	N/A	N/A	3
	HF16100	U-box domain-containing protein 35	5	N/A	N/A
	HF11267	NAC domain-containing protein 21/22	588	438	116
	HF22809		559	419	118
miR166a, j	HF00268*	Homeobox-leucine zipper protein ATHB-15	174	N/A	100
	HF28547*		N/A	196	100
	HF12939*	Homeobox-leucine zipper protein ATHB-8	N/A	174	N/A
	HF21765*		174	195	N/A
	HF40749	Glucan endo-1,3-beta-glucosidase 14	19	N/A	N/A
	HF06176*	Homeobox-leucine zipper protein REVOLUTA	N/A	170	111
	HF40517*		446	N/A	N/A
	HF11732*	Homeobox-leucine zipper protein HOX32	N/A	118	N/A
	HF23204		114	113	54
miR167a, f, j, h	HF32107*	Auxin response factor 8	620	N/A	402
	HF37005*		N/A	651	375
	HF11771*	Auxin response factor 6	95	86	N/A
	HF34014		95	N/A	48
	HF18290*		N/A	77	57
	HF29114		78	74	41
miR168a, b, d	HF09608*	Protein argonaute 1	N/A	299	166
	HF13064	Mannose-P-dolichol utilization defect 1 protein homolog 2	N/A	N/A	3
	HF36007	Thaumatin-like protein 1	5	N/A	N/A
miR169d	HF19226	Pentatricopeptide repeat-containing protein At2g17670	2	N/A	N/A
miR171a, b, c, e, g	HF27082*	Scarecrow-like protein 6	990	N/A	474
	HF37501*		N/A	21	N/A
	HF01470*		N/A	20	7
	HF00464	Phosphopentomutase	4	N/A	N/A
	HF07177	Nodulation-signaling pathway 2 protein	12	25	8
	HF44555		11	10	N/A
	HF44340	Sugar transport protein 5	N/A	N/A	8
	HF10118	Alkylated DNA repair protein alkB homolog 8	4	N/A	N/A
	HF24442	Interleukin-3 receptor class 2 subunit beta	N/A	4	N/A
miR172a, d, f, g	HF40428*	Ethylene-responsive transcription factor RAP2-7	453	N/A	193
	HF06289*		N/A	481	205
	HF24247*		N/A	88	N/A
	HF25060*		N/A	57	22
	HF01637	Floral homeotic protein APETALA 2	18	N/A	7
	HF27401*		7	N/A	7
	HF04766*		N/A	4	N/A
	HF31388		13	11	N/A
miR2111a, c	HF32972*	F-box/kelch-repeat protein At3g27150	N/A	58	35
miR2118a, b	HF17618	Protein MARD1	7	N/A	N/A
	HF44440		N/A	N/A	6
	HF07401	Protein JINGUBANG	3	N/A	N/A
miR319a, c, f	HF03499*	Transcription factor MYB101	N/A	8	4
	HF16566	Transcription factor GAMYB	118	119	64
miR395a, b, e, h, k	HF01798*	ATP sulfurylase 1, chloroplastic	223	293	N/A
	HF13989*		105	N/A	96
	HF07090	Low affinity sulfate transporter 3	3	N/A	N/A
	HF09045*	Probable pectate lyase 22	N/A	3	N/A
	HF41418		N/A	N/A	5
	HF35421*	Sulfate transporter 2.1	12	17	N/A
	HF34432	Mitochondrial uncoupling protein 1	N/A	N/A	2
miR396a-f	HF00473	Growth-regulating factor 5	2	N/A	N/A
	HF30789		73	70	9
	HF19000*		N/A	23	9
	HF07761	Caffeoylshikimate esterase	21	N/A	N/A
	HF21477	Growth-regulating factor 6	170	142	82
	HF13165		201	215	129
	HF27291	Growth-regulating factor 1	113	88	35
	HF31510*		N/A	95	N/A
	HF42039	Dynein light chain, cytoplasmic	3	3	2
	HF00333	LRR receptor-like serine/threonine-protein kinase	N/A	2	N/A
	HF13870	Probable ATP-dependent DNA helicase CHR12	N/A	N/A	6
	HF08723	Growth-regulating factor 8	24	17	5
	HF41682*		5	N/A	N/A
	HF26054*	Growth-regulating factor 7	2	N/A	N/A
	HF27171*	Growth-regulating factor 12	20	15	N/A
	HF36486*	Growth-regulating factor 4	244	N/A	120
	HF19974*		N/A	21	10
	HF02750*	60S ribosomal protein L18-2	42	22	N/A
	HF10798	E3 ubiquitin-protein ligase CHIP	14	N/A	N/A
	HF41728	Transcription factor LHW	4	N/A	2
miR397b	HF23917	Laccase-5	2	11	N/A
	HF26400	Laccase-7	30	N/A	N/A
	HF27792		45	N/A	82
	HF40034	Laccase-3	15	N/A	N/A
miR398a-d	HF42086	Multicopper oxidase LPR2	24	20	7
	HF30403	Umecyanin	N/A	1442	1135
	HF25617*	Copper transporter 6	110	58	N/A
	HF06452		92	71	19
	HF01373	Copper chaperone for superoxide dismutase	283	298	129
	HF08261		290	293	181
	HF29451	Dehydration-responsive element-binding protein 2A	12	7	N/A
	HF40618	Superoxide dismutase [Cu-Zn], chloroplastic	17	15	N/A
	HF41261	LIM domain-containing protein WLIM1	64	N/A	46
	HF31034	Serine/threonine-protein phosphatase PP1 isozyme 4	N/A	N/A	4
miR399c, e, g	HF05992	Heme-binding protein 2	N/A	11	N/A
	HF38390	GRF1-interacting factor 1	N/A	4	N/A
miR408	HF17186	Basic blue protein	2	N/A	3
	HF20292	Nudix hydrolase 23, chloroplastic	3	N/A	N/A
miR477a, b	HF05240	DTW domain-containing protein 2	7	7	6
	HF41450	DELLA protein GAI1	3	3	N/A
miR482a, c, d	HF37161	Disease resistance protein RPM1	N/A	N/A	112
	HF43810	Disease resistance protein At4g27190	9	N/A	5
	HF01646	Putative disease resistance protein RGA1	4	N/A	6
	HF04762	Putative disease resistance protein RGA3	11	N/A	N/A
	HF40153	Putative disease resistance protein At1g50180	4	N/A	7
	HF07514	Probable apyrase 6	16	N/A	N/A
	HF40855	Pro-apoptotic serine protease nma111	N/A	6	N/A
	HF00026	Chaperone protein dnaJ 8, chloroplastic	194	200	276
	HF32497		191	197	251
miR5225a	HF20446	Putative disease resistance protein RGA4	N/A	N/A	2
miR535b, d	HF02059	Ribosome maturation factor RimM	2	N/A	N/A
	HF32583	Pyrophosphate-energized vacuolar membrane proton pump	17	N/A	N/A
miR7122a, b	HF44234	Pentatricopeptide repeat-containing protein At1g12700	N/A	187	162
miR7125	HF11513	Zinc transporter 1	189	133	121
miR7126	HF16036	E3 ubiquitin protein ligase DRIP2	N/A	N/A	4
miR7782	HF21767	Thioredoxin-related transmembrane protein 2	8	9	8
miR858	HF00466	Transcription repressor MYB4	56	166	103
	HF28765		35	108	57
	HF08482	Transcription factor MYB26	3	N/A	N/A
	HF13276	Anthocyanin regulatory C1 protein	62	182	117
	HF18993		41	87	93
	HF21423		63	150	77
	HF13279	Transcription factor MYB3	49	72	81
	HF16086	Transcription factor MYB15	18	60	68
	HF21717	Transcription factor MYB44	15	36	31
	HF24028	Transcription factor MYB1	4	4	10
	HF29485	Transcription factor MYB102	2	N/A	N/A
	HF30785	Transcription factor TT2	44	88	76
	HF05712	Transcription factor MYB7	N/A	40	47

L1 represent for the degradome libraries constructed from pooled root tissues of mock-inoculation including both resistant and susceptible genotypes, L2 for the degradome libraries constructed from pooled root tissues of *P. ultimum*-inoculation resistant genotypes, and L3 for the degradome libraries constructed from pooled root tissues of *P. ultimum*-inoculation susceptible genotypes. *After individual gene ID indicates the individual gene ID was targeted by another miRNA species from the same family. Refer to Supplementary Table 2 detailed information, such as the *P* value and degradome value for each gene ID, and the cleavage site sequence. Lower case letters denote various species in the same microRNA families.

**FIGURE 4:**
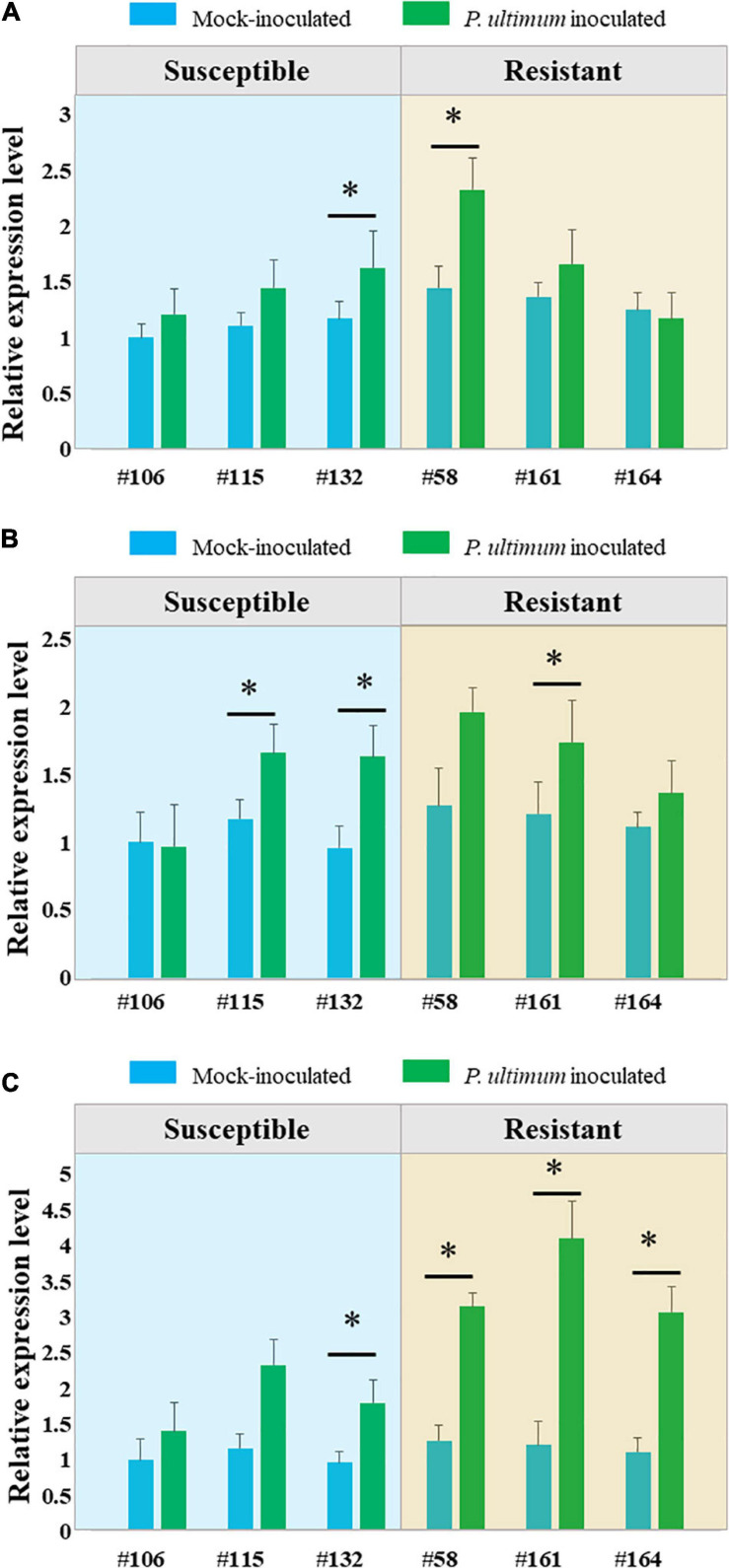
Genotype-specific expression patterns of three laccase encoding genes in apple roots in response to *Pythium ultimum* infection. **(A)** Laccase-3; **(B)** Laccase-5 and **(C)** Laccase-7. The expression levels of these genes were measured by qRT-PCR. Genotypes are listed along *X* axis, with susceptible genotypes grouped at the left panel, and resistant genotypes grouped at right panel. Blue bar represents value of mock-inoculated root tissue and green bar represents the value of *P. ultimum* inoculated root tissue for each genotype. The values on *Y* axis represent the fold change of detected transcript level after normalizing to a reference gene (MD02G1221400) and calibrating by values of mock inoculated root tissue of the susceptible genotype #106. The values are the average of three biological replicates, and error bars are the standard deviation (SD) of fold changes. Error bars represent SE of three independent biological replicates. Asterisks denote statistically significant differences in a one-tailed Mann–Whitney *U* test (^∗^*P* < 0.05).

Three laccase encoding genes (laccase −3, −5, and −7), the target genes of miR397b based on degradome sequencing analysis, showed mostly upregulated expression patterns in response to *P. ultimum* infection (Figure 4). Among them, laccase-7 appeared to be more responsive to *P. ultimum* infection especially for resistance genotypes. For example, the detected transcript level of laccase-7 was increased three-fold in the infected root tissues of the resistant genotype #161, as compared to the mock-inoculated control tissue. The upregulated expression of these target genes corresponded to the downregulated expression of miR397b in response to *P. ultimum* infection. The observation of upregulated expression of these apple laccase genes in *P. ultimum* infected apple root tissues is consistent with the findings from a previous comparative transcriptome analysis between a resistant genotypes G.935 and a susceptible genotype B.9, which showed a peak response at 48 hpi and with stronger induction in the roots of the resistant genotype (Zhu et al., 2019). Therefore, the expression profiles of these laccase encoding genes are consistent with the findings from degradome sequencing, i.e., the reduced expression of miR397b and attenuated cleavage events likely contributed to the upregulated expression of these laccase genes in the infected apple root tissues.

### Genotype-Specific Expression of Selected miRNAs and Their Target Cleavage Activity

MiR397b-known-5p-mature is one of the four members from this family abundantly expressed in apple roots, and its cleavage activity on target (laccase) genes was only detected for miR397b (Tables 4, 5). One of the notable features regarding the genotype-specific miR397b expression, similar to miR397a (Figure 2), was that it expressed at a significantly lower level in mock-inoculated root tissues of resistant genotypes (except R-#58) compared to that of the susceptible genotypes. In response to *P. ultimum* infection, its expression was downregulated for all genotypes. Nevertheless, the lowest average value of read counts was detected in the infected root tissues of the resistant genotypes. The data from degradome sequencing indicated that three laccase encoding genes that are homologous to *Arabidopsis thaliana* laccase −3, −5, and −7, were the targets of miR397b. Furthermore, a substantially higher cleavage activity was observed in the infected root tissues of susceptible genotypes (L3), compared to that in resistant genotypes (L2). Taken together, the reduction of miR397b may result in less cleavage of laccase target mRNA, which could lead to higher laccase enzyme activity, possibly contributing to the observed resistance to *P. ultimum* infection.

**TABLE 5:** The genotype-specific expression of miR397b and target genes in response to *Pythium ultimum* infection of apple roots.

Genotype-specific expression: miR397b-known-5p-mature						
**Susceptible genotypes**						

	**Control**			***P. ultimum* infected**		
	
#106	962.179	1088.28	1174.93	511.3	491.7	548.6
#115	864.0	945.8	1100.5	604.4	593.9	608.5
#132	977.9	1491.3	1115.1	300.2	416.5	398.1
	**Ave (S_CK)** = **^a^1080.0**			**Ave (S_Pu)** = **^b^497.0**		

**Resistant genotypes**						

	**Control**			***P. ultimum* infected**		
	
#58	802.8	1392.7	777.4	600.5	448.1	541.7
#161	583.8	528.8	590.2	248.8	244.5	261.3
#164	475.9	341.7	534.1	335.7	339.5	306.6
	**Ave (R_CK)** = ^a^669.7**			**Ave (R_Pu)** = ^b^369.6*		

**The identified target genes based on degradome sequencing:** Laccase-5 OS = *Arabidopsis thaliana* GN = LAC5 PE = 2 SV = 1 (also, laccase-3 and laccase-7) (*P*-value = 0)						

**Cleavage site**	**Target site sequence**			**Tag abundance**		
				L1	L2	L3
713	AGUCAUCAACGCUGCACUCAA			2	11	N/A
662	CCUAAUCAACGCUGCACUCAA			30	N/A	N/A
647	CCUAAUCAACGCUGCACUCAA			45	N/A	82
779	AGUCGUCAACUCUGCACUCAA			15	N/A	N/A

The values in the top section of the table, are the normalized read counts for each replicate per genotype/treatment. The average values are calculated for individual group (R vs. S), and under either mock-inoculation or *P. ultimum* infection. Different letters (a or b) in front of the average values indicate the significant difference between treatments (mock-inoculation and *P. ultimum* inoculation). Asterisks denote statistically significant differences in a one-tailed Mann–Whitney *U* test (*P < 0.05) between genotype-groups (resistant and susceptible) of the same treatment (mock-inoculation or *P. ultimum* inoculation).

Resistance protein encoding genes, or R genes, are evidently the key players during plant-pathogen interactions. The differential expression patterns of miR482 between R and S genotypes and specifically identified targeted R genes by degradome sequencing were among the key findings from the current study (Table 1 and Supplementary Table 1). For example, miR482c-known-3p-mature is one of the three members in miR482 family which showed intriguing expression patterns among six genotypes and in response to *P. ultimum* infection (Table 6). In response to *P. ultimum* inoculation, the levels of miR482c were downregulated in both R and S genotypes, but with slightly larger reduction (>1x) in R group than that of S groups (<1x). The degradome sequencing identified the target of miR482c-known-3p-mature is Arabidopsis RPM1 homolog encoding gene, with two cleavage sites. Based on the detected values of tag abundance in three different libraries, it appeared that a more active cleavage activity occurred in susceptible genotypes than in resistant genotypes. Notably, the same RPM homolog gene was identified as one of the downregulated R genes specifically in susceptible B.9 plants from a previous comparative transcriptome analysis (Zhu et al., 2019). Therefore, at transcriptional or post-transcriptional levels, a consistent regulation scheme seemed to corroborate each other.

**TABLE 6:** The genotype-specific expression of miR482c and target genes in response to *Pythium ultimum* infection of apple roots.

Genotype-specific expression: miR482c-known-3p-mature						
**Susceptible genotypes**						

	**Control**			***P. ultimum* infected**		
	
#106	1041.2	1291.5	1424.5	541.1	697.0	413.4
#115	1393.7	1260.4	1173.9	916.5	846.5	825.6
#132	1281.1	2224.9	1455.1	714.4	798.6	671.0
	**Ave (S_CK) = ^a^1394**			**Ave (S_Pu) = ^b^713.8**		

**Resistant genotypes**						

	**Control**			***P. ultimum* infected**		
	
#58	1341.2	1628.7	1368.5	560.3	724.2	585.8
#161	898.0	1054.3	1482.5	598.5	622.8	538.5
#164	1121.1	583.5	719.2	523.1	443.3	332.6
	**Ave (R_CK) = ^a^1133.0**			Ave (R_Pu) = ^b^547.7*		

**The identified target genes based on degradome sequencing:** Disease resistance protein RPM1 OS = *Arabidopsis thaliana* GN = RPM1 PE = 1 SV = 1 (*P*-value = 4.67E-100)						

**Cleavage Site**	**Target site sequence**			**Tag abundance**		
				L1	L2	L3
655	GGAAUGGGAGGCAUAGGCAAGA			9	N/A	5
3391	GGAAUGGGAGGAAUGGGGAAGA			N/A	N/A	112

*The values in the top section of the table, are the normalized read counts for each replicate per genotype/treatment. The average values are calculated for individual group (R vs. S), and under either mock-inoculation or *P. ultimum* infection. Different letters (a or b) in front of the average values indicate the significant difference between treatments (mock-inoculation and *P. ultimum* inoculation). Asterisks denote statistically significant differences in a one-tailed Mann–Whitney *U* test (^∗^*P* < 0.05) between genotype-groups (resistant and susceptible) of the same treatment (mock-inoculation or *P. ultimum* inoculation).*

The expression of miR10986 was generally detected at a low to moderate level in apple roots (Table 7), but a relatively large variation at read count values (2–3x) were observed among genotypes within the same R or S groups, or even within replicates for a given sample (genotype/treatment). Such uncommon variation among genotypes (within the same genotype group) probably indicated that its expression is also prone to certain abiotic stress conditions. In response to *P. ultimum* infection, downregulation was observed in all genotypes, but the degree of downregulation was more substantial (>1x) in resistant genotypes compared to that of susceptible genotypes. Similar to the regulation features for miR397, miR10986 also showed the lower basal expression in mock-inoculated tissues of R group as compared to that in susceptible genotypes. Degradome analysis indicated a polyphenol oxidase (PPO) is the cleavage target of miR10986. The overall cleavage activity on these PPO genes (for example, HF12100) was one of the strongest among the observed miRNA-target pairs from this dataset. However, a clear trend was missing, as the stronger cleavage activity was on either library L2 or L3 depending on the choices of cleavage sites. Therefore, the role of miR10986-PPO regulation during interaction between apple root and *P. ultimum* deserves future investigation.

**TABLE 7:** The genotype-specific expression of miR10986 and target genes in response to *Pythium ultimum* infection of apple roots.

Genotype-specific expression: miR10986 probable 5p mature						
**Susceptible genotypes**						

	**Control**			***P. ultimum* infected**		
	
#106	65.9	67.8	65.6	31.9	81.2	17.6
#115	208.1	267	271.5	138.7	157.3	161
#132	170.5	630.4	278.2	261.7	139.6	141.6
	**Ave (S_CK)** = **225**			**Ave (S_Pu)** = **125.6**		

**Resistant genotypes**						

	**Control**			***P. ultimum* infected**		
	
#58	223	260.7	232.1	101.4	111.4	108.3
#161	189.6	167.3	111.8	80	42.2	67.3
#164	68.8	58	50	39	42.2	34.1
	**Ave (R_CK)** = **^a^151.3**			**Ave (R_Pu)** = ^b^69.5*		

**The identified target genes based on degradome sequencing:** Polyphenol oxidase, chloroplastic OS = *Malus* × *domestica* PE = 2 SV = 1 (*P*-value = 0)						

**Cleavage site**	**Target site sequence**			**Tag abundance**		
				L1	L2	L3
1692	UUGGUGGUGACUUUGGUGCCG			92	N/A	N/A
1692	UUAGUGGUGACUUUGGUGCCA			1417	1426	724
930	UUGGUGGUGACUUUGGUGCCA			1390	1493	739
1686	UUGGUAGUGACUUUGGUGCCG			146	N/A	43
1752	CGUGGUGGUGACUUUGGUGCCC			105	139	154
1767	UGUGGUGGUGACUUUAGUGCCC			3	N/A	N/A

The values in the top section of the table are the normalized read counts for each replicate per genotype/treatment. The average values are calculated for individual group (R vs. S), and under either mock-inoculation or *P. ultimum* infection. Different letters (a or b) in front of the average values indicate the significant difference between treatments (mock-inoculation and *P. ultimum* inoculation). Asterisks denote statistically significant differences in a one-tailed Mann–Whitney *U* test (*P < 0.05) between genotype-groups (resistant and susceptible) of the same treatment (mock-inoculation or *P. ultimum* inoculation).

Both the mature and star forms of miR7122a were detected with comparable abundance in apple roots (Table 8). In susceptible genotypes, their expression levels were slightly downregulated due to *P. ultimum* infection (<25% for both mature and star form). In contrast, variable regulation patterns were exhibited among resistant genotypes, i.e., slight upregulated for its mature form (∼5%) and substantially downregulated (47.8%) for its star form. Analysis of degradome sequencing data demonstrated that a homolog to the “putative pentatricopeptide repeat (PPR)-containing protein At1g12700” was the target of miR7122a. Slightly elevated cleavage activities on this target gene occurred in the resistant genotypes (L2). The roles of PPR proteins in plant immunity have been reported (Schmitz-Linneweber and Small, 2008; Barkan and Small, 2014) in other pathosystems, the definitive contribution of this miR-target pair in shaping up the resistance traits in apple roots to *P. ultimum* infection deserve subsequent research.

**TABLE 8:** Genotype-specific expression of miR7122a-known-5p-mature, 3P-star, and identified target genes in apple roots in response to *Pythium ultimum* infection.

Genotype-specific expression: miR7122a-known-5p-mature and 3p-star							
**Susceptible genotypes**							

		**Control**			***P. ultimum* infected**		

#106	5p-mature	462.5	734.0	485.2	637.0	498.1	558.4
	3p-star	415.1	336.8	447.9	308.9	397.7	284.1
#115	5p-mature	1247.3	841.1	791.6	919.0	784.5	1216.9
	3p-star	698.1	638.5	568.4	802.6	673.7	803.2
#132	5p-mature	730.7	1022.6	760.6	236.0	414.2	402.2
	3p-star	774.8	1077.2	652.2	308.2	572.1	552.0

**Ave (S_CK)** = **786.2 (m)**					**Ave (S_Pu)** = **629.6 (m)**		

**Ave (S_CK)** = **623.2 (s)**					**Ave (S_Pu)** = **522.5 (s)**		

**Resistant genotypes**							

		**Control**			***P. ultimum* infected**		

#58	5p-mature	824.2	1604.0	817.0	1015.4	930.1	875.9
	3p-star	1261.9	1393.5	763.8	686.5	670.9	672.1
#161	5p-mature	945.4	891.1	1081.8	1127.4	1154.1	1139.6
	3p-star	634.6	635.0	835.3	521.9	511.9	477.6
#164	5p-mature	886.7	910.0	733.2	870.5	823.2	1189.3
	3p-star	688.0	546.7	545.0	513.3	453.8	431.6

**Ave (R_CK)** = 965.9 (m)*					**Ave (R_Pu)** = **1013.9 (m)**		

**Ave (R_CK)** = ^a^ 811.5 (s)**					**Ave (R_Pu)** = **^b^548.9 (s)**		

**The identified target gene based on degradome sequencing:** Putative pentatricopeptide repeat-containing protein At1g12700, mitochondrial OS = *Arabidopsis thaliana* GN = At1g12700 (*P-*value = 2.19E=-10)							

**Cleavage site**	**Target site sequence**				**Tag abundance**		

					L1	L2	L3
281	CGGCCGUGAUUUCUUUGUAUAA				N/A	187	162

The values in the top section of the table are the normalized read counts for each replicate per genotype/treatment. The average values are calculated for individual groups (R vs. S), and under either mock-inoculation or *P. ultimum* infection. Lower case letter “m” and “s” indicate the mature and star form of the miR7122a. Different letters (a or b) in front of the average values indicate the significant difference between treatments (mock-inoculation and *P. ultimum* inoculation). Asterisks denote statistically significant differences in a one-tailed Mann–Whitney *U* test (*P < 0.05) between genotype-groups (resistant and susceptible) of the same treatment (mock-inoculation or *P. ultimum* inoculation).

### PhasiRNA Analysis for miRNA390 and miRNA482/2118

**FIGURE 5:**
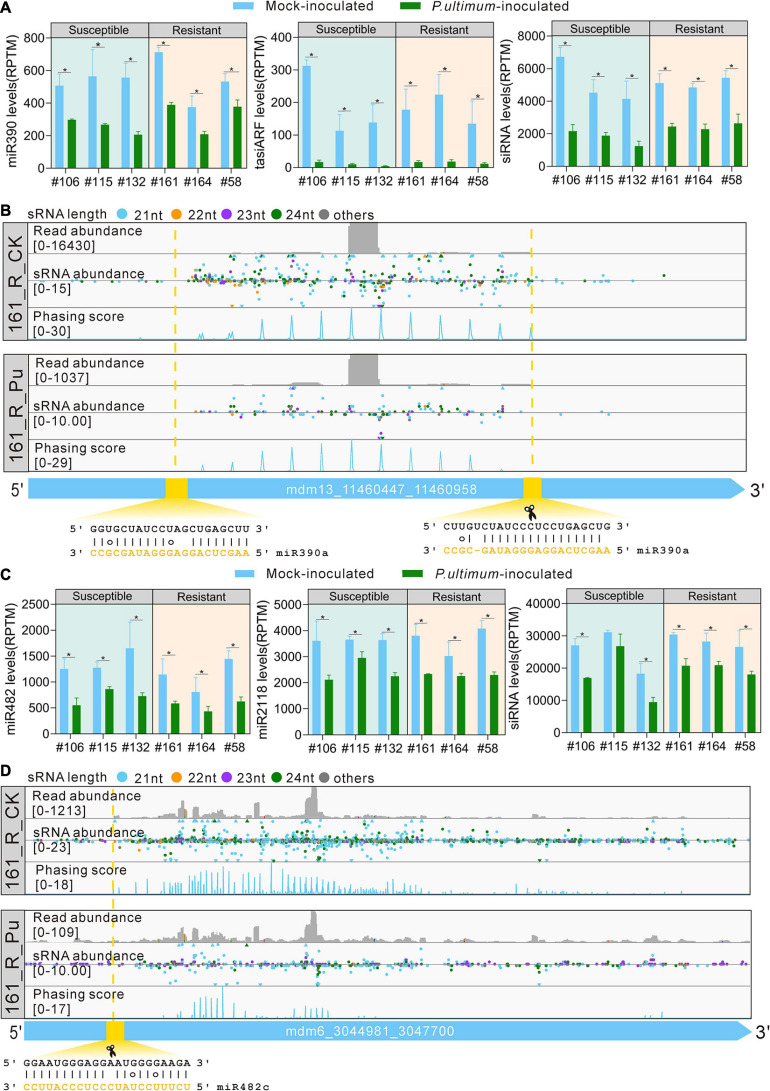
miRNA triggers phasiRNA biogenesis in response to *P. ultimum* inoculation. **(A)** The expression levels of miR390, tasiARF, and siRNA from TAS3 transcripts in mock-inoculated (blue bars) and *P. ultimum*-inoculated (green bars) root issues. **(B)** Read abundance, sRNA abundance and phasing score distribution of a TAS3 locus in mock-inoculated and *P. ultimum*-inoculated root issues were viewed in IGV-sRNA. The yellow dashed lines and rectangle indicate the target site and slicing site of miR390a. **(C)** The expression levels of miR482, miR2118, and siRNA from NBS-LRR transcripts (HF37161) in mock-inoculated (blue bars) and *P. ultimum*-inoculated (green bars) root tissues. **(D)** Read abundance, sRNA abundance, and phasing score distribution of a representative PHAS locus in mock-inoculated and *P. ultimum*-inoculated root tissues were viewed in IGV-sRNA. The yellow dashed lines and rectangle indicate the target site of miR482c. Phasing score was calculated based on the mapping results of 21-nucleotide siRNAs (Xia et al., 2013). Error bars represent SE of three independent biological replicates. Asterisks denote statistically significant differences in a one-tailed Mann–Whitney *U* test (*P < 0.05).

PhasiRNAs are another main class of small RNAs in plants. Interestingly, we found that overall production of phasiRNAs was significantly lower in the *P. ultimum*-inoculated root tissues for both susceptible and resistant materials, suggesting that phasiRNA pathway contributes to the defense to *P. ultimum*. miR390 triggers the production of trans-acting siRNA3 (TAS3)-derived tasiRNAs to repress auxin responsive factor 2/3/4 (ARF2/3/4) genes, critical for auxin signaling; these tasiRNAs are known as tasiARFs. In plants, 5′ proximal miR390 target site on TAS3 is non-cleavable while 3′ proximal target site is sufficient for miR390-directed slicing, leading to a “two-hit, one-cleavage” model (Xia et al., 2017). In this study, the expression levels of miR390, tasiARF and all siRNAs from TAS3 transcripts were all significantly downregulated in *P. ultimum*-inoculated root tissues (Figure 5A), indicating that auxin signaling module regulated by the miR390-TAS3-ARF pathway were turned down by the *P. ultimum* infection. Detailed examination of the mapping profile of a *TAS3* gene revealed that the miR390 cleavage site set the phase of the phasiRNA production, and indeed the abundance of sRNAs in mock-inoculated sample was much higher than that in *P. ultimum*-inoculated sample (Figure 5B).

MiR482/2118 is a well-known miRNA family important for disease resistance. All miRNA members of this family, including both miR482 and miR2118 variants are 22-nt long, and are capable to target NBS-LRR genes to trigger phasiRNAs biogenesis (Cuperus et al., 2010; Shivaprasad et al., 2012). In this study, a significant reduction on the expression levels of miR482, miR2118, and siRNAs from NBS-LRR transcripts were found in the *P. ultimum*-inoculated root tissues (Figure 5C), elucidating that miR482-mediated silencing of NBS-LRR genes was released in root tissues upon *P. ultimum* inoculation to activate defense. Detailed examination of the mapping profile of a NBS-LRR gene (R protein RPM1 homolog, HF37161) revealed that the miR482 cleavage site set the phase of the phasiRNA production and the abundance of phasiRNAs was greatly decreased in *P. ultimum*-inoculated roots (Figure 5D). These results regarding phasiRNAs suggested that apple trees turned down the endogenous sRNA silencing pathway to promote quick resistance response to the infection of *P. ultimum*. This kind of responses is likely widely present in plants.

### Genomic Location of miR397a in Apple Genome

**FIGURE 6:**
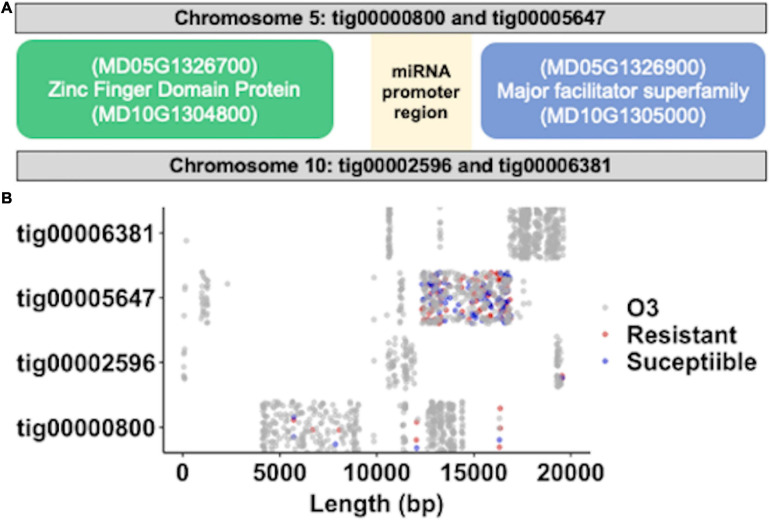
Genomic location of miR397a, expression variant analysis. **(A)** Illustration of physical map around MIR397a. The two regions on chromosome 5 and 10 that matches MIR397a containing contigs are shown. The relative upstream and downstream sequences from MIR397a position was used to identify the annotated genes including zinc finger domain protein and a major facilitator superfamily protein on the two apple chromosomes. **(B)** Distribution of variants across the four MIR397a matching “Robusta 5” contigs after its comparison with the “O3” parent (gray dots), resistance (red dots), and susceptible (blue dots) genotypes.

A comparison of miR397a precursor sequences against Robusta 5 *de novo* genome assembly identified four contig sequences (Figure 6A); two of which aligned to the same regions of chromosome 5 and chromosome 10, respectively, of the Golden Delicious Double Haploid genome (Daccord et al., 2017). The two overlapping contigs on same chromosomes represented the distinct haplotypes of the heterozygous apple genome. In addition, the regions of chromosomes 5 and 10 represent paralogs that were differentiated from a single ancestral chromosome through ancient whole genome duplication of the apple genome (Velasco et al., 2010). Annotation of 20 kb sequences around miR397a genomic loci identified a zinc-finger domain protein, an unknown protein, and a major facilitator superfamily protein. The unknown protein sequence appeared to match the promoter region of a miR397a gene from *Pyrus pyrifolia* (NCBI blast identifier: KY438936.1). Further comparison of Robusta 5 and Ottawa 3, across the miR397a targeted 20 kb genomic regions, identified 1,278 variants between them (Figure 6B). Co-linear contigs on chromosome 5 had relatively more variants than on chromosome 10 between Robusta 5 and Ottawa 3 on these regions. An alignment of *P. ultimum* susceptible (S) and resistant (R) genotype groups against Robusta 5 contigs resulted in 27 and 33 unique variants specific to each group, respectively (Figure 6B). A higher coverage of these regions with more sequencing reads can likely identify more variants in these regions.

## Discussion

Upon pathogen infection, plants activate a sophisticated defense system that initiates massive reprogramming of global gene expression. As an integral part of the host transcriptome themselves, sRNAs are versatile and important post-transcriptional regulators of gene expression in almost all cellular processes including plant-pathogen interactions (Staiger et al., 2013; Weiberg et al., 2014; Yang and Huang, 2014; Chaloner et al., 2016). These sRNAs negatively regulate gene expression by binding and cleavage of their specific target mRNAs, or repressing the translation process (Bartel, 2004; Axtell and Bowman, 2008). The current study is part of an integrated effort to define the resistance mechanisms in apple roots to infection from *P*. *ultimum*, through the identification of key miRNA families and their corresponding targets.

The majority of identified miRNA families exhibited downregulated expression patterns in response to *P. ultimum* infection, although variable degrees of downregulation existed between resistant and susceptible genotypes. It is apparent that most of these target genes positively regulate defense activation in apple roots, and upon pathogen infection the reduced or attenuated cleavage activities from corresponding miRNAs lead to elevated defense activation. On the other hand, there is a possibility that the pathogen-derived effectors, toxins, or even mobile sRNAs, effectively and non-selectively sabotaged host miRNA pathways and many other cellular processes (Qiao et al., 2013; de Vries et al., 2017; Hou et al., 2019). Therefore, existence of non-host (or passive) resistance, or certain forms of preformed defense systems, such as the chemo/physical barrier impeding the initial pathogen penetration, may be a critical contributor to resistance to this necrotrophic pathogen. The microscopic features of necrosis progression or arrested necrotic progression could partially owe to the enhanced tissue fortification which impede pathogen hyphae penetration at the initial stage of infection and enhance the plant survivability for those resistant genotypes (Zhu et al., 2018b).

In response to pathogenic pressure, an increased level of tissue fortification such as lignin deposition has been hypothesized as a fundamental resistance mechanism (Vance et al., 1980; Bhuiyan et al., 2009; Miedes et al., 2014; Yang et al., 2018). In this dataset, four miRNA families which belong to copper (Cu) microRNAs (Pilon, 2017), i.e., laccase-targeting miR397, *SOD*-targeting miR398, nudix hydrolase-targeting miR408, and *PPO*-targeting miR10986, were markedly downregulated in infected root tissue. The targets of these miRNAs are known to directly participate in abiotic and biotic stresses through increased H_2_O_2_ generation, enhanced lignin content and modified cell wall composition (Lu et al., 2013). The levels of downregulation for both miR397 and miR10986 were more obvious in resistant genotypes, than in susceptible genotypes. Interestingly, even in mock-inoculated root tissues of resistant apple rootstock genotypes, much lower levels of miR397a/b and miR10986 were observed. The elevated activities of laccase and PPO appear to exist prior to pathogen exposure in resistant genotypes, which likely function as a preformed defense mechanism. Upon pathogen challenge, the stronger downregulation of laccase-targeting miRNA was observed in resistant genotypes, possibly leading to a quicker and stronger tissue fortification for an effective impediment of pathogen progression at the initial stages of infection. As an important part of the inducible defense mechanisms, production of secondary metabolites such as phenolics from phenylpropanoid pathway and the formation of lignin are believed to be responsible for delay or stop of the establishment of pathogen infection in many pathosystems (Danielsson et al., 2011; Oliva et al., 2015). Gene expression regulation such as that from MYB TFs and microRNAs as well as biochemical feature such as secondary metabolites from phenylpropanoid pathway and defense phytohormones appear to be evolutionarily conserved from non-flowering land plants and even streptophyte algae to angiosperms (Overdijk et al., 2016; de Vries et al., 2018a; Carella et al., 2019). Future studies are needed to define the detailed functional roles of Cu-miRNAs in shaping up resistance traits in apple roots to *P. ultimum* infection.

Post-transcriptional regulation of R genes is known to be a crucial aspect of plant immune response (Staiger et al., 2013; Weiberg et al., 2014; Liu et al., 2017; Islam et al., 2018). The roles of miR482/2118 on the expression of NBS-LRRs in tomato (*Solanum lycopersicum*) during infection by *Phytophthora infestans* infection are well investigated; and overexpression of mir482c was shown to induce enhanced susceptibility to late blight in tomato (de Vries et al., 2018b; Jiang et al., 2018; Canto-Pastor et al., 2019; Hong et al., 2019). Interestingly, in addition to a decreased expression of target NBS-LRR genes, the transgenic tomato plants overexpressing miR482c also showed lower peroxidase (POD), superoxide dismutase (SOD), and phenylalanine ammonia-lyase (PAL) activities, indicating the interconnected immune responses (Hong et al., 2019). Our data revealed a consistent downregulation of miR482/miR2118 superfamily across genotypes, which indicated that regulation of R gene expression, through miRNA function, is essential for the resistance traits of apple roots to *P. ultimum* infection. A slightly higher level of downregulation appeared to be the case in infected roots of resistant genotypes. In many plant species, including perennial woody plants, the miRNA–NB-LRR interactions result in the production of phased, secondary small interfering (phasi)RNAs, which function in both *cis* and *trans*, to amplify the regulatory effects across additional members of the target gene family (Katiyar-Agarwal and Jin, 2010). Therefore, it is likely that the subtle variation of expression of miR482/miR2118 leads to amplified effect on phasiRNA biogenesis, which may become an important factor differentiating apple roots resistance vs. susceptibility.

Hormone signaling and their interactions with corresponding transcription factors (TFs) are the integrated modules regulating plant defense response, and both elements are the preferred targets of miRNA regulation (Curaba et al., 2014). At the same time, both miRNAs and TFs are the primary regulators of gene expression, often for the same target genes at transcriptional and/or post-transcriptional levels (Li et al., 2014; Samad et al., 2017). The complicated interactions among these regulatory components (miRNA-TF-hormone signaling) are the common theme in many pathosystems (Balmer and Mauch-Mani, 2013; Weiberg and Jin, 2015; Chaloner et al., 2016; Liu et al., 2017). From our dataset, miR393, miR160, and miR167, all of which target auxin signaling, were among a few of the identified miRNA families with mostly upregulated expression upon *P. ultimum* infection. Interestingly, miR160 showed partially differential regulation between R and S groups, where two out of three resistant genotypes exhibited downregulated expression in response to *P. ultimum* infection. Results from previous transcriptome analyses also identified regulatory components related to JA, auxin, WRKY, MYB, and NAC, which are largely consistent with the findings in the current study; (Shin et al., 2016b; Zhu et al., 2017, 2019). The role of these regulatory components in secondary metabolites including monolignols or the precursors of lignin deserves further investigation.

The identification of *bona fide* targets of a given miRNA species represents one of the fundamental aspects of small RNA research. Plant miRNAs can target multiple non-paralogous but functionally related genes (Djami-Tchatchou et al., 2017; You et al., 2017), which are often from a large gene family. Utilizing powerful degradome sequencing, a few target genes specific to this pathosystem were experimentally identified (rather than by *in silico* prediction). For example, three miRNA families (miR159, miR319, and miR858) are known to target a large number of MYB TFs in the apple genome, this approach pinpointed less than a dozen of MYB TF encoding genes which are potentially essential in this pathosystem. As an extreme example, only a single SPL encoding gene was identified as the target of miR156. In addition, novel targets were identified beyond the “commonly” established spectrum of genes functioning in this pathosystem such as those genes encoding PPRs (pentatricopeptide repeat-containing proteins). Most plants contain several hundred PPRs, the RNA-binding proteins with proposed functions of RNA editing, RNA splicing, RNA cleavage and translation within mitochondria and chloroplasts (Barkan and Small, 2014; Park et al., 2014) which has been linked to plant resistance response (Schmitz-Linneweber and Small, 2008; Barkan and Small, 2014). Moreover, the quantified “tag abundance” also provides the estimated miRNA cleavage activities between resistant or susceptible genotype groups. For example, the detected tag abundance for two target gene IDs “HF12099-RA and HF12100-RA” (polyphenol oxidase) were about 50% in infected susceptible root tissues (L3), compared to the values of mock-inoculated control tissues (L1), or infected resistant root tissues (L2). These identified target genes constitute a focused candidate gene list, which may significantly contribute to apple root resistance traits through post-transcriptional regulations.

As part of the integrated effort to uncover the underpinned molecular mechanism controlling apple roots resistance to *P. ultimum*, the current dataset of miRNA profiling and degradome sequencing offers a unique perspective on its post-transcriptional regulation. The identified “miRNA-target gene” pairs likely represent the crucial components functioning in apple root defense activation to *P. ultimum* infection. As examples, genotype-specific expression patterns for miR397-laccse, miR10986-PPO, and miR398-SOD appeared to link with apple root resistance vs. susceptibility. Cell wall fortification through the function of copper protein encoding genes could be a key strategy to defend apple roots against this necrotrophic pathogen. Young apple roots, as a primary organ for taking up water and nutrients, lack effective protection layers such as cuticle or wax in aerial parts of a plant to deter or impede penetration from pathogen like *P. ultimum.* Winning the chemical war at the early stage of infection is crucial to thwart disruptive arsenals of effectors and toxins from this fast-growing necrotrophic pathogen. Therefore, quick and effective cell wall modification through the function of these Cu-miRNAs (Pilon, 2017) can significantly determine the outcome between the apple roots and *P. ultimum*. Such initial defense reactions are likely “buying the time” for a steady and robust defense activation to be developed. Inclusion of multiple genotypes with contrasting resistance phenotypes enhanced the reliability of this comprehensive dataset from this study. Those miRNA families targeting R genes, hormonal signaling, and various TFs represent the core regulation circuits for this pathosystem. Noticeably, many of the identified target genes in the current study are consistent with the results from previous transcriptome analyses (Shin et al., 2016b; Zhu et al., 2017, 2019). As an example, the same RPM1 homolog gene was also identified from a susceptible genotype B.9 with downregulated expression (Zhu et al., 2019). In summary, the identified miRNA species and their corresponding target genes constitute a valuable resource for subsequent functional analysis to define their detailed roles in shaping up the resistance traits in apple roots against *P. ultimum* infection. The findings from this study significantly contribute to the effort for deciphering the molecular mechanisms controlling defense responses for this less explored pathosystem between apple roots and a necrotrophic pathogen.

## Data Availability Statement

The datasets presented in this study can be found in online repositories. The names of the repository/repositories and accession number(s) can be found in the article/Supplementary Material.

## Author Contributions

YZ, RX, AK, and GF participated in the experimental design, data analysis, interpretations, and manuscript writing. MS performed most of the experiments of phenotyping and RNA preparation. GL and JS analyzed the dataset and contributed to the manuscript preparation. All authors contributed to the article and approved the submitted version.

## Conflict of Interest

The authors declare that the research was conducted in the absence of any commercial or financial relationships that could be construed as a potential conflict of interest.

## Publisher’s Note

All claims expressed in this article are solely those of the authors and do not necessarily represent those of their affiliated organizations, or those of the publisher, the editors and the reviewers. Any product that may be evaluated in this article, or claim that may be made by its manufacturer, is not guaranteed or endorsed by the publisher.

## Footnotes

1
www.faststone.org

2
https://www.rosaceae.org/species/malus_x_domestica_HFTH1/genome_v1.0

3
ncbi.nlm.nih.gov/sra/review/SRP295189

4
http://www.rosaceae.org

5
www.bioinformatics.nl/cgi-bin/primer3plus/primer3plus.cgi

6
www.idtdna.com/analyzer/Applications/OligoAnalyzer/

7
https://www.rosaceae.org/analysis/162
