# Supplementary material for: Laccase Directed Lignification Is One of the Major Processes Associated With the Defense Response Against Pythium ultimum Infection in Apple Roots
Source: Front Plant Sci. 2021 Sep 7;12:629776. doi: 10.3389/fpls.2021.629776 (PMC8453155; doi:10.3389/fpls.2021.629776)
Supplement: Supplementary file 1 [file Data_Sheet_1.PDF]

GeneID	106_S_CK_rep	106_S_CK_r	106_S_CK_r	106_S_Pu_i	106_S_Pu_i	106_S_Pu_i
miR10980-Probable-3p-mature	0.479053654	5.7474759	2.9493187	0	4.2759566	1.9592926
miR10980-Probable-5p-star	0	0	0	0	0	0
miR10984-Known-3p-mature	504.6830249	462.09706	652.53675	255.6552	312.14483	203.76643
miR10984-Known-5p-star	24.43173638	22.70253	28.755857	10.6523	10.689891	15.674341
miR10986-Probable-3p-star	0	0	0	0	0	0
miR10986-Probable-5p-mature	65.86987748	67.820216	65.62234	31.9569	81.243175	17.633633
miR10991a-Probable-3p-star	0	0	0	0	0	0
miR10991a-Probable-5p-mature	0.239526827	0	0.3686648	0	2.1379783	0
miR10991b-Probable-3p-star	0	0	0	0	0	0
miR10991b-Probable-5p-mature	0.239526827	0	0.3686648	0	2.1379783	0
miR10996-Known-3p-mature	90.06208703	122.42124	92.903537	38.34828	42.759566	60.738069
miR10996-Known-5p-star	0.479053654	2.0116166	0.7373297	0	0	0
miR11014-Probable-3p-mature	8.622965779	9.1959615	11.42861	4.26092	4.2759566	1.9592926
miR11014-Probable-5p-star	0	0	0	0	0	0
miR1511a-Known-3p-mature	16.52735108	25.00152	23.963214	6.39138	8.5519131	13.715048
miR1511a-Known-5p-star	0.239526827	0.2873738	0	0	0	0
miR1511b-Probable-3p-mature	15.32971694	16.380306	16.589917	4.26092	4.2759566	3.9185851
miR1511b-Probable-5p-star	0	0	1.4746593	0	0	0
miR1511c-Probable-3p-star	0	0	0	0	0	0
miR1511c-Probable-5p-mature	2.634795099	2.0116166	2.5806538	0	0	1.9592926
miR1511d-Known-3p-mature	19554.25159	22563.728	21591.593	7697.352	7636.8584	7686.3047
miR1511d-Known-5p-star	15.09019011	19.541418	7.7419615	8.52184	2.1379783	7.8371702
miR1511e-Probable-3p-mature	523.3661175	575.03496	579.54111	264.17704	299.31696	190.05138
miR1511e-Probable-5p-star	0	0	0	0	0	0
miR1526-Known-3p-mature	6.227697507	10.63283	4.7926428	0	0	5.8778777
miR1526-Known-5p-star	0.239526827	0.2873738	0	0	0	0
miR156a-Known-3p-star	0.479053654	0.8621214	0.3686648	0	0	0
miR156a-Known-5p-mature	3.113848754	1.1494952	3.6866483	0	0	0
miR156b-Probable-3p-mature	9.820599916	9.770709	12.165939	10.6523	8.5519131	1.9592926
miR156b-Probable-5p-star	0	0	0	0	0	0
miR156c-Known-3p-star	62.27697507	71.843449	89.585554	55.39196	79.105196	48.982314
miR156c-Known-5p-mature	**252.9403295**	320.42178	281.29127	136.34944	224.48772	139.10977
miR156d-Probable-3p-star	0	0.5747476	0	2.13046	0	0
miR156d-Probable-5p-mature	1.916214618	2.873738	3.3179835	0	2.1379783	0
miR156e-Known-3p-star	28.74321926	34.197482	27.649862	42.6092	42.759566	29.389388
miR156e-Known-5p-mature	**1236.437482**	1607.2816	1545.0743	1039.6645	1272.0971	899.31529
miR156f-Known-3p-star	48.86347275	58.624254	43.50245	53.2615	62.00137	39.185851
miR156f-Known-5p-mature	1002.180245	1116.4472	1269.6817	715.83456	739.74048	632.8515
miR156g-Known-3p-star	62.27697507	71.843449	89.585554	55.39196	79.105196	48.982314
miR156g-Known-5p-mature	254.1379637	322.14602	284.97791	136.34944	224.48772	144.98765
miR156h-Known-3p-mature	**1612.734128**	1795.5115	1838.5315	472.96212	575.11616	444.75941
miR156h-Known-5p-star	127.9073257	144.54902	125.34604	55.39196	102.62296	62.697362
miR156i-Known-3p-star	9.341546261	9.1959615	11.797275	10.6523	8.5519131	1.9592926
miR156i-Known-5p-mature	127.9073257	144.54902	125.34604	55.39196	102.62296	62.697362
miR156j-Known-3p-star	28.74321926	34.197482	27.649862	42.6092	42.759566	29.389388
miR156j-Known-5p-mature	**1236.437482**	1607.2816	1545.0743	1039.6645	1272.0971	899.31529

miR156k-Known-3p-star	48.86347275	58.624254	43.50245	53.2615	62.00137	39.185851
miR156k-Known-5p-mature	84.79249683	93.396484	98.43351	66.04426	83.381153	50.941607
miR156l-Known-3p-mature	0.958107309	0.8621214	1.1059945	0	0	1.9592926
miR156l-Known-5p-star	1.916214618	2.873738	3.3179835	0	2.1379783	0
miR156m-Probable-3p-star	0	0	0	0	0	0
miR156m-Probable-5p-mature	3.832429235	3.4484855	2.5806538	0	0	1.9592926
miR156n-Known-3p-star	48.86347275	58.624254	43.50245	53.2615	62.00137	39.185851
miR156n-Known-5p-mature	1002.180245	1116.4472	1269.6817	715.83456	739.74048	632.8515
miR156o-Known-3p-star	62.27697507	71.843449	89.585554	55.39196	79.105196	48.982314
miR156o-Known-5p-mature	**252.9403295**	320.42178	281.29127	136.34944	224.48772	139.10977
miR156p-Known-3p-star	222.2808956	271.28086	302.67383	151.26266	213.79783	101.88321
miR156p-Known-5p-mature	127.9073257	144.54902	125.34604	55.39196	102.62296	62.697362
miR156q-Known-3p-star	221.801842	268.98187	301.19917	151.26266	213.79783	101.88321
miR156q-Known-5p-mature	127.9073257	144.54902	125.34604	55.39196	102.62296	62.697362
miR156r-Probable-3p-star	0	0	0	0	0	0
miR156r-Probable-5p-mature	**252.9403295**	320.42178	281.29127	136.34944	224.48772	139.10977
miR156s-Probable-3p-star	0	0	0	0	0	0
miR156s-Probable-5p-mature	**252.9403295**	320.42178	281.29127	136.34944	224.48772	139.10977
miR156t-Known-3p-star	2.395268272	4.0232331	6.2673021	2.13046	4.2759566	5.8778777
miR156t-Known-5p-mature	**252.9403295**	320.42178	281.29127	136.34944	224.48772	139.10977
miR156u-Known-3p-star	3.832429235	3.1611118	2.5806538	0	4.2759566	0
miR156u-Known-5p-mature	127.9073257	144.83639	125.71471	55.39196	102.62296	62.697362
miR156v-Probable-3p-mature	3.832429235	3.7358593	2.211989	0	0	0
miR156v-Probable-5p-star	0	0	0	0	0	0
miR159a-Known-3p-mature	15546.72825	17049.6	19755.274	8242.7497	10080.568	7110.2727
miR159a-Known-5p-star	147.3089987	155.46922	316.68309	55.39196	76.967218	43.104436
miR159b-Probable-3p-mature	53.17495564	70.981327	70.783648	14.91322	8.5519131	5.8778777
miR159b-Probable-5p-star	0	0	0	0	0	0
miR159c-Known-3p-mature	15546.72825	17049.6	19755.274	8242.7497	10080.568	7110.2727
miR159c-Known-5p-star	147.3089987	155.46922	316.68309	55.39196	76.967218	43.104436
miR160a-Known-3p-star	10.06012674	19.541418	16.589917	0	4.2759566	0
miR160a-Known-5p-mature	217.0113055	232.77277	263.96402	266.3075	414.76779	299.77176
miR160b-Known-3p-star	23.23410224	34.197482	39.815802	10.6523	12.82787	5.8778777
miR160b-Known-5p-mature	218.4484664	234.20964	265.07001	270.56842	414.76779	299.77176
miR160c-Known-3p-star	22.51552176	31.03637	31.705175	4.26092	6.4139348	11.755755
miR160c-Known-5p-mature	217.0113055	232.77277	263.96402	266.3075	414.76779	299.77176
miR160d-Known-3p-star	4.071956063	7.4717187	5.5299725	2.13046	0	0
miR160d-Known-5p-mature	218.4484664	234.20964	265.07001	270.56842	414.76779	299.77176
miR162a-Known-3p-mature	1040.744064	1317.0341	1379.9125	675.35582	829.53557	679.87452
miR162a-Known-5p-star	65.151297	101.73032	110.59945	55.39196	57.725413	54.860192
miR162b-Known-3p-mature	1040.744064	1317.0341	1379.9125	675.35582	829.53557	679.87452
miR162b-Known-5p-star	65.151297	101.73032	110.59945	55.39196	57.725413	54.860192
miR164a-Known-3p-star	3.592902408	1.436869	6.2673021	0	0	3.9185851
miR164a-Known-5p-mature	259.4075539	229.03692	179.53977	78.82702	160.34837	101.88321
miR164b-Known-3p-star	238.0896662	292.54652	353.91824	125.69714	171.03826	99.923921
miR164b-Known-5p-mature	259.4075539	229.61166	179.53977	78.82702	***	101.88321
miR164c-Known-3p-star	0.479053654	0.2873738	0	0	0	0

miR164c-Known-5p-mature	259.4075539	229.03692	179.53977	78.82702	160.34837	101.88321
miR164d-Known-3p-mature	2.634795099	2.5863642	1.8433242	0	0	0
miR164d-Known-5p-star	1.197634136	2.0116166	2.9493187	2.13046	0	0
miR164e-Known-3p-mature	7.904385298	10.345457	11.059945	0	4.2759566	1.9592926
miR164e-Known-5p-star	0	0.2873738	0	0	0	0
miR164f-Known-3p-star	238.0896662	292.54652	353.91824	125.69714	171.03826	99.923921
miR164f-Known-5p-mature	259.4075539	229.61166	179.53977	78.82702	160.34837	101.88321
miR164g-Probable-3p-star	0	0	0	0	0	0
miR164g-Probable-5p-mature	259.4075539	229.03692	179.53977	78.82702	160.34837	101.88321
miR164h-Probable-3p-star	0.479053654	0	0	0	0	0
miR164h-Probable-5p-mature	3.353375581	3.1611118	4.7926428	0	0	3.9185851
miR166a-Known-3p-mature	642299.3311	571747.12	555352.28	198754.87	263238.58	208768.5
miR166a-Known-5p-star	1119.308864	1015.579	1243.5065	306.78624	329.24865	297.81247
**miR166b-Known-3p-mature**	**642561.1339**	**571743.39**	**555276.33**	**198904.01**	**263356.16**	**208860.59**
miR166b-Known-5p-star	1.916214618	7.1843449	4.0553131	2.13046	2.1379783	1.9592926
miR166c-Known-3p-mature	642570.9545	571812.93	555258.64	198889.09	263334.78	208858.63
miR166c-Known-5p-star	54.85164343	53.451526	59.355038	51.13104	49.1735	35.267266
miR166d-Known-3p-mature	642299.3311	571747.12	555352.28	198754.87	263238.58	208768.5
miR166d-Known-5p-star	507.7968737	550.89557	503.22749	168.30634	218.07378	143.02836
miR166e-Known-3p-mature	642299.3311	571747.12	555352.28	198754.87	263238.58	208768.5
miR166e-Known-5p-star	1125.776088	1020.177	1247.1931	308.9167	329.24865	301.73105
miR166f-Known-3p-mature	19860.12735	18874.998	21012.789	9742.5936	16379.052	8405.3651
miR166f-Known-5p-star	0.718580482	1.1494952	1.4746593	0	0	0
**miR166g-Known-3p-mature**	**642561.1339**	**571743.39**	**555276.33**	**198904.01**	**263356.16**	**208860.59**
miR166g-Known-5p-star	0.239526827	0.8621214	0.7373297	0	0	0
miR166h-Probable-3p-mature	642601.1349	571746.83	555278.18	198863.53	263373.27	208882.14
miR166h-Probable-5p-star	0	0.2873738	0	0	0	0
miR166i-Known-3p-mature	642601.1349	571746.83	555278.18	198863.53	263373.27	208882.14
miR166i-Known-5p-star	31.13848754	34.772229	33.179835	8.52184	10.689891	5.8778777
miR166j-Probable-3p-mature	642611.6741	571818.1	555259.74	198848.61	263347.61	208880.18
miR166j-Probable-5p-star	0	0.2873738	0	0	0	0
miR167a-Known-3p-star	12.93444867	12.357073	8.847956	6.39138	0	0
miR167a-Known-5p-mature	155.213384	194.55206	164.79318	302.52532	327.11068	315.4461
miR167b-Known-3p-star	0.239526827	0.5747476	0.7373297	0	0	0
miR167b-Known-5p-mature	137.7279256	175.29802	145.99127	298.2644	312.14483	299.77176
miR167c-Known-3p-star	1.67668779	1.7242428	0.7373297	0	0	0
miR167c-Known-5p-mature	154.9738572	193.40256	165.16184	302.52532	324.9727	315.4461
miR167d-Known-3p-star	12.93444867	12.357073	8.847956	6.39138	0	0
miR167d-Known-5p-mature	155.213384	194.55206	164.79318	302.52532	327.11068	315.4461
miR167e-Known-3p-star	2.395268272	4.0232331	2.5806538	2.13046	6.4139348	0
miR167e-Known-5p-mature	137.7279256	175.29802	145.99127	298.2644	312.14483	299.77176
miR167f-Known-3p-star	12.93444867	12.357073	8.847956	6.39138	0	0
miR167f-Known-5p-mature	154.9738572	193.40256	165.16184	302.52532	324.9727	315.4461
miR167g-Known-3p-star	1.916214618	3.4484855	2.211989	0	0	0
miR167g-Known-5p-mature	57.72596536	57.187385	49.401087	219.43738	222.34974	201.80713
miR167h-Known-3p-star	1.916214618	3.4484855	2.211989	0	0	0
miR167h-Known-5p-mature	57.72596536	57.187385	49.401087	219.43738	222.34974	201.80713

miR168a-Known-3p-star	2323.410224	2904.487	4279.4614	950.18516	1231.4755	697.50815
miR168a-Known-5p-mature	5454.025856	5434.5258	8608.3238	2573.5957	3016.6873	2437.3599
miR168b-Known-3p-star	2323.410224	2904.487	4279.4614	950.18516	1231.4755	697.50815
miR168b-Known-5p-mature	5454.025856	5434.5258	8608.3238	2571.4652	3016.6873	2437.3599
miR169a-Known-3p-star	0	0	0	0	0	0
miR169a-Known-5p-mature	20.83883397	21.553035	8.4792911	6.39138	17.103826	25.470803
miR169b-Known-3p-star	1.197634136	1.7242428	2.211989	0	2.1379783	0
miR169b-Known-5p-mature	20.35978031	19.828792	8.4792911	6.39138	17.103826	23.511511
miR169c-Known-3p-mature	0.718580482	0.8621214	1.4746593	0	0	0
miR169c-Known-5p-star	1.197634136	0.5747476	0	0	0	0
miR169d-Known-3p-star	1.437160963	2.5863642	1.8433242	4.26092	8.5519131	7.8371702
miR169d-Known-5p-mature	581.3316096	526.75617	461.93703	234.3506	320.69674	356.59125
miR169e-Known-3p-star	0	0	0	0	0	0
miR169e-Known-5p-mature	20.83883397	21.840408	8.4792911	6.39138	17.103826	25.470803
miR169f-Probable-3p-star	0	0	0	0	0	0
miR169f-Probable-5p-mature	7.185804816	11.494952	14.377928	8.52184	10.689891	5.8778777
miR169g-Known-3p-star	1.197634136	3.7358593	4.423978	0	4.2759566	0
miR169g-Known-5p-mature	20.83883397	21.553035	8.4792911	6.39138	17.103826	25.470803
miR169h-Known-3p-star	0.239526827	1.1494952	0	4.26092	2.1379783	1.9592926
miR169h-Known-5p-mature	20.35978031	19.828792	8.4792911	6.39138	17.103826	23.511511
miR169i-Known-3p-star	1.197634136	3.7358593	4.423978	0	4.2759566	0
miR169i-Known-5p-mature	20.83883397	21.553035	8.4792911	6.39138	17.103826	25.470803
miR169j-Known-3p-star	2.395268272	1.7242428	3.3179835	4.26092	8.5519131	9.7964628
miR169j-Known-5p-mature	7.185804816	11.494952	14.377928	8.52184	10.689891	5.8778777
miR169k-Known-3p-star	0	0	0	0	0	1.9592926
miR169k-Known-5p-mature	20.83883397	21.553035	8.4792911	6.39138	17.103826	25.470803
miR169l-Probable-3p-star	0	0	0	0	0	0
miR169l-Probable-5p-mature	7.185804816	11.494952	14.377928	8.52184	10.689891	5.8778777
miR171a-Known-3p-mature	221.0832615	320.13441	227.09754	93.74024	72.691261	74.453117
miR171a-Known-5p-star	0.479053654	0.8621214	0.7373297	4.26092	6.4139348	9.7964628
miR171b-Known-3p-mature	113.2961893	130.75508	123.87138	80.95748	96.209022	39.185851
miR171b-Known-5p-star	10.29965357	18.104549	9.2166208	6.39138	6.4139348	3.9185851
miR171c-Known-3p-mature	48.3844191	62.07274	65.991005	14.91322	12.82787	29.389388
miR171c-Known-5p-star	55.80975074	47.129302	58.249043	10.6523	6.4139348	9.7964628
miR171d-Probable-3p-mature	61.07934094	68.682337	71.889642	44.73966	76.967218	47.023021
miR171d-Probable-5p-star	0	0	0.3686648	0	0	0
miR171e-Known-3p-mature	47.42631179	59.199002	64.147681	14.91322	12.82787	29.389388
miR171e-Known-5p-star	12.69492184	12.069699	15.115258	2.13046	2.1379783	3.9185851
miR171f-Known-3p-mature	113.2961893	130.75508	123.87138	80.95748	96.209022	39.185851
miR171f-Known-5p-star	10.29965357	18.104549	9.2166208	6.39138	6.4139348	3.9185851
miR171g-Known-3p-mature	61.31886777	68.969711	72.258307	44.73966	76.967218	47.023021
miR171g-Known-5p-star	0	0	0	0	0	0
miR172a-Probable-3p-mature	2.634795099	3.4484855	2.211989	2.13046	2.1379783	3.9185851
miR172a-Probable-5p-star	0	0	0	0	0	0
miR172b-Known-3p-star	1.916214618	1.1494952	2.211989	4.26092	2.1379783	5.8778777
miR172b-Known-5p-mature	3.832429235	4.8853545	5.5299725	0	2.1379783	1.9592926
miR172c-Known-3p-mature	2.634795099	3.4484855	2.211989	2.13046	2.1379783	3.9185851

miR172c-Known-5p-star	0.239526827	0.8621214	0	0	0	0
miR172d-Probable-3p-mature	1.916214618	1.1494952	2.211989	4.26092	2.1379783	5.8778777
miR172d-Probable-5p-star	0	0	0	0	0	0
miR172e-Probable-3p-mature	1.916214618	1.1494952	2.211989	4.26092	2.1379783	5.8778777
miR172e-Probable-5p-star	0	0	0	0	0	0
miR172f-Probable-3p-mature	2.634795099	3.7358593	2.211989	2.13046	2.1379783	3.9185851
miR172f-Probable-5p-star	0	0	0	0	0	0
miR172g-Probable-3p-mature	1.916214618	1.1494952	2.211989	4.26092	2.1379783	5.8778777
miR172g-Probable-5p-star	0	0	0	0	0	0
miR2111a-Known-3p-star	9.820599916	8.6212139	14.746593	6.39138	6.4139348	1.9592926
miR2111a-Known-5p-mature	5.030063371	9.770709	11.42861	10.6523	4.2759566	0
miR2111b-Probable-3p-mature	5.030063371	9.770709	11.42861	10.6523	4.2759566	0
miR2111b-Probable-5p-star	0	0	0	0	0	0
miR2111c-Probable-3p-star	0	0	0	0	0	0
miR2111c-Probable-5p-mature	5.030063371	9.770709	11.42861	10.6523	4.2759566	0
miR2118a-Known-3p-mature	2836.476688	3657.4063	4338.0791	2143.2428	2276.9469	1924.0253
miR2118a-Known-5p-star	17.96451204	15.230811	18.433242	6.39138	12.82787	7.8371702
miR2118b-Known-3p-mature	2836.476688	3657.4063	4338.0791	2143.2428	2276.9469	1924.0253
miR2118b-Known-5p-star	15.32971694	12.069699	12.165939	2.13046	12.82787	1.9592926
miR319a-Known-3p-mature	25850.6933	33952.352	36699.847	14218.69	15228.819	13235.021
miR319a-Known-5p-star	85.99013097	132.47932	188.01906	25.56552	38.483609	11.755755
miR319b-Known-3p-mature	567.6785805	634.80871	698.98852	283.35118	318.55876	213.56289
miR319b-Known-5p-star	10.06012674	10.63283	10.322615	8.52184	2.1379783	7.8371702
miR319c-Probable-3p-mature	84.79249683	95.982848	108.0188	61.78334	32.069674	41.145144
miR319c-Probable-5p-star	0	0	0	0	0	0
miR319d-Known-3p-mature	917.8668019	1098.63	1195.58	351.5259	303.59292	333.07974
miR319d-Known-5p-star	2.874321926	2.2989904	1.4746593	0	2.1379783	1.9592926
miR319e-Known-3p-mature	910.4414702	1087.7098	1185.9948	347.26498	303.59292	333.07974
miR319e-Known-5p-star	2.395268272	0.8621214	1.4746593	0	2.1379783	0
miR319f-Known-3p-mature	25912.97027	34020.172	36775.792	14237.864	15256.613	13252.655
miR319f-Known-5p-star	14.61113646	19.828792	17.695912	4.26092	14.965848	1.9592926
miR319g-Known-3p-mature	567.6785805	634.80871	698.98852	283.35118	318.55876	213.56289
miR319g-Known-5p-star	5.748643853	4.8853545	5.8986373	2.13046	4.2759566	5.8778777
miR319h-Known-3p-mature	917.8668019	1098.63	1195.58	351.5259	303.59292	333.07974
miR319h-Known-5p-star	3.113848754	2.2989904	1.8433242	0	2.1379783	1.9592926
miR319i-Probable-3p-star	0	0	0	0	0	0
miR319i-Probable-5p-mature	496.7786396	556.93042	607.92831	255.6552	288.62707	182.21421
miR319j-Known-3p-mature	910.4414702	1087.7098	1185.9948	347.26498	303.59292	333.07974
miR319j-Known-5p-star	5.748643853	4.8853545	5.8986373	2.13046	4.2759566	5.8778777
miR319k-Probable-3p-mature	496.7786396	556.93042	607.92831	255.6552	288.62707	182.21421
miR319k-Probable-5p-star	0	0	0	0	0	0
miR3627a-Known-3p-star	2.634795099	2.873738	2.5806538	0	4.2759566	3.9185851
miR3627a-Known-5p-mature	10.5391804	12.069699	9.2166208	14.91322	14.965848	7.8371702
miR3627b-Known-3p-star	3.592902408	0.8621214	4.0553131	0	0	1.9592926
miR3627b-Known-5p-mature	33.05470215	37.07122	22.11989	25.56552	51.311479	31.348681
miR3627c-Known-3p-star	2.395268272	3.1611118	2.5806538	8.52184	4.2759566	1.9592926
miR3627c-Known-5p-mature	33.05470215	37.07122	22.11989	25.56552	51.311479	31.348681

miR3627d-Known-3p-star	0.239526827	0	0	0	0	0
miR3627d-Known-5p-mature	33.05470215	37.07122	22.11989	25.56552	51.311479	31.348681
miR390a-Known-3p-star	18.92261935	18.391923	22.488555	8.52184	12.82787	5.8778777
miR390a-Known-5p-mature	427.3158597	531.06677	564.05719	291.87302	301.45494	299.77176
miR390b-Known-3p-star	19.16214618	27.875258	27.649862	27.69598	23.517761	27.430096
miR390b-Known-5p-mature	427.3158597	532.21627	564.42586	291.87302	299.31696	299.77176
miR390c-Known-3p-star	18.92261935	18.391923	22.488555	8.52184	12.82787	5.8778777
miR390c-Known-5p-mature	427.3158597	531.35415	564.42586	291.87302	301.45494	299.77176
miR390d-Known-3p-star	18.92261935	18.391923	22.488555	8.52184	12.82787	5.8778777
miR390d-Known-5p-mature	427.3158597	532.21627	564.42586	291.87302	299.31696	299.77176
miR391-Probable-3p-star	0.239526827	0.2873738	0	0	0	0
miR391-Probable-5p-mature	9.102019434	8.0464663	7.3732966	8.52184	4.2759566	9.7964628
miR393a-Known-3p-star	6.706751162	10.920204	8.847956	6.39138	8.5519131	3.9185851
miR393a-Known-5p-mature	20.35978031	35.059603	34.285829	25.56552	34.207652	27.430096
miR393b-Known-3p-star	2.634795099	2.0116166	1.4746593	0	0	0
**miR393b-Known-5p-mature**	**131.2607013**	**203.74802**	**183.96375**	**187.48048**	**218.07378**	**188.09209**
miR393c-Known-3p-star	6.706751162	10.920204	8.847956	6.39138	8.5519131	3.9185851
miR393c-Known-5p-mature	20.35978031	35.059603	34.285829	25.56552	34.207652	27.430096
miR393d-Known-3p-star	2.634795099	2.0116166	1.4746593	0	0	0
**miR393d-Known-5p-mature**	**131.2607013**	**203.74802**	**183.96375**	**187.48048**	**218.07378**	**188.09209**
miR395a-Known-3p-mature	59.64217997	48.566171	53.087736	38.34828	64.139348	19.592926
miR395a-Known-5p-star	1.437160963	1.436869	0	0	0	0
miR395b-Probable-3p-mature	4.31148289	7.1843449	6.635967	4.26092	17.103826	5.8778777
miR395b-Probable-5p-star	0	0	0	0	0	0
miR395c-Known-3p-mature	59.64217997	48.566171	53.087736	38.34828	64.139348	19.592926
miR395c-Known-5p-star	20.12025349	22.989904	22.11989	6.39138	23.517761	5.8778777
miR395d-Probable-3p-star	0.479053654	0.2873738	0	0	0	0
miR395d-Probable-5p-mature	20.12025349	22.989904	22.11989	6.39138	23.517761	5.8778777
miR395e-Known-3p-mature	58.20501901	48.566171	51.244412	38.34828	64.139348	19.592926
miR395e-Known-5p-star	20.12025349	22.989904	22.11989	6.39138	23.517761	5.8778777
miR395f-Known-3p-mature	59.64217997	48.566171	53.087736	38.34828	64.139348	19.592926
miR395f-Known-5p-star	0	0.5747476	0.7373297	0	0	0
miR395g-Probable-3p-mature	58.44454584	47.991424	51.244412	38.34828	64.139348	19.592926
miR395g-Probable-5p-star	0	0	0	0	0	0
miR395h-Probable-3p-mature	4.551009717	8.9085877	8.4792911	6.39138	21.379783	5.8778777
miR395h-Probable-5p-star	0	0	0.3686648	0	0	0
miR395i-Known-3p-mature	58.44454584	48.566171	51.244412	38.34828	64.139348	19.592926
miR395i-Known-5p-star	20.12025349	22.989904	22.11989	6.39138	23.517761	5.8778777
miR395j-Known-3p-mature	59.64217997	49.140919	53.087736	38.34828	64.139348	19.592926
miR395j-Known-5p-star	20.12025349	22.989904	22.11989	6.39138	23.517761	5.8778777
miR395k-Known-3p-mature	59.64217997	48.566171	53.087736	38.34828	64.139348	19.592926
miR395k-Known-5p-star	0	0	0	2.13046	0	0
miR396a-Known-3p-star	232.5805492	302.31723	297.14385	104.39254	128.2787	105.8018
miR396a-Known-5p-mature	4640.353224	5872.7709	6837.258	4993.7982	5744.7476	5393.9324
miR396b-Known-3p-star	3.592902408	6.6095973	4.423978	4.26092	4.2759566	0
miR396b-Known-5p-mature	5040.602552	4331.2978	4931.6294	3805.0016	4213.9552	4716.0172
miR396c-Probable-3p-star	0	0	0	0	0	0

miR396c-Probable-5p-mature	1443.867714	1526.5296	1644.2451	1297.4501	2071.701	965.93123
miR396d-Probable-3p-star	0	0	0	0	0	0
miR396d-Probable-5p-mature	1443.867714	1526.5296	1644.2451	1297.4501	2071.701	965.93123
miR396e-Known-3p-star	232.5805492	302.31723	297.14385	104.39254	128.2787	105.8018
miR396e-Known-5p-mature	21084.34897	19699.186	22880.077	10522.342	14645.151	11195.398
miR396f-Known-3p-star	3.592902408	6.6095973	4.423978	4.26092	4.2759566	0
miR396f-Known-5p-mature	5040.602552	4331.2978	4931.6294	3805.0016	4213.9552	4716.0172
**miR397a-Known-3p-mature**	**4320.584909**	**2335.4868**	**3053.6508**	**820.2271**	**859.46727**	**1295.0924**
miR397a-Known-5p-star	962.4187917	1087.9972	1174.5662	511.3104	491.735	546.64262
miR397b-Known-3p-star	1.437160963	2.5863642	1.8433242	0	2.1379783	0
**miR397b-Known-5p-mature**	**962.1792649**	**1088.2846**	**1174.9348**	**511.3104**	**491.735**	**548.60192**
miR398a-Probable-3p-mature	23.23410224	26.151015	21.013895	10.6523	2.1379783	1.9592926
miR398a-Probable-5p-star	0	0	0	0	0	0
**miR398b-Known-3p-mature**	**11733.70068**	**14555.483**	**11614.048**	**6150.638**	**6518.6958**	**6265.8176**
miR398b-Known-5p-star	0.479053654	0	0	0	0	0
**miR398c-Known-3p-mature**	**11733.70068**	**14555.483**	**11614.048**	**6150.638**	**6518.6958**	**6265.8176**
miR398c-Known-5p-star	0.958107309	1.436869	1.4746593	0	0	0
miR398d-Known-3p-mature	125.7515843	180.18337	118.71008	85.2184	62.00137	97.964628
miR398d-Known-5p-star	2.634795099	2.873738	2.211989	0	0	3.9185851
**miR399a-Known-3p-mature**	**92.69688213**	**103.74194**	**116.49809**	**117.1753**	**96.209022**	**111.67968**
miR399a-Known-5p-star	1.437160963	2.0116166	2.211989	0	2.1379783	0
miR399b-Known-3p-mature	4.551009717	5.7474759	8.1106263	4.26092	4.2759566	7.8371702
miR399b-Known-5p-star	0.239526827	0.8621214	0	0	0	1.9592926
miR399c-Known-3p-mature	9.581073088	18.391923	8.1106263	4.26092	4.2759566	7.8371702
miR399c-Known-5p-star	0	0	0	0	0	0
miR399d-Known-3p-mature	14.37160963	18.391923	22.85722	8.52184	0	15.674341
miR399d-Known-5p-star	0.239526827	0	0.7373297	0	0	0
**miR399e-Known-3p-mature**	**94.13404309**	**102.87982**	**119.07874**	**117.1753**	**96.209022**	**111.67968**
miR399e-Known-5p-star	1.437160963	2.0116166	2.211989	0	2.1379783	0
miR399f-Known-3p-star	0.718580482	0.2873738	0.3686648	0	0	1.9592926
miR399f-Known-5p-mature	0.958107309	0.8621214	0.7373297	0	2.1379783	0
miR399g-Known-3p-mature	9.581073088	18.391923	8.1106263	4.26092	4.2759566	7.8371702
miR399g-Known-5p-star	0	0	0	0	0	0
miR399h-Known-3p-mature	14.37160963	18.391923	22.85722	8.52184	0	15.674341
miR399h-Known-5p-star	0	0.2873738	0	0	0	0
miR403-Known-3p-mature	799.780076	1793.7872	1236.8705	982.14206	902.22683	672.03735
miR403-Known-5p-star	0.239526827	0.8621214	1.4746593	0	0	0
miR408-Known-3p-mature	1128.889937	1187.7159	1215.8566	871.35814	889.39896	1067.8144
miR408-Known-5p-star	589.9545754	901.20422	1090.8792	600.78972	508.83883	517.25324
miR4414-Known-3p-star	0.958107309	0.2873738	2.211989	0	0	0
miR4414-Known-5p-mature	1.197634136	0.8621214	1.4746593	0	0	1.9592926
miR477a-Known-3p-star	1.916214618	4.8853545	3.3179835	0	0	5.8778777
miR477a-Known-5p-mature	34.01280946	55.463143	31.705175	42.6092	47.035522	35.267266
miR477b-Known-3p-star	4.790536544	5.4601021	5.1613076	2.13046	0	3.9185851
miR477b-Known-5p-mature	6.706751162	18.391923	13.640599	0	2.1379783	5.8778777
miR477c-Probable-3p-star	1.67668779	2.2989904	1.8433242	0	0	0
miR477c-Probable-5p-mature	34.01280946	55.463143	32.07384	42.6092	47.035522	35.267266

miR477d-Known-3p-mature	6.706751162	8.0464663	3.3179835	2.13046	10.689891	3.9185851
miR477d-Known-5p-star	6.706751162	18.391923	13.640599	0	2.1379783	5.8778777
miR477e-Known-3p-mature	33.05470215	38.220715	33.5485	12.78276	8.5519131	15.674341
miR477e-Known-5p-star	14.37160963	22.70253	18.064577	2.13046	0	7.8371702
miR482a-Probable-3p-star	0	0	0	0	0	0
miR482a-Probable-5p-mature	1.916214618	2.873738	2.5806538	0	2.1379783	0
miR482b-Known-3p-star	429.2320744	509.80111	707.09915	464.44028	609.32381	384.02134
miR482b-Known-5p-mature	746.1260668	863.84563	649.9561	820.2271	1240.0274	719.06037
**miR482c-Known-3p-mature**	**1041.223118**	**1291.4578**	**1424.5209**	**541.13684**	**696.98092**	**413.41073**
miR482c-Known-5p-star	6.706751162	10.920204	13.271934	2.13046	19.241804	15.674341
miR482d-Known-3p-mature	1175.597668	1525.3801	1626.9179	681.7472	1045.4714	560.35767
miR482d-Known-5p-star	62.99555556	79.027794	122.76539	38.34828	59.863392	27.430096
miR5139-Probable-3p-mature	32.09659485	39.657584	50.507082	17.04368	23.517761	19.592926
miR5139-Probable-5p-star	0	0	0	0	0	0
miR5156-Known-3p-mature	20.59930714	27.013137	17.695912	10.6523	23.517761	17.633633
miR5156-Known-5p-star	0.239526827	0.5747476	0	0	0	0
miR5225a-Known-3p-star	34.73138995	24.714146	32.442505	10.6523	12.82787	1.9592926
miR5225a-Known-5p-mature	30.65943388	39.944958	66.728334	14.91322	12.82787	7.8371702
miR5225b-Known-3p-star	0.718580482	1.7242428	2.9493187	2.13046	0	0
miR5225b-Known-5p-mature	2.395268272	2.5863642	1.8433242	6.39138	2.1379783	0
miR535a-Known-3p-star	11.73681453	12.069699	12.534604	6.39138	6.4139348	5.8778777
miR535a-Known-5p-mature	960.5025771	926.20574	1057.6994	409.04832	615.73774	444.75941
miR535b-Known-3p-star	0.239526827	1.436869	2.211989	0	2.1379783	1.9592926
miR535b-Known-5p-mature	451.2685425	557.79254	374.56347	291.87302	342.07652	248.83016
miR535c-Known-3p-star	12.69492184	18.391923	14.746593	4.26092	12.82787	7.8371702
miR535c-Known-5p-mature	451.2685425	557.79254	374.56347	291.87302	342.07652	248.83016
miR535d-Known-3p-star	8.622965779	10.058083	8.847956	8.52184	6.4139348	1.9592926
miR535d-Known-5p-mature	4761.553798	4155.9998	4269.5074	2690.771	3425.0412	2968.3282
miR7120-Known-3p-mature	134.3745501	171.56216	159.26321	100.13162	162.48635	105.8018
miR7120-Known-5p-star	44.07293621	50.290414	46.451769	34.08736	40.621587	27.430096
miR7121-Known-3p-star	5.269590199	5.1727283	6.2673021	2.13046	4.2759566	1.9592926
miR7121-Known-5p-mature	265.6352514	326.74401	365.71551	187.48048	228.76368	131.2726
miR7122a-Known-3p-star	415.0999916	336.80209	447.92777	308.9167	397.66396	284.09742
miR7122a-Known-5p-mature	462.5263033	733.95267	485.16292	637.00754	498.14894	558.39838
miR7122b-Known-3p-star	415.0999916	336.80209	447.92777	308.9167	397.66396	284.09742
**miR7122b-Known-5p-mature**	**462.5263033**	**733.95267**	**485.16292**	**637.00754**	**498.14894**	**558.39838**
miR7125-Probable-3p-mature	69.70230672	76.154056	44.23978	100.13162	68.415305	125.39472
miR7125-Probable-5p-star	0	0	0	0	0	0
**miR7126-Known-3p-mature**	**9326.935125**	**7905.0784**	**7952.1004**	**3163.7331**	**3472.0767**	**3650.162**
miR7126-Known-5p-star	16.28782425	27.875258	29.861851	10.6523	17.103826	17.633633
miR7127-Probable-3p-star	0	0	0	0	0	0
miR7127-Probable-5p-mature	1.437160963	1.7242428	2.5806538	0	4.2759566	3.9185851
miR7782-Probable-3p-star	0	0	0	0	0	0
miR7782-Probable-5p-mature	6.706751162	6.6095973	8.4792911	4.26092	8.5519131	1.9592926
**miR827-Known-3p-mature**	**136.7698183**	**168.40104**	**159.63187**	**95.8707**	**94.071044**	**80.330995**
miR827-Known-5p-star	0.239526827	1.7242428	0.3686648	0	0	0
**miR858-Probable-3p-mature**	**108.0265991**	**85.637391**	**79.262939**	**176.82818**	**186.00411**	**190.05138**

miR858-Probable-5p-star	0	0	0	0	0	0
miRN10-Novel-3p-star	0	0	0	0	0	0
miRN10-Novel-5p-mature	0	0	0	0	0	0
miRN11-Novel-3p-mature	0.718580482	0.2873738	1.4746593	0	4.2759566	1.9592926
miRN11-Novel-5p-star	0	0	0	0	2.1379783	9.7964628
miRN12-Novel-3p-star	0	0	0	0	0	0
miRN12-Novel-5p-mature	0	0	0	0	0	1.9592926
miRN13-Novel-3p-mature	10.5391804	13.793942	6.635967	6.39138	6.4139348	7.8371702
miRN13-Novel-5p-star	0.479053654	0	0	0	0	0
miRN14-Novel-3p-mature	10.5391804	13.793942	6.635967	6.39138	6.4139348	7.8371702
miRN14-Novel-5p-star	0.239526827	0	0	0	0	0
**miRN15-Novel-3p-mature**	**0.958107309**	**0.5747476**	**0.7373297**	**0**	**2.1379783**	**0**
miRN15-Novel-5p-star	0	0	0	0	0	0
miRN16a-Novel-3p-star	0	0	0	0	0	0
miRN16a-Novel-5p-mature	27.78511196	34.197482	40.553131	17.04368	8.5519131	3.9185851
miRN16b-Novel-3p-star	0	0	0	0	0	0
miRN16b-Novel-5p-mature	27.78511196	34.197482	40.553131	17.04368	8.5519131	3.9185851
miRN16c-Novel-3p-star	0	0	0	0	0	0
miRN16c-Novel-5p-mature	27.78511196	34.197482	40.553131	17.04368	8.5519131	3.9185851
miRN17-Novel-3p-star	0.239526827	0	0.7373297	0	4.2759566	0
**miRN17-Novel-5p-mature**	**38.56381918**	**52.302031**	**65.253675**	**36.21782**	**34.207652**	**52.900899**
miRN18-Novel-3p-star	0.239526827	0	0	0	0	0
miRN18-Novel-5p-mature	0	0	0	0	0	0
miRN19-Novel-3p-star	0.958107309	4.0232331	1.4746593	0	0	0
miRN19-Novel-5p-mature	1.437160963	5.1727283	2.9493187	0	0	0
miRN1a-Novel-3p-star	0.479053654	0.2873738	0	0	0	0
miRN1a-Novel-5p-mature	8.143912125	14.081316	14.377928	2.13046	6.4139348	5.8778777
miRN1b-Novel-3p-star	0.479053654	0.2873738	0	0	0	0
miRN1b-Novel-5p-mature	8.143912125	14.081316	14.377928	2.13046	6.4139348	5.8778777
miRN1c-Novel-3p-star	0.479053654	0.2873738	0	0	0	0
miRN1c-Novel-5p-mature	8.143912125	14.081316	14.377928	2.13046	6.4139348	5.8778777
miRN1d-Novel-3p-star	0.479053654	0.2873738	0	0	0	0
miRN1d-Novel-5p-mature	8.143912125	14.081316	14.377928	2.13046	6.4139348	5.8778777
miRN1e-Novel-3p-star	0.479053654	0.2873738	0	0	0	0
miRN1e-Novel-5p-mature	8.143912125	14.081316	14.377928	2.13046	6.4139348	5.8778777
miRN1f-Novel-3p-star	0.479053654	0.2873738	0	0	0	0
miRN1f-Novel-5p-mature	8.143912125	14.081316	14.377928	2.13046	6.4139348	5.8778777
**miRN20-Novel-3p-mature**	**11.73681453**	**19.828792**	**19.539236**	**12.78276**	**12.82787**	**5.8778777**
miRN20-Novel-5p-star	0	0	0	0	0	0
miRN21-Novel-3p-mature	0	0	0	0	0	0
miRN21-Novel-5p-star	0	0	0	0	0	0
miRN22-Novel-3p-star	0	0	0	0	0	0
miRN22-Novel-5p-mature	0.479053654	0	0	0	0	0
miRN23-Novel-3p-star	0	0	0	0	0	0
miRN23-Novel-5p-mature	0	1.1494952	0	0	0	0

miRN24-Novel-3p-mature	0.239526827	0	0	0	0	0
miRN24-Novel-5p-star	0	0	0	0	0	0
miRN25-Novel-3p-star	0	0.2873738	0	0	0	0
miRN25-Novel-5p-mature	1.197634136	3.7358593	2.211989	0	0	1.9592926
miRN26-Novel-3p-mature	1.67668779	3.1611118	1.4746593	6.39138	2.1379783	3.9185851
miRN26-Novel-5p-star	1.197634136	0.5747476	1.4746593	0	0	1.9592926
miRN27a-Novel-3p-mature	10.5391804	4.3106069	8.1106263	6.39138	0	1.9592926
miRN27a-Novel-5p-star	0	0	0	0	0	0
miRN27b-Novel-3p-mature	10.5391804	4.3106069	8.1106263	6.39138	0	1.9592926
miRN27b-Novel-5p-star	0	0	0	0	0	0
miRN28-Novel-3p-star	0	0	0	0	0	0
**miRN28-Novel-5p-mature**	**53.41448247**	**62.07274**	**77.05095**	**29.82644**	**19.241804**	**17.633633**
miRN29-Novel-3p-star	0.958107309	1.7242428	1.1059945	0	0	1.9592926
miRN29-Novel-5p-mature	3.353375581	4.5979807	4.0553131	0	8.5519131	0
miRN2-Novel-3p-star	0	0	0	0	0	0
miRN2-Novel-5p-mature	0	0	0	0	0	0
miRN30-Novel-3p-star	1.67668779	2.0116166	6.2673021	0	2.1379783	0
miRN30-Novel-5p-mature	10.5391804	10.058083	14.009264	2.13046	2.1379783	0
miRN31-Novel-3p-star	1.197634136	2.5863642	3.3179835	0	0	0
miRN31-Novel-5p-mature	9.341546261	13.219195	9.9539504	10.6523	2.1379783	13.715048
**miRN32-Novel-3p-mature**	**1.437160963**	**1.1494952**	**1.1059945**	**0**	**2.1379783**	**0**
miRN32-Novel-5p-star	0	0	0	0	0	0
**miRN33-Novel-3p-mature**	**461.0891424**	**439.68191**	**528.2967**	**345.13452**	**367.73226**	**323.28327**
miRN33-Novel-5p-star	0	0	0	0	0	0
miRN34-Novel-3p-star	0	0	0	0	0	0
miRN34-Novel-5p-mature	0	0.2873738	0.3686648	0	0	0
miRN35a-Novel-3p-star	0	0	0	0	0	0
miRN35a-Novel-5p-mature	3.832429235	5.4601021	5.1613076	0	8.5519131	3.9185851
miRN35b-Novel-3p-star	0	0	0	0	0	0
miRN35b-Novel-5p-mature	3.832429235	5.4601021	5.1613076	0	8.5519131	3.9185851
miRN36-Novel-3p-mature	0	0	0	0	0	0
miRN36-Novel-5p-star	0	0	0	0	0	0
miRN37-Novel-3p-mature	3.832429235	3.7358593	3.3179835	0	4.2759566	0
miRN37-Novel-5p-star	0	0.5747476	0	0	0	0
miRN38-Novel-3p-mature	7.904385298	11.207578	7.7419615	2.13046	4.2759566	3.9185851
miRN38-Novel-5p-star	0	0	0	0	0	0
miRN39-Novel-3p-mature	3.113848754	4.0232331	3.6866483	0	0	0
miRN39-Novel-5p-star	2.634795099	1.7242428	1.1059945	2.13046	2.1379783	3.9185851
miRN3a-Novel-3p-star	1.197634136	0.5747476	0.3686648	4.26092	0	0
miRN3a-Novel-5p-mature	0.718580482	0.5747476	2.5806538	0	0	0
miRN3b-Novel-3p-star	1.197634136	0.5747476	0.3686648	4.26092	0	0
miRN3b-Novel-5p-mature	0.718580482	0.5747476	2.5806538	0	0	0
miRN3c-Novel-3p-star	1.197634136	0.5747476	0.3686648	4.26092	0	0
miRN3c-Novel-5p-mature	0.718580482	0.5747476	2.5806538	0	0	0
miRN3d-Novel-3p-star	1.197634136	0.5747476	0.3686648	4.26092	0	0
miRN3d-Novel-5p-mature	0.718580482	0.5747476	2.5806538	0	0	0
miRN4-Novel-3p-mature	0.958107309	1.436869	0.3686648	0	0	0

miRN4-Novel-5p-star	0.479053654	0	0.3686648	0	0	0
miRN5-Novel-3p-mature	1.437160963	2.873738	2.5806538	2.13046	2.1379783	0
miRN5-Novel-5p-star	0	0	0	0	0	0
miRN6-Novel-3p-mature	0.479053654	1.7242428	1.1059945	0	0	1.9592926
miRN6-Novel-5p-star	1.437160963	0.8621214	1.8433242	4.26092	0	0
**miRN7-Novel-3p-mature**	**46.70773131**	**60.635871**	**50.507082**	**31.9569**	**55.587435**	**48.982314**
miRN7-Novel-5p-star	0.958107309	2.2989904	1.4746593	2.13046	0	0
miRN8-Novel-3p-star	0.239526827	0	0	0	0	0
miRN8-Novel-5p-mature	1.67668779	1.436869	1.1059945	0	2.1379783	5.8778777
miRN9-Novel-3p-star	0.239526827	0.5747476	0.7373297	0	0	0
miRN9-Novel-5p-mature	0	0	0	0	0	0

115_S_CK_r	115_S_CK_r	115_S_CK_r	115_S_Pu_i	115_S_Pu_i	115_S_Pu_i	132_S_CK_r	132_S_CK_r	132_S_CK_r	132_S_Pu_i
4.6973666	6.3580434	7.4705927	2.4770385	6.6481007	5.6165282	7.7613842	11.930653	6.4043813	3.2106357
0	0	0	0	0	0	0	0	0	0
680.03414	561.02164	532.68574	376.50985	409.96621	514.84842	956.86779	1292.744	1112.0568	735.23557
18.42813	30.276397	25.010245	19.816308	28.808437	16.849585	37.03289	42.719436	33.046607	11.237225
0	0	0	0	0	0	0	0	0	0
208.12947	267.03782	271.53981	138.71415	157.33838	161.00714	170.5287	630.40033	278.20632	261.66681
0	0	0	0	0	0	0	0	0	0
1.0840077	0.302764	0.6496168	0	0	0	0.4435077	0.7697196	1.024701	0
0	0.302764	0	0	0	0	0	0	0	0
1.0840077	0.302764	0.6496168	0	0	0	0.4435077	0.7697196	1.024701	0
16.621451	30.276397	22.411778	24.770385	22.160336	22.466113	30.823783	16.164111	27.410752	9.631907
0.3613359	0	0	0	0	0	0.2217538	0.3848598	0.5123505	0
6.1427101	9.3856831	9.0946346	4.9540769	6.6481007	5.6165282	4.8785843	7.6971957	2.049402	0
0	0	0	0	0	0	0	0	0	0
2461.7814	3946.2256	3056.122	1347.5089	1311.8919	1533.3122	27.719229	23.476447	24.592824	35.316992
5.4200383	3.3304037	5.8465508	4.9540769	2.2160336	5.6165282	0	0	0	1.6053178
21.318817	45.71736	28.258329	4.9540769	6.6481007	5.6165282	23.284153	17.70355	25.36135	3.2106357
0	0	0	0	0	0	0	0	0.2561753	0
0	0	0	0	0	0	0	0	0	0
6.1427101	11.807795	4.2225089	0	0	5.6165282	5.1003382	5.388037	6.6605565	0
24632.99	41932.507	32597.119	15072.779	13856.858	17766.951	24053.195	20284.42	22658.189	5864.2261
14.814771	16.954782	17.864461	24.770385	19.944302	20.593937	27.719229	30.019063	17.676092	8.0265892
1175.0643	1696.9921	1242.3921	688.6167	576.16873	748.87042	886.35007	718.53322	824.37196	256.85085
0	0	0	0	0	0	0	0	0	0
5.0587024	4.5414596	4.2225089	2.4770385	0	1.8721761	9.5354149	10.391214	8.7099586	0
0	0	0	0	0	0	0	0	0	0
0.7226718	0.9082919	1.2992335	0	2.2160336	0	1.5522768	0	0.5123505	0
11.562748	12.413323	9.419443	0	0	3.7443521	8.8701534	10.391214	12.040237	1.6053178
31.797558	26.037702	44.498748	14.862231	35.456537	29.954817	41.024459	59.653267	41.756566	30.501039
0	0	0	0	0	0	0	0	0	0
97.199354	70.241241	57.166275	148.62231	110.80168	117.94709	160.99328	196.27849	126.55057	75.449938
1489.0652	891.03437	970.20263	1315.3074	1021.5915	1368.5607	667.25729	890.18068	584.8481	223.13918
0	0.302764	0	2.4770385	0	1.8721761	0.2217538	0.7697196	0	1.6053178
4.3360307	3.0276397	5.5217425	7.4311154	0	9.3608803	5.7655997	9.6214946	5.3796803	8.0265892
16.260115	22.10177	25.335054	52.017808	50.968772	31.826993	21.288368	22.706727	19.725494	22.47445
1532.0642	1378.1816	1236.8703	1362.3712	1413.8294	1263.7188	1511.0306	1763.4275	1696.9049	431.8305
62.872445	41.478664	69.184185	79.265231	84.209276	58.037458	123.07338	144.70728	103.75098	64.212714
1571.8111	1532.5912	1402.5226	1513.4705	1728.5062	1224.4031	2511.3622	2381.5124	2354.2506	741.65684
97.199354	70.241241	57.166275	148.62231	110.80168	117.94709	160.99328	196.27849	126.55057	75.449938
1506.4093	899.209	980.5965	1335.1237	1039.3197	1387.2825	669.47482	892.10498	586.12898	224.7445
6119.2233	3962.8776	7926.6237	4109.4068	3290.8099	5515.4307	2897.6573	2867.2054	2817.1592	756.1047
951.03606	535.58947	664.88275	802.56047	660.37801	969.7872	324.64761	377.54745	283.32983	94.713753
27.461528	25.12941	42.549898	14.862231	33.240504	29.954817	39.028675	58.883547	39.963339	27.290403
951.03606	535.58947	664.88275	802.56047	660.37801	969.7872	324.64761	377.54745	283.32983	94.713753
16.260115	22.10177	25.335054	52.017808	50.968772	31.826993	21.288368	22.706727	19.725494	22.47445
1532.0642	1378.1816	1236.8703	1362.3712	1413.8294	1263.7188	1511.0306	1763.4275	1696.9049	431.8305

62.872445	41.1759	69.184185	79.265231	84.209276	58.037458	122.62987	143.5527	104.00715	64.212714
119.24084	140.78525	121.15353	99.081539	106.36961	71.14269	173.18974	176.26578	203.14697	33.711675
1.8066794	0.9082919	3.2480838	7.4311154	6.6481007	7.4887042	5.1003382	10.391214	6.4043813	6.4212714
4.3360307	3.0276397	5.5217425	7.4311154	0	9.3608803	5.7655997	9.6214946	5.3796803	8.0265892
0	0	0	0	0	0	0	0	0	0
16.260115	13.927143	13.317144	17.339269	13.296201	20.593937	7.3178765	10.776074	5.3796803	0
62.872445	41.478664	69.184185	79.265231	84.209276	58.037458	123.07338	144.70728	103.75098	64.212714
1571.8111	1532.5912	1402.5226	1513.4705	1728.5062	1224.4031	2511.3622	2381.5124	2354.2506	741.65684
97.199354	70.241241	57.166275	148.62231	110.80168	117.94709	160.99328	196.27849	126.55057	75.449938
1489.0652	891.03437	970.20263	1315.3074	1021.5915	1368.5607	667.25729	890.18068	584.8481	223.13918
367.83994	336.37077	411.85703	445.86693	372.29364	539.1867	398.49164	639.63696	416.28478	219.92854
951.03606	535.58947	664.88275	802.56047	660.37801	969.7872	324.64761	377.54745	283.32983	94.713753
365.67192	332.7376	411.53222	440.91285	365.64554	537.31453	396.93936	638.48238	414.49156	216.71791
951.03606	535.58947	664.88275	802.56047	660.37801	969.7872	324.64761	377.54745	283.32983	94.713753
0	0	0	0	0	0	0.6652615	0	0	0
1489.0652	891.03437	970.20263	1315.3074	1021.5915	1368.5607	667.25729	890.56554	584.8481	223.13918
0	0	0	0	0	0	0.6652615	0	0	0
1489.0652	891.03437	970.20263	1315.3074	1021.5915	1368.5607	667.25729	890.56554	584.8481	223.13918
7.5880537	5.1469875	5.5217425	17.339269	6.6481007	3.7443521	6.652615	5.388037	4.6111545	4.8159535
1489.0652	891.03437	970.20263	1315.3074	1021.5915	1368.5607	667.25729	890.56554	584.8481	223.13918
10.840077	6.6608074	10.393868	12.385192	8.8641343	5.6165282	17.740307	15.009532	12.296412	12.842543
951.75873	535.58947	665.53237	802.56047	662.59404	969.7872	324.64761	377.54745	283.586	94.713753
14.0921	13.321615	12.342718	14.862231	11.080168	20.593937	6.4308612	9.2366348	4.6111545	0
0	0	0	0	0	0	0	0	0	0
28477.965	30711.772	26875.295	23581.406	20547.063	27041.711	20952.854	32796.211	21895.555	5796.8027
254.01913	97.792763	63.987251	170.91565	135.17805	151.64626	369.44189	448.74651	322.52464	43.343582
76.603209	65.699782	79.902862	19.816308	8.8641343	13.105232	65.860889	61.577566	47.648597	24.079768
0	0	0	0	0	0	0	0	0	0
28478.327	30712.075	26875.295	23581.406	20547.063	27041.711	20952.854	32796.211	21895.555	5796.8027
254.01913	97.792763	63.987251	170.91565	135.17805	151.64626	369.22013	448.74651	322.52464	43.343582
8.6720613	13.624379	9.0946346	9.9081539	4.4320672	5.6165282	16.409784	23.091587	19.469319	1.6053178
266.30455	280.96497	285.50657	473.11435	489.74342	462.42749	279.63158	342.14035	312.27763	438.25177
21.680153	25.432174	22.411778	14.862231	13.296201	13.105232	29.271506	28.864484	29.972504	1.6053178
267.74989	283.38708	287.78022	473.11435	491.95945	466.17184	281.40562	343.67979	312.53381	439.85709
18.789466	24.523882	16.240419	14.862231	4.4320672	14.977408	17.962061	21.937008	24.848999	6.4212714
266.30455	280.96497	285.50657	473.11435	489.74342	462.42749	279.63158	342.14035	312.27763	438.25177
11.924084	4.5414596	1.9488503	2.4770385	4.4320672	1.8721761	24.171168	22.706727	10.24701	1.6053178
267.74989	283.38708	287.78022	473.11435	491.95945	466.17184	281.40562	343.67979	312.53381	439.85709
1353.9256	1110.841	1234.9215	1233.5652	1116.8809	1289.9293	1168.421	1617.5657	1199.6687	622.86332
127.55157	144.41841	124.40161	108.98969	106.36961	114.20274	108.65938	143.93756	104.5195	113.97757
1353.9256	1110.841	1234.9215	1233.5652	1116.8809	1289.9293	1168.421	1617.5657	1199.6687	622.86332
127.55157	144.41841	124.40161	108.98969	106.36961	114.20274	108.65938	143.93756	104.5195	113.97757
1.0840077	0.302764	0.6496168	0	0	0	2.4392922	3.0788783	3.8426288	1.6053178
573.80139	501.98267	330.00531	282.38239	274.78816	374.43521	371.65943	376.00801	540.01743	110.76693
432.8804	646.09832	407.30971	260.08904	283.6523	299.54817	301.36346	366.38652	442.92701	268.08808
573.80139	502.28543	330.33012	284.85942	274.78816	374.43521	371.65943	376.77773	539.50508	110.76693
0	0	0	0	0	0	0	0.3848598	0.2561753	0

573.80139	501.98267	330.00531	282.38239	274.78816	374.43521	371.65943	376.00801	540.01743	110.76693
0.7226718	0.302764	0.9744251	0	0	1.8721761	0.6652615	0.3848598	1.7932268	0
1.4453436	3.6331677	0.9744251	0	4.4320672	1.8721761	2.2175383	1.1545794	1.2808763	0
0	0	1.2992335	0	0	0	0	0	0	0
0.3613359	0.302764	0.3248084	0	0	0	0.2217538	0.3848598	0	0
432.8804	646.09832	407.30971	260.08904	283.6523	299.54817	301.36346	366.38652	442.92701	268.08808
573.80139	502.28543	330.33012	284.85942	274.78816	374.43521	371.65943	376.77773	539.50508	110.76693
0	0	0	0	0	0	0	0	0	0
573.80139	501.98267	330.00531	282.38239	274.78816	374.43521	371.65943	376.00801	540.01743	110.76693
0.3613359	0	0	0	0	0	0	0	0	0
17.344123	7.5690993	6.1713592	7.4311154	6.6481007	0	10.865938	14.624672	9.7346596	3.2106357
561417.33	611100.31	606823.58	342599.19	304124.02	417547.68	668336.34	608427.14	643611.63	316600.78
3441.7243	1483.5435	1067.3203	1704.2025	1480.3104	2257.8443	3462.6861	3886.699	3542.9037	520.12298
**562202.51**	**611707.36**	**607280.91**	**343010.38**	**304540.63**	**418120.57**	**669096.96**	**609044.84**	**644285.11**	**316976.43**
10.478741	4.2386956	4.2225089	4.9540769	17.728269	9.3608803	11.974707	15.394391	13.064938	0
562024.73	611488.46	607072.06	342824.6	304376.64	417899.65	668859.46	608988.65	644057.89	316762.92
101.17405	57.222391	39.951431	47.063731	39.888604	50.548754	78.944365	101.98784	86.84341	12.842543
561417.33	611100.31	606823.58	342599.19	304124.02	417547.68	668336.34	608427.14	643611.63	316600.78
2993.3065	1069.0596	648.31753	1466.4068	1265.3552	1930.2135	2892.1135	3257.8381	2772.0724	422.19859
561417.33	611100.31	606823.58	342599.19	304124.02	417547.68	668336.34	608427.14	643611.63	316600.78
3447.1444	1487.7822	1073.4917	1706.6795	1486.9585	2263.4609	3471.5563	3892.4719	3553.1507	521.7283
32741.368	29544.011	24259.938	18171.554	17367.055	19474.375	27174.824	36975.404	28355.014	18404.969
2.8906871	1.5138199	1.6240419	2.4770385	0	0	2.2175383	3.0788783	2.8179278	0
**562202.51**	**611707.36**	**607280.91**	**343010.38**	**304540.63**	**418120.57**	**669096.96**	**609044.84**	**644285.11**	**316976.43**
3.9746948	1.8165838	0.6496168	2.4770385	0	1.8721761	8.4266457	6.5426164	9.2223091	1.6053178
562189.5	611677.69	607274.42	343012.86	304491.88	418075.64	669105.38	609025.6	644289.47	317008.54
0.3613359	0	0.3248084	0	0	0	0.4435077	0	0.2561753	0
562189.5	611677.69	607274.42	343012.86	304491.88	418075.64	669105.38	609025.6	644289.47	317008.54
85.27527	59.038974	48.721257	22.293346	33.240504	48.676577	81.161903	86.208592	81.976081	12.842543
562014.25	611459.39	607067.51	342827.08	304327.89	417854.72	668870.32	608970.57	644063.78	316795.03
0.3613359	0	0.3248084	0	0	0	0.4435077	0	0.2561753	0
3.9746948	5.4497515	6.1713592	0	6.6481007	1.8721761	28.827998	26.555325	24.080474	1.6053178
114.18214	150.77646	164.67785	250.18089	237.11559	235.89418	244.59448	204.36055	278.97485	200.66473
0	0	0	0	0	0	0.2217538	0	0	0
107.6781	137.15208	150.38628	225.4105	226.03543	226.5333	224.85839	192.04503	258.22465	191.03282
2.1680153	0.6055279	2.2736587	0	0	0	1.9957845	3.0788783	2.3055773	0
114.18214	151.07922	164.67785	247.70385	237.11559	235.89418	244.37272	204.36055	277.69397	200.66473
3.9746948	5.4497515	6.1713592	0	6.6481007	1.8721761	28.827998	26.555325	24.080474	1.6053178
114.18214	150.77646	164.67785	250.18089	237.11559	235.89418	244.59448	204.36055	278.97485	200.66473
6.1427101	0.302764	0	0	0	0	5.5438458	5.0031772	7.172907	0
107.6781	137.15208	150.38628	225.4105	226.03543	226.5333	224.85839	192.04503	258.22465	191.03282
3.9746948	5.4497515	6.1713592	0	6.6481007	1.8721761	28.827998	26.555325	24.080474	1.6053178
114.18214	151.07922	164.67785	247.70385	237.11559	235.89418	244.37272	204.36055	277.69397	200.66473
2.8906871	3.3304037	1.6240419	2.4770385	2.2160336	3.7443521	6.8743688	3.0788783	4.098804	0
37.217597	47.23118	60.739167	121.37489	132.96201	106.71404	71.848242	58.113828	70.192019	83.476528
2.8906871	3.3304037	1.6240419	2.4770385	2.2160336	3.7443521	6.8743688	3.0788783	4.098804	0
37.217597	47.23118	60.739167	121.37489	132.96201	106.71404	71.848242	58.113828	70.192019	83.476528

2081.6561	1703.3501	1083.2359	1865.21	1710.7779	2094.965	3431.8623	4296.1898	4053.7172	878.10886
7083.9901	7592.4121	6254.835	4723.7124	4199.3836	5272.0478	6439.5096	7032.5429	7205.4413	3759.6544
2081.6561	1703.3501	1083.2359	1865.21	1710.7779	2094.965	3431.8623	4296.1898	4053.7172	878.10886
7083.9901	7592.4121	6254.835	4723.7124	4199.3836	5272.0478	6439.5096	7032.5429	7205.4413	3759.6544
0	0	0	0	0	0	0.2217538	0	0	0
13.369428	10.293975	14.291569	7.4311154	8.8641343	9.3608803	49.451105	35.4071	36.120711	11.237225
9.3947331	8.1746272	4.2225089	0	2.2160336	7.4887042	14.192245	13.085233	17.932268	0
12.646756	8.1746272	12.667527	7.4311154	8.8641343	9.3608803	48.785843	35.02224	35.352185	11.237225
0.7226718	0.6055279	0.3248084	0	2.2160336	0	4.6568305	0.7697196	2.5617525	0
0	0.9082919	0.3248084	0	0	1.8721761	1.1087692	2.3091587	0.7685258	0
2.8906871	3.3304037	0.9744251	4.9540769	0	11.233056	5.7655997	8.0820555	7.6852576	8.0265892
533.33177	550.72766	490.78546	230.36458	210.52319	273.3377	1451.3788	928.66666	1419.7232	166.95306
0.3613359	0	0	0	0	0	14.413999	10.776074	17.163742	1.6053178
13.008092	10.293975	14.291569	7.4311154	8.8641343	9.3608803	49.451105	35.4071	36.120711	11.237225
0	0	0	0	0	0	0	0	0	0
13.730764	10.899503	9.0946346	12.385192	11.080168	3.7443521	7.7613842	5.388037	3.074103	4.8159535
2.5293512	1.5138199	0.9744251	0	0	0	6.2091073	1.1545794	7.172907	0
13.008092	10.293975	14.291569	7.4311154	8.8641343	9.3608803	49.451105	35.4071	36.120711	11.237225
0.3613359	0.6055279	0.6496168	4.9540769	0	1.8721761	3.1045537	2.6940185	3.5864535	1.6053178
12.646756	8.1746272	12.667527	7.4311154	8.8641343	9.3608803	48.785843	35.02224	35.352185	11.237225
2.5293512	1.5138199	0.9744251	0	0	0	6.2091073	1.1545794	7.172907	0
13.008092	10.293975	14.291569	7.4311154	8.8641343	9.3608803	49.451105	35.4071	36.120711	11.237225
7.2267178	7.2663353	3.8977006	4.9540769	6.6481007	3.7443521	2.4392922	3.0788783	2.8179278	3.2106357
13.730764	10.899503	9.0946346	12.385192	11.080168	3.7443521	7.7613842	5.388037	3.8426288	4.8159535
1.0840077	0.302764	0	0	0	0	0.2217538	0.7697196	1.7932268	0
13.369428	10.293975	14.291569	7.4311154	8.8641343	9.3608803	49.451105	35.4071	36.120711	11.237225
0.3613359	0	0	0	0	0	0	0	0	0
13.730764	10.899503	9.0946346	12.385192	11.080168	3.7443521	7.7613842	5.388037	3.074103	4.8159535
214.63352	263.10189	260.49632	123.85192	97.505477	125.4358	310.01186	229.37643	240.03621	56.186124
0.7226718	1.8165838	1.9488503	4.9540769	6.6481007	7.4887042	1.9957845	2.3091587	1.5370515	4.8159535
254.38047	190.43854	165.97708	203.11715	128.52995	198.45066	227.96294	184.34784	214.93104	128.42543
27.822863	20.890714	19.163694	12.385192	11.080168	14.977408	25.945199	31.558502	24.080474	4.8159535
58.175078	61.461086	62.363209	34.678539	28.808437	31.826993	88.701534	69.274761	105.5442	11.237225
30.713551	42.084192	39.626622	9.9081539	24.376369	13.105232	36.811136	27.709905	44.318319	4.8159535
131.8876	115.35307	111.73408	99.081539	104.15358	114.20274	153.2319	189.73587	152.68045	93.108435
0	0	0	0	0	0	0	0	0.2561753	0
58.175078	60.552794	60.08955	34.678539	28.808437	31.826993	88.47978	68.120182	103.23863	11.237225
12.28542	15.138199	10.718677	0	2.2160336	7.4887042	30.823783	28.094764	37.657762	6.4212714
254.38047	190.43854	165.97708	203.11715	128.52995	198.45066	227.96294	184.34784	214.93104	128.42543
27.822863	20.890714	19.163694	12.385192	11.080168	14.977408	25.945199	31.558502	24.080474	4.8159535
132.24894	115.65584	111.73408	99.081539	104.15358	114.20274	153.45365	190.12073	152.68045	94.713753
0	0	0	0	0	0	0.4435077	0	0	0
2.1680153	1.2110559	1.9488503	2.4770385	0	0	1.9957845	2.6940185	1.5370515	1.6053178
0	0	0	0	0	0	0	0	0	0
0.3613359	0	0.3248084	0	0	0	0.4435077	1.1545794	0.2561753	0
2.5293512	4.5414596	2.9232754	0	0	0	2.4392922	0	3.5864535	0
2.1680153	1.2110559	1.9488503	2.4770385	0	0	1.9957845	2.6940185	1.5370515	1.6053178

1.0840077	0	0	0	0	1.8721761	0	1.1545794	0.5123505	0
0.3613359	0	0.3248084	0	0	0	0.4435077	1.1545794	0.2561753	0
0	0	0	0	0	0	0	0	0	0
0.3613359	0	0.3248084	0	0	0	0.4435077	1.1545794	0.2561753	0
0	0	0	2.4770385	0	0	0	0	0	0
2.1680153	1.2110559	1.9488503	2.4770385	0	0	1.9957845	2.6940185	1.5370515	1.6053178
0	0	0	0	0	0	0	0	0	0
0.3613359	0	0.3248084	0	0	0	0.4435077	1.1545794	0.2561753	0
0	0	0	0	0	0	0	0	0	0
6.504046	8.7801552	6.4961676	4.9540769	2.2160336	5.6165282	7.983138	3.8485979	8.7099586	1.6053178
10.840077	16.652018	20.787736	4.9540769	15.512235	7.4887042	7.0961227	5.0031772	7.6852576	0
10.840077	16.652018	20.787736	4.9540769	15.512235	7.4887042	7.0961227	5.0031772	7.6852576	0
0	0	0	0	0	0	0	0	0	0
0	0	0	0	0	0	0	0	0.2561753	0
10.840077	16.652018	20.787736	4.9540769	15.512235	7.4887042	7.0961227	5.0031772	7.6852576	0
3519.7729	3788.7883	3660.5904	2910.5202	2727.9373	3212.6541	3417.0048	3909.0208	3589.0153	2120.6249
16.260115	10.293975	12.342718	7.4311154	8.8641343	14.977408	35.25886	50.031772	47.648597	4.8159535
3519.7729	3788.7883	3660.5904	2910.5202	2727.9373	3212.6541	3417.0048	3909.0208	3589.0153	2120.6249
23.125497	14.229907	9.0946346	24.770385	28.808437	13.105232	27.497475	46.952894	27.410752	3.2106357
44222.815	44592.289	39814.037	29075.478	28185.731	30098.974	40150.527	35432.501	36483.455	12895.518
79.855232	48.744999	32.480838	52.017808	57.616873	31.826993	109.76815	120.84597	110.66771	17.658496
682.56349	713.91745	673.00296	435.95877	425.47845	486.76577	631.99843	569.97734	511.3258	146.08392
9.0333972	9.9912111	7.7954011	12.385192	6.6481007	5.6165282	14.192245	14.239812	11.015536	9.631907
104.06474	138.6659	114.98217	96.6045	66.481007	73.014866	127.2867	122.00055	113.74181	25.685085
0	0	0	0	0	0	0	0	0	0
1202.1645	1181.385	1196.2693	725.77227	706.91471	778.82524	1230.2903	1001.4052	937.60142	229.56045
2.1680153	0	1.2992335	0	0	1.8721761	3.5480613	3.0788783	2.5617525	0
1194.5764	1171.6966	1190.0979	718.34116	700.26661	775.08089	1220.5331	988.31993	929.65999	226.34982
1.4453436	2.4221118	1.2992335	2.4770385	2.2160336	3.7443521	2.2175383	6.5426164	4.3549793	0
44282.075	44659.805	39884.845	29112.633	28230.052	30153.268	40207.296	35482.533	36530.847	12897.124
16.621451	15.440963	14.616377	7.4311154	4.4320672	13.105232	12.861722	17.31869	14.601989	1.6053178
682.56349	713.91745	673.00296	435.95877	425.47845	486.76577	631.99843	569.59248	511.3258	146.08392
4.3360307	4.5414596	6.1713592	0	4.4320672	0	3.3263075	5.388037	4.3549793	1.6053178
1202.1645	1181.385	1196.2693	725.77227	706.91471	778.82524	1230.2903	1001.4052	937.60142	229.56045
2.1680153	0	1.6240419	0	0	1.8721761	3.5480613	3.0788783	2.5617525	0
0	0	0	0	0	0	0	0	0	0
617.52303	647.9149	625.25613	423.57358	394.45398	458.68313	594.07852	502.62688	460.34693	122.00416
1194.5764	1171.6966	1190.0979	718.34116	700.26661	775.08089	1220.5331	988.31993	929.65999	226.34982
4.3360307	4.5414596	6.1713592	0	4.4320672	0	3.3263075	5.388037	4.3549793	1.6053178
617.52303	647.9149	625.25613	423.57358	394.45398	458.68313	594.07852	502.62688	460.34693	122.00416
0	0	0	0	0	0	0	0	0	0
5.0587024	2.7248757	6.1713592	4.9540769	2.2160336	3.7443521	15.522768	29.249344	7.4290823	8.0265892
50.94836	24.221118	31.831221	44.586693	28.808437	43.060049	56.990735	92.366348	29.972504	20.869132
11.924084	7.2663353	7.7954011	9.9081539	2.2160336	20.593937	13.30523	21.167288	6.6605565	6.4212714
86.720613	49.956055	73.731502	54.494846	53.184806	112.33056	77.613842	106.60616	48.160947	30.501039
3.6133589	1.5138199	1.2992335	9.9081539	4.4320672	1.8721761	2.4392922	3.8485979	4.3549793	4.8159535
86.720613	49.956055	73.731502	54.494846	53.184806	112.33056	77.613842	106.60616	48.160947	30.501039

0.3613359	0	0.9744251	0	0	1.8721761	0.2217538	0	0	1.6053178
86.720613	49.956055	73.731502	54.494846	53.184806	112.33056	77.613842	106.60616	48.160947	30.501039
16.982787	42.992484	34.429688	17.339269	31.02447	13.105232	19.07083	20.397569	19.469319	6.4212714
385.90673	597.95884	710.03112	267.52016	259.27593	275.20988	572.34665	461.83174	637.10785	184.61155
5.7813742	9.9912111	12.667527	14.862231	17.728269	7.4887042	8.6483995	9.6214946	8.4537833	4.8159535
386.6294	598.86714	710.35593	267.52016	259.27593	275.20988	573.67717	463.37118	639.6696	184.61155
16.982787	42.992484	34.429688	17.339269	31.02447	13.105232	19.07083	20.397569	19.469319	6.4212714
385.90673	598.86714	710.03112	267.52016	259.27593	277.08206	572.34665	461.83174	637.87638	184.61155
16.982787	42.992484	34.429688	17.339269	31.02447	13.105232	19.07083	20.397569	19.469319	6.4212714
386.6294	598.86714	710.35593	267.52016	259.27593	275.20988	573.67717	463.37118	639.6696	184.61155
0.7226718	0	0	0	0	0	0.4435077	1.9242989	2.3055773	0
22.402825	9.0829192	15.265994	12.385192	11.080168	13.105232	46.568305	43.104296	30.22868	12.842543
9.756069	8.7801552	11.043485	4.9540769	0	3.7443521	5.5438458	5.7728968	2.5617525	6.4212714
52.032368	25.432174	25.335054	29.724462	26.592403	35.571345	29.936768	29.249344	27.666927	16.053178
11.201413	4.5414596	4.8721257	2.4770385	8.8641343	14.977408	6.652615	9.6214946	5.3796803	6.4212714
**238.12035**	**112.6282**	**190.66252**	**200.64012**	**161.77045**	**235.89418**	**190.93005**	**306.73325**	**203.40315**	**78.660574**
9.756069	8.7801552	11.043485	4.9540769	0	3.7443521	5.5438458	5.7728968	2.5617525	6.4212714
52.032368	25.432174	25.335054	29.724462	26.592403	35.571345	29.936768	29.249344	27.666927	16.053178
11.201413	4.5414596	4.8721257	2.4770385	8.8641343	14.977408	6.652615	9.6214946	5.3796803	6.4212714
**238.12035**	**112.6282**	**190.66252**	**200.64012**	**161.77045**	**235.89418**	**190.93005**	**306.73325**	**203.40315**	**78.660574**
87.804621	51.167111	86.074221	29.724462	31.02447	29.954817	33.484829	21.937008	37.657762	6.4212714
3.252023	0.6055279	3.8977006	2.4770385	2.2160336	1.8721761	0.4435077	0.3848598	0.2561753	0
4.3360307	1.2110559	6.820976	4.9540769	2.2160336	0	2.4392922	3.0788783	2.8179278	1.6053178
0	0	0.3248084	0	0	0	0.2217538	1.1545794	0.2561753	0
87.804621	51.167111	86.074221	29.724462	31.02447	29.954817	33.484829	21.937008	37.657762	6.4212714
47.696337	53.589223	24.685437	7.4311154	4.4320672	13.105232	19.07083	12.315513	23.568123	8.0265892
1.4453436	0	0	0	0	1.8721761	0.2217538	0	0.7685258	0
47.696337	53.589223	24.685437	7.4311154	4.4320672	13.105232	19.07083	12.315513	23.568123	8.0265892
86.720613	49.350527	85.099796	29.724462	31.02447	29.954817	33.484829	21.167288	37.145412	6.4212714
47.696337	53.589223	24.685437	7.4311154	4.4320672	13.105232	19.07083	12.315513	23.568123	8.0265892
87.804621	51.167111	86.074221	29.724462	31.02447	29.954817	33.484829	21.937008	37.657762	6.4212714
1.4453436	0.302764	0.3248084	0	0	0	0.4435077	0.7697196	0	0
87.081949	49.350527	84.774987	29.724462	31.02447	29.954817	33.484829	21.167288	37.145412	6.4212714
0	0	0	0	0	0	0	0	0	0
5.4200383	1.2110559	8.7698263	4.9540769	2.2160336	0	2.661046	3.0788783	3.8426288	1.6053178
0	0	0	0	0	0	0	0	0	0
87.081949	49.350527	84.774987	29.724462	31.02447	29.954817	33.484829	21.167288	37.145412	6.4212714
47.696337	53.589223	24.685437	7.4311154	4.4320672	13.105232	19.07083	12.315513	23.568123	8.0265892
87.804621	51.167111	86.074221	29.724462	31.02447	29.954817	33.484829	21.937008	37.657762	6.4212714
47.696337	53.589223	24.685437	7.4311154	4.4320672	13.105232	19.07083	12.315513	23.568123	8.0265892
87.804621	51.167111	86.074221	29.724462	31.02447	29.954817	33.484829	21.937008	37.657762	6.4212714
0.7226718	1.2110559	0.6496168	0	0	0	0	0	1.024701	0
456.36723	182.56667	112.3837	426.05062	363.42951	421.23961	519.56923	775.49247	559.48675	86.687163
7780.6457	8135.2679	8688.949	7572.3066	6524.0029	8263.7851	5155.7766	7984.3011	5371.2265	4904.246
3.9746948	3.6331677	5.8465508	7.4311154	4.4320672	1.8721761	3.5480613	2.3091587	3.5864535	0
5265.7479	6453.4141	6826.4977	5162.1482	4584.9735	5517.3028	3868.9391	3859.3739	3659.7196	3729.1533
0.7226718	0	0	0	0	0	0	0	0	0

5481.1041	2908.9562	3444.2681	3703.1725	4124.0385	2628.5352	2660.159	4049.4947	3761.9336	1292.2809
0.7226718	0	0	0	0	0	0	0	0	0
5481.1041	2908.9562	3444.2681	3703.1725	4124.0385	2628.5352	2660.159	4049.4947	3761.9336	1292.2809
456.36723	182.56667	112.3837	426.05062	363.42951	421.23961	519.56923	775.49247	559.48675	86.687163
19782.417	21318.217	23136.101	16561.479	13418.083	19680.315	16328.178	23588.056	16882.718	10147.214
3.9746948	3.6331677	5.8465508	7.4311154	4.4320672	1.8721761	3.5480613	2.3091587	3.5864535	0
5265.7479	6453.4141	6826.4977	5162.1482	4584.9735	5517.3028	3868.9391	3859.3739	3659.7196	3729.1533
**1692.8586**	**2079.9885**	**2294.1216**	**894.21089**	**828.79656**	**971.65937**	**2012.8595**	**1615.6414**	**2159.3012**	**640.52182**
865.76079	945.53188	1099.4764	604.39739	593.897	608.45722	978.37791	1490.9468	1115.6432	300.19444
3.6133589	6.3580434	3.5728922	4.9540769	6.6481007	0	4.2133228	43.874016	10.759361	9.631907
**863.95411**	**945.83465**	**1100.4508**	**604.39739**	**593.897**	**608.45722**	**977.93441**	**1491.3317**	**1115.1309**	**300.19444**
60.704429	65.397018	22.08697	2.4770385	4.4320672	5.6165282	40.802705	48.492333	54.821504	16.053178
0	0	0	0	0	0	0	0	0	0
**12287.588**	**16961.746**	**11594.36**	**5687.2803**	**5930.1059**	**5509.8141**	**9449.3744**	**23780.871**	**13117.198**	**7923.8489**
0	0.6055279	0.3248084	0	0	0	1.1087692	0.3848598	0.5123505	0
**12287.588**	**16961.746**	**11594.36**	**5687.2803**	**5930.1059**	**5509.8141**	**9449.3744**	**23780.871**	**13117.198**	**7923.8489**
0.3613359	0.6055279	0.6496168	0	0	0	2.4392922	2.3091587	1.7932268	0
112.01413	97.489999	46.447598	81.74227	86.42531	71.14269	142.14421	90.05719	116.30356	40.132946
1.8066794	1.2110559	1.6240419	2.4770385	4.4320672	1.8721761	7.0961227	1.1545794	4.3549793	3.2106357
**139.47565**	**70.846769**	**100.36579**	**106.51265**	**84.209276**	**67.398338**	**284.95368**	**286.33568**	**252.84497**	**96.31907**
6.1427101	1.5138199	1.6240419	0	0	0	5.7655997	8.4669153	5.3796803	0
5.4200383	3.6331677	4.5473173	2.4770385	11.080168	5.6165282	11.087692	15.394391	8.1976081	1.6053178
0	0	0	0	0	1.8721761	0.2217538	0	0.2561753	0
7.5880537	9.0829192	11.043485	9.9081539	6.6481007	13.105232	12.418215	11.545794	18.444618	8.0265892
0	0	0	0	0	0	0.8870153	0	0.2561753	0
6.504046	8.7801552	4.2225089	9.9081539	13.296201	7.4887042	26.832214	37.331399	18.188443	9.631907
0	0	0	0	0	0	0.2217538	0.3848598	0	0
**140.19833**	**72.966117**	**101.01541**	**104.03562**	**84.209276**	**67.398338**	**288.05823**	**289.79942**	**253.10115**	**97.924388**
6.1427101	1.5138199	1.6240419	0	0	0	5.7655997	8.4669153	5.3796803	0
0.7226718	0	0	0	0	0	0	1.9242989	0.2561753	0
2.5293512	2.4221118	1.6240419	4.9540769	0	1.8721761	3.1045537	5.388037	5.3796803	0
7.5880537	9.0829192	11.043485	9.9081539	6.6481007	13.105232	12.418215	11.545794	18.444618	8.0265892
0	0	0	0	0	0	0.8870153	0	0.2561753	0
6.504046	8.7801552	4.2225089	9.9081539	13.296201	7.4887042	26.832214	37.331399	18.188443	9.631907
0	0.302764	0	0	0	0	0.4435077	0	0	0
1366.5723	1084.8033	1382.7093	1317.7845	1001.6472	1729.8907	1700.8519	2412.686	2004.3152	515.30703
1.8066794	1.2110559	1.2992335	0	0	0	1.1087692	0.3848598	0.2561753	0
977.41358	1415.1188	955.58625	1020.5399	1163.4176	638.41204	1346.711	2834.4923	1956.4104	1521.8413
1164.9469	1383.3286	777.91607	1159.254	1283.0834	949.19326	737.77501	2297.9978	1156.8874	828.34401
1.4453436	1.8165838	1.9488503	0	4.4320672	1.8721761	0.2217538	0	0.2561753	0
2.1680153	2.4221118	1.6240419	0	2.2160336	1.8721761	0.2217538	0	0	0
3.252023	3.6331677	5.1969341	2.4770385	2.2160336	0	20.179599	7.6971957	19.98167	1.6053178
38.301604	70.241241	44.498748	14.862231	28.808437	20.593937	108.43762	71.58392	135.77288	40.132946
6.1427101	11.505031	4.5473173	0	2.2160336	5.6165282	1.330523	0.3848598	0.5123505	0
8.3107255	13.624379	10.06906	4.9540769	2.2160336	9.3608803	26.61046	17.31869	26.129876	3.2106357
0	0	0	0	0	0	0	0	0	0
38.301604	69.635713	44.498748	14.862231	28.808437	20.593937	108.21587	70.429341	134.74818	40.132946

7.9493896	5.7525155	8.1202095	4.9540769	6.6481007	3.7443521	12.418215	6.9274761	10.503185	1.6053178
8.3107255	13.624379	10.06906	4.9540769	2.2160336	9.3608803	26.61046	17.31869	26.129876	3.2106357
14.814771	30.579161	13.96676	4.9540769	2.2160336	33.699169	0.2217538	0.3848598	0	1.6053178
10.840077	22.10177	14.941185	9.9081539	2.2160336	9.3608803	33.706583	23.861307	35.352185	3.2106357
0	0	0	0	0	0	0	0	0	0
7.5880537	9.0829192	0.9744251	0	6.6481007	5.6165282	8.6483995	3.8485979	7.4290823	0
1240.8274	1196.826	1136.8293	1196.4096	1227.6826	1203.8092	1256.9007	1535.2057	1352.093	2171.995
2205.5943	784.76421	1241.7424	2506.7629	2575.031	2577.9864	2436.1876	3600.7482	1854.965	1303.5181
**1393.6725**	**1260.4064**	**1173.8575**	**916.50423**	**846.52483**	**825.62964**	**1281.0719**	**2224.8744**	**1455.0754**	**714.36644**
15.176107	14.229907	10.718677	14.862231	8.8641343	24.338289	15.522768	14.624672	15.882866	0
1516.8881	1566.8036	1625.9908	1285.583	1083.6404	1267.4632	1507.7043	1934.6901	1491.1961	593.9676
134.41695	70.241241	48.396449	148.62231	155.12235	93.608803	161.8803	186.657	170.35654	33.711675
33.965574	31.487453	35.079305	29.724462	26.592403	39.315697	31.489044	25.400746	28.179278	11.237225
0	0	0	0	0	0	0	0	0	0
0	0	0.3248084	0	0	0	0.8870153	0.7697196	0.2561753	0
0	0	0	0	0	0	0	0	0	0
15.898779	23.010062	24.360629	4.9540769	4.4320672	20.593937	28.606245	41.949717	39.194814	11.237225
21.318817	19.982422	24.360629	17.339269	11.080168	14.977408	33.928337	38.870838	44.062143	16.053178
1.4453436	0.6055279	0.9744251	0	0	1.8721761	0.6652615	1.9242989	0.5123505	3.2106357
2.8906871	0.9082919	2.9232754	0	0	9.3608803	3.1045537	4.6183174	1.024701	4.8159535
33.242902	44.20354	39.626622	49.540769	37.672571	43.060049	29.936768	63.117005	37.913937	25.685085
2184.2755	2231.6732	2045.968	1684.3862	1396.1012	1952.6796	1789.1099	1911.5986	1976.3921	634.10055
1.8066794	1.8165838	2.598467	4.9540769	2.2160336	0	2.8827998	3.8485979	4.8673298	3.2106357
1560.6097	1539.8576	1410.318	1107.2362	924.086	1318.0119	1103.0036	1056.825	1086.9516	407.75073
35.410917	39.66208	38.652197	27.247423	26.592403	35.571345	33.484829	58.113828	39.450989	30.501039
1560.6097	1539.8576	1410.318	1107.2362	924.086	1318.0119	1103.0036	1056.825	1086.9516	407.75073
18.066794	16.652018	15.915611	14.862231	8.8641343	11.233056	11.752953	27.325045	10.759361	17.658496
8349.3884	7963.9035	6978.1832	6596.3535	5821.5202	7537.3808	6637.314	7966.2127	6977.9577	3465.8812
194.39871	159.55661	142.91569	222.93346	177.28269	215.30025	153.01015	281.71736	170.10037	61.002078
57.813742	37.845496	43.524323	61.925962	64.264974	74.887042	52.555659	99.293825	71.472895	22.47445
8.6720613	6.6608074	1.9488503	4.9540769	6.6481007	5.6165282	1.1087692	0.3848598	0.2561753	0
398.19215	489.26658	450.83403	391.37208	358.99744	389.41262	726.68731	872.09227	771.59986	378.85501
698.10094	638.52922	568.41467	802.56047	673.67421	803.16353	774.8079	1077.2225	652.22219	308.22103
1247.3315	841.07831	791.55802	918.98127	784.47589	1216.9144	730.67888	1022.5725	760.58432	235.98172
698.10094	638.52922	568.41467	802.56047	673.67421	803.16353	774.8079	1077.2225	652.22219	308.22103
**1247.3315**	**841.07831**	**791.55802**	**918.98127**	**780.04382**	**1216.9144**	**730.67888**	**1022.5725**	**760.58432**	**234.3764**
29.268207	32.395745	10.393868	32.2015	39.888604	28.082641	32.154306	28.864484	39.707164	11.237225
0	0	0	0	0	0	0.2217538	0	0	0
**5334.0404**	**8080.1649**	**9292.1181**	**3869.1341**	**3315.1862**	**4996.8379**	**5448.9352**	**5216.7744**	**5499.3141**	**2587.7724**
22.041489	32.092981	27.283904	17.339269	24.376369	24.338289	19.957845	33.482801	22.031072	8.0265892
0	0	0	0	0	0	0	0	0	0
0	0.6055279	0	0	0	0	0	0	1.024701	0
0	0	0	0	0	0	0	0	0	0
6.1427101	9.9912111	5.5217425	0	2.2160336	5.6165282	7.3178765	6.1577566	7.9414328	0
**553.92792**	**505.9186**	**538.20749**	**383.94096**	**301.38057**	**466.17184**	**545.95794**	**562.28015**	**516.44931**	**109.16161**
0	1.5138199	0.6496168	0	0	0	1.330523	0.3848598	0.2561753	0
**124.66088**	**133.21615**	**139.99241**	**153.57639**	**150.69028**	**234.02201**	**93.801872**	**205.89999**	**119.12149**	**75.449938**

0	0	0	0	0	0	0	0	0	0
0	0	0.3248084	0	0	0	0	0	0	0
7.9493896	5.7525155	6.4961676	0	4.4320672	7.4887042	3.1045537	3.8485979	1.7932268	0
1.4453436	0.6055279	0	0	0	0	0.6652615	3.8485979	1.5370515	3.2106357
0	0	0.3248084	0	0	1.8721761	0.6652615	0	0.2561753	0
0	0	0	0	0	1.8721761	0	0	0.2561753	0
57.452406	39.66208	32.480838	47.063731	48.752739	50.548754	64.308612	78.896256	54.821504	11.237225
14.814771	11.505031	11.693102	14.862231	15.512235	9.3608803	19.736091	25.785606	13.833464	3.2106357
0	0	0	0	0	0	0	0.3848598	0	0
14.814771	11.505031	11.693102	14.862231	15.512235	9.3608803	19.736091	25.785606	13.577288	3.2106357
0	0	0	0	0	0	0	0	0.5123505	0
**97.56069**	**89.315372**	**75.680353**	**66.880039**	**117.44978**	**112.33056**	**185.16445**	**235.14933**	**158.31631**	**93.108435**
0	0.6055279	0	0	0	0	0	0	0.2561753	0
0	0	0	0	0	0	0	0	0	0
0.3613359	0	0	0	0	0	0	0	0	0
0	0	0	0	0	0	0	0	0	0
0.3613359	0	0	0	0	0	0	0	0	0
0	0	0	0	0	0	0	0	0	0
0.3613359	0	0	0	0	0	0	0	0	0
2.5293512	2.1193478	1.9488503	0	0	0	2.2175383	3.4637381	1.7932268	0
**166.57584**	**98.095527**	**92.24558**	**104.03562**	**97.505477**	**97.353155**	**205.1223**	**220.52466**	**230.55773**	**40.132946**
0	0	0	0	0	0	0	0	0.2561753	0
0.3613359	0.302764	0	0	0	0	4.2133228	14.239812	6.148206	1.6053178
0	0.302764	0.3248084	0	0	0	0.4435077	0	2.049402	0
11.562748	14.229907	5.1969341	4.9540769	2.2160336	1.8721761	2.661046	1.5394391	6.148206	0
0.3613359	0.6055279	0	0	0	0	0	0	0	0
7.9493896	8.4773912	11.368293	7.4311154	0	7.4887042	0	0	0.5123505	0
0.3613359	0.6055279	0	0	0	0	0	0	0	0
7.9493896	8.4773912	11.368293	7.4311154	0	7.4887042	0	0	0.5123505	0
0.3613359	0.6055279	0	0	0	0	0	0	0	0
7.9493896	8.4773912	11.368293	7.4311154	0	7.4887042	0	0	0.5123505	0
0.3613359	0.6055279	0	0	0	0	0	0	0	0
7.9493896	8.4773912	11.368293	7.4311154	0	7.4887042	0	0	0.5123505	0
0.3613359	0.6055279	0	0	0	0	0	0	0	0
7.9493896	8.4773912	11.368293	7.4311154	0	7.4887042	0	0	0.5123505	0
0.3613359	0.6055279	0	0	0	0	0	0	0	0
7.9493896	8.4773912	11.368293	7.4311154	0	7.4887042	0	0	0.5123505	0
**14.453436**	**10.596739**	**12.342718**	**9.9081539**	**2.2160336**	**5.6165282**	**17.740307**	**8.0820555**	**13.321113**	**1.6053178**
0	0	0	0	0	0	0	0	0	0
0	0	0	0	0	0	3.1045537	5.0031772	10.24701	1.6053178
0	0	0	0	0	0	0	0.3848598	0.5123505	0
0	0	0	0	0	0	0	0	0	0
1.8066794	4.5414596	7.4705927	4.9540769	2.2160336	3.7443521	2.4392922	1.9242989	3.8426288	0
1.8066794	0.302764	0.6496168	0	0	0	0	0	0.5123505	0
1.8066794	3.3304037	1.9488503	7.4311154	0	5.6165282	0.6652615	0.7697196	1.5370515	3.2106357

5.4200383	6.9635713	7.7954011	9.9081539	6.6481007	3.7443521	25.279937	26.170465	23.311948	8.0265892
0	0	0	0	0	0	0	0	0	0
0	0	0	0	0	0	0	0.3848598	0	0
1.4453436	3.0276397	1.6240419	2.4770385	0	0	0.8870153	1.1545794	1.2808763	0
6.504046	4.8442235	2.2736587	9.9081539	0	5.6165282	4.6568305	11.545794	6.4043813	3.2106357
1.4453436	1.8165838	1.6240419	0	0	0	0.8870153	0.3848598	1.7932268	1.6053178
1.0840077	0.9082919	0.6496168	0	0	1.8721761	0	0	0	0
0	0	0	0	0	0	0	0	0	0
1.0840077	0.9082919	0.6496168	0	0	1.8721761	0	0	0	0
0	0	0	0	0	0	0	0	0	0
0.3613359	0.9082919	0.3248084	0	0	1.8721761	0.2217538	0	0.5123505	0
**83.829926**	**98.701055**	**98.416939**	**59.448923**	**48.752739**	**82.375746**	**110.65516**	**127.77345**	**113.99799**	**30.501039**
2.8906871	2.7248757	1.9488503	0	0	3.7443521	1.330523	0.7697196	1.5370515	1.6053178
1.8066794	2.4221118	0.6496168	0	0	1.8721761	3.1045537	5.0031772	2.8179278	0
0	0	0	0	0	0	0	0	0	0
0	0	0	0	0	0	0	0	0	0
0.7226718	1.2110559	2.598467	0	0	0	0	0	0	0
10.478741	19.982422	14.291569	2.4770385	0	1.8721761	0.8870153	0.3848598	0	0
1.8066794	0.6055279	2.9232754	0	0	1.8721761	0.8870153	1.1545794	1.5370515	0
8.3107255	8.4773912	10.718677	2.4770385	6.6481007	7.4887042	9.0919072	8.4669153	8.9661338	1.6053178
**59.620422**	**43.900776**	**37.677772**	**22.293346**	**15.512235**	**50.548754**	**32.154306**	**53.11065**	**35.096009**	**12.842543**
0	0	0	0	0	0	0	0	0	0
**87.081949**	**63.580434**	**52.618958**	**42.109654**	**28.808437**	**63.653986**	**43.020244**	**69.659621**	**51.23505**	**22.47445**
0	0	0	0	0	0	0	0	0	0
0	0	0	0	0	0	0	0	0	0
49.141681	45.111832	50.020491	29.724462	22.160336	37.443521	0	0	0	0
0	0	0	0	0	0	0.4435077	0.3848598	0	0
6.504046	6.9635713	9.419443	4.9540769	13.296201	7.4887042	5.322092	7.6971957	4.3549793	3.2106357
0	0	0	0	0	0	0.4435077	0.3848598	0	0
6.504046	6.9635713	9.419443	4.9540769	13.296201	7.4887042	5.322092	7.6971957	4.3549793	3.2106357
41.914963	67.213602	61.388784	14.862231	13.296201	31.826993	0	0	0.5123505	1.6053178
2.5293512	4.5414596	3.2480838	0	0	5.6165282	0	0	0	0
0	0	0	2.4770385	0	0	0.2217538	0	0.2561753	0
0	0	0	0	0	0	0	0	0	0
14.453436	13.927143	10.718677	14.862231	4.4320672	5.6165282	13.970492	12.700373	13.321113	4.8159535
0	0	0	2.4770385	0	0	0	0	0	0
3.6133589	4.5414596	4.2225089	2.4770385	2.2160336	7.4887042	7.0961227	9.6214946	9.9908348	0
0	0	0	0	0	0	8.2048919	4.2334576	7.172907	1.6053178
2.1680153	5.1469875	2.9232754	0	2.2160336	1.8721761	1.7740307	1.9242989	2.049402	1.6053178
5.4200383	3.3304037	1.9488503	0	2.2160336	1.8721761	3.7698152	3.0788783	1.2808763	1.6053178
2.1680153	5.1469875	2.9232754	0	2.2160336	1.8721761	1.7740307	1.9242989	2.049402	1.6053178
5.4200383	3.3304037	1.9488503	0	2.2160336	1.8721761	3.7698152	3.0788783	1.2808763	1.6053178
2.1680153	5.1469875	2.9232754	0	2.2160336	1.8721761	1.7740307	1.9242989	2.049402	1.6053178
5.4200383	3.3304037	1.9488503	0	2.2160336	1.8721761	3.7698152	3.0788783	1.2808763	1.6053178
2.1680153	5.1469875	2.9232754	0	2.2160336	1.8721761	1.7740307	1.9242989	2.049402	1.6053178
5.4200383	3.3304037	1.9488503	0	2.2160336	1.8721761	3.7698152	3.0788783	1.2808763	1.6053178
1.4453436	0.6055279	0.6496168	0	0	0	0	0	0	0

0	0.302764	0	0	0	0	0	0	0	0
9.756069	5.4497515	6.820976	2.4770385	4.4320672	1.8721761	0.6652615	0.3848598	0.7685258	0
0	0	0.3248084	0	2.2160336	0	0	0	0	0
0.3613359	0	0.6496168	0	0	0	3.3263075	5.7728968	3.3302783	3.2106357
0	0	0	0	0	0	0.4435077	0.7697196	0.7685258	0
**5.4200383**	**2.4221118**	**3.8977006**	**2.4770385**	**2.2160336**	**0**	**27.940983**	**51.186351**	**27.923102**	**8.0265892**
0	0.6055279	0.6496168	0	0	0	0.4435077	1.1545794	0.2561753	0
0	0.302764	0	0	0	0	0	0	0	0
2.1680153	3.0276397	1.2992335	0	2.2160336	5.6165282	1.1087692	3.8485979	1.7932268	1.6053178
0.3613359	0.9082919	2.2736587	0	0	0	1.330523	0.7697196	3.3302783	0
23.848169	13.927143	18.838886	7.4311154	19.944302	29.954817	0	0	0	0

132_S_Pu_i	132_S_Pu_i	161_R_CK_	161_R_CK_	161_R_CK_	161_R_Pu_i	161_R_Pu_i	161_R_Pu_i	164_R_CK_	164_R_CK_
4.5765438	4.1039211	8.9969682	4.5443832	5.4075843	3.4795722	0	1.6028125	2.7144866	2.6969102
0	0	0	0	0	0	0	0	0	0
540.03217	478.10681	495.17929	478.39962	476.89744	186.15711	216.39009	200.35156	441.25488	438.05528
27.459263	6.1558817	16.26375	25.200671	28.84045	12.178503	8.7963451	11.219687	22.319112	16.374098
0	0	0.6920745	0	0	0	0	0	0	0
139.58459	141.58528	189.62841	167.31593	111.75674	80.030161	42.222456	67.318124	68.766994	57.98357
0	0	0	0	0	0	0	0	0	0
0	2.0519606	0	0.4131257	0	0	0	0	0.6032192	0
0	0	0	0	0	0	0	0	0	0
0	2.0519606	0	0.4131257	0	0	0	0	0.6032192	0
13.729632	10.259803	43.946729	35.94194	35.27805	29.576364	22.870497	30.453437	35.891545	32.170286
0	0	0.6920745	0.8262515	0.772512	0	0	4.8084375	1.5080481	0.3852729
4.5765438	2.0519606	6.9207447	8.262515	5.4075843	6.9591444	5.2778071	0	8.1434598	7.8980942
0	0	0	0	0	0	0	0	0	0
11.44136	8.2078423	19.378085	25.200671	29.355458	13.918289	17.59269	24.042187	27.144866	24.464829
0	0	0	0	0	0	0	0	0.3016096	0
4.5765438	8.2078423	19.378085	15.285653	21.630337	5.2193583	1.759269	4.8084375	17.191748	15.603552
0	0	0	0	0	0	0	0	0	0
0	0	0	0	0	0	0	0	0	0
0	0	5.1905586	3.305006	3.3475522	1.7397861	0	4.8084375	4.5241443	2.1190009
10397.908	8384.3109	26516.141	22742.159	35107.84	12380.318	12497.847	13694.43	29916.658	28613.64
13.729632	24.623527	14.187527	4.957509	22.402849	5.2193583	12.314883	14.425312	15.080481	21.960555
270.01609	283.17056	888.62362	977.0424	1013.0208	407.10995	427.50237	487.255	1087.6043	1172.9633
0	0	0	0	0	0	0	0	0	0
2.2882719	6.1558817	7.9588564	7.4362635	5.6650883	8.6989305	3.518538	8.0140624	6.9370213	9.2465494
0	0	0	0	0	0	0	0	0	0
0	0	0	0	0.257504	1.7397861	0	1.6028125	0.6032192	0
2.2882719	0	4.4984841	4.957509	10.300161	1.7397861	0	1.6028125	4.5241443	2.8895467
6.8648158	22.571566	33.219575	28.505677	32.188002	33.055936	29.907573	36.864687	21.715893	15.218279
0	0	0	0	0	0	0	0	0	0
164.75558	119.01371	42.56258	40.899449	105.31914	67.851658	84.444913	49.687187	79.32333	49.700203
395.87104	350.88526	384.10133	419.73576	613.88957	295.76364	311.39062	237.21625	420.1422	280.86394
0	0	0.3460372	0	0.257504	0	0	0	0.6032192	0.5779093
9.1530877	4.1039211	3.8064096	2.0656287	4.8925763	3.4795722	1.759269	1.6028125	3.6193155	2.6969102
43.477166	20.519606	47.061064	24.787545	47.638243	111.34631	131.94518	91.360312	26.240037	26.198557
1185.3249	804.36854	1484.4997	1479.8164	2877.3499	1327.4568	1308.8961	1168.4503	1459.489	1161.4051
139.58459	112.85783	74.051969	48.335713	96.048997	121.78503	182.96398	137.84187	60.623534	38.912562
2134.9577	1541.0224	1061.9883	973.73739	1702.874	984.71894	1206.8585	976.1128	1365.3868	789.23151
164.75558	119.01371	42.56258	40.899449	105.31914	67.851658	84.444913	49.687187	79.32333	49.700203
398.15931	352.93722	386.52359	421.38826	618.78215	300.983	309.63135	237.21625	421.95186	283.36821
675.04022	439.11956	2723.6591	364.79004	3535.7876	1214.3707	1016.8575	1299.8809	3224.5085	2263.4782
208.23274	164.15685	206.93027	191.69035	345.57039	180.93776	165.37129	136.23906	228.62009	158.73243
6.8648158	22.571566	31.489389	27.679425	31.672994	31.31615	29.907573	36.864687	21.715893	14.06246
208.23274	164.15685	206.93027	191.69035	345.57039	180.93776	165.37129	136.23906	228.62009	158.73243
43.477166	20.519606	47.061064	24.787545	47.638243	111.34631	131.94518	91.360312	26.240037	26.198557
1185.3249	804.36854	1484.4997	1479.8164	2877.3499	1327.4568	1308.8961	1168.4503	1459.489	1161.4051

139.58459	112.85783	74.051969	48.748838	96.306501	121.78503	182.96398	137.84187	60.925143	38.719925
135.00804	108.75391	208.31442	203.67099	291.23704	172.23882	172.40836	134.63625	131.8034	105.56477
11.44136	10.259803	1.7301862	0.8262515	4.1200642	1.7397861	3.518538	1.6028125	0.3016096	1.1558187
9.1530877	4.1039211	3.8064096	2.0656287	4.8925763	3.4795722	1.759269	1.6028125	3.6193155	2.6969102
0	0	0	0	0	0	0	0	0	0
4.5765438	0	4.8445213	3.305006	5.6650883	1.7397861	3.518538	3.205625	3.0160962	1.733728
139.58459	112.85783	74.051969	48.335713	96.048997	121.78503	182.96398	137.84187	60.623534	38.912562
2134.9577	1541.0224	1061.9883	973.73739	1702.874	984.71894	1206.8585	976.1128	1365.3868	789.23151
164.75558	119.01371	42.56258	40.899449	105.31914	67.851658	84.444913	49.687187	79.32333	49.700203
395.87104	350.88526	384.10133	419.73576	613.88957	295.76364	311.39062	237.21625	420.1422	280.86394
334.0877	201.09214	188.59029	135.91837	410.97641	160.06032	181.20471	184.32344	278.9889	131.18542
208.23274	164.15685	206.93027	191.69035	345.57039	180.93776	165.37129	136.23906	228.62009	158.73243
331.79943	203.1441	188.24426	135.09212	409.17388	160.06032	179.44544	187.52906	278.08407	130.80015
208.23274	164.15685	206.93027	191.69035	345.57039	180.93776	165.37129	136.23906	228.62009	158.73243
0	0	0	0.4131257	1.5450241	0	0	0	1.2064385	0.1926364
395.87104	350.88526	384.10133	419.73576	613.88957	295.76364	311.39062	237.21625	420.1422	280.86394
0	0	0	0.4131257	1.5450241	0	0	0	1.2064385	0.1926364
395.87104	350.88526	384.10133	419.73576	613.88957	295.76364	311.39062	237.21625	420.1422	280.86394
6.8648158	2.0519606	1.7301862	0	7.2101124	0	10.555614	1.6028125	7.5402405	2.1190009
395.87104	350.88526	384.10133	419.73576	613.88957	295.76364	311.39062	237.21625	420.1422	280.86394
6.8648158	2.0519606	4.4984841	3.7181317	4.1200642	6.9591444	3.518538	1.6028125	3.0160962	1.5410916
208.23274	164.15685	207.2763	192.10347	345.57039	180.93776	165.37129	136.23906	228.9217	159.1177
4.5765438	0	3.4603724	2.8918802	4.8925763	1.7397861	3.518538	3.205625	2.412877	1.5410916
0	0	0	0	0	0	0	0	0	0
13548.858	11583.317	25184.244	24294.273	28658.909	11430.395	10346.261	10349.36	24888.524	20814.946
192.21484	100.54607	40.832394	15.285653	250.2939	66.111872	128.42664	110.59406	196.04625	71.082848
18.306175	10.259803	91.699868	82.62515	111.75674	13.918289	8.7963451	8.0140624	80.529769	58.561479
0	0	0	0	0	0	0	0	0	0
13548.858	11583.317	25184.244	24294.273	28659.167	11430.395	10346.261	10349.36	24888.524	20814.753
192.21484	100.54607	40.832394	15.285653	250.2939	66.111872	128.42664	110.59406	196.04625	71.082848
0	2.0519606	2.0762234	1.652503	17.252769	12.178503	7.0370761	8.0140624	10.556337	10.209732
402.73586	396.02839	310.04936	298.27679	375.95586	340.99808	279.72377	317.35687	274.16315	226.15519
16.017903	6.1558817	15.917713	2.8918802	43.260674	13.918289	21.111228	9.6168749	36.193155	20.997373
405.02413	400.13231	315.58596	300.75555	379.04591	340.99808	283.24231	320.5625	277.17924	227.69628
9.1530877	8.2078423	4.1524468	1.2393772	27.037922	12.178503	5.2778071	9.6168749	26.843256	14.64037
402.73586	396.02839	310.04936	298.27679	375.95586	340.99808	279.72377	317.35687	274.16315	226.15519
4.5765438	0	1.7301862	0.4131257	15.450241	5.2193583	8.7963451	1.6028125	16.890139	7.3201849
405.02413	400.13231	315.58596	300.75555	379.04591	340.99808	283.24231	320.5625	277.17924	227.69628
457.65438	549.92543	1608.035	1352.1606	1487.6007	873.37263	793.43033	1166.8475	1479.6968	883.62337
86.954333	67.714699	103.46513	30.15818	106.09165	43.494653	63.333685	78.537812	117.62775	47.966475
457.65438	549.92543	1608.035	1352.1606	1487.6007	873.37263	793.43033	1166.8475	1479.6968	883.62337
86.954333	67.714699	103.46513	30.15818	106.09165	43.494653	63.333685	78.537812	117.62775	47.966475
2.2882719	0	0.3460372	0	1.5450241	0	0	0	0.9048289	2.8895467
260.863	172.36469	333.92593	365.61629	426.42665	240.09048	258.61255	232.40781	372.78949	240.79556
247.13337	164.15685	287.21091	204.08412	336.81525	222.69262	151.29714	193.94031	338.406	152.18279
260.863	172.36469	333.92593	365.61629	426.94166	240.09048	258.61255	232.40781	373.39271	240.98819
0	0	0	0	0.772512	0	0	0	0.3016096	0.1926364

260.863	172.36469	333.92593	365.61629	426.42665	240.09048	258.61255	232.40781	372.78949	240.79556
0	0	0.3460372	0.4131257	3.8625602	0	0	1.6028125	1.2064385	1.3484551
0	0	2.4222607	1.2393772	3.3475522	0	3.518538	8.0140624	1.5080481	1.733728
0	0	3.4603724	3.7181317	4.6350723	0	7.0370761	0	8.746679	4.8159111
0	0	0	0	0.257504	0	0	0	0.3016096	0
247.13337	164.15685	287.21091	204.08412	336.81525	222.69262	151.29714	193.94031	338.406	152.18279
260.863	172.36469	333.92593	365.61629	426.94166	240.09048	258.61255	232.40781	373.39271	240.98819
0	0	0	0.4131257	0	0	0	0	0	0
260.863	172.36469	333.92593	365.61629	426.42665	240.09048	258.61255	232.40781	372.78949	240.79556
0	0	0	0	0.515008	0	0	1.6028125	0	0
4.5765438	10.259803	16.609787	24.374419	15.192737	10.438717	12.314883	11.219687	8.4450694	9.4391858
286528.26	234450.86	592218.16	601888.69	577879.18	196630.63	200141.48	233353.47	604918.81	651381.07
1265.4144	810.52442	623.90514	216.06477	2749.3704	720.27145	844.44913	777.36406	1364.7835	1200.3177
**286880.65**	**234758.65**	**592682.2**	**602248.52**	**578464.49**	**196860.28**	**200357.87**	**233563.44**	**605102.49**	**651839.16**
9.1530877	16.415685	3.8064096	3.305006	10.300161	5.2193583	3.518538	12.8225	3.9209251	5.3938205
286743.35	234672.47	592539.28	602272.89	578296.34	196726.31	200275.18	233496.12	605045.49	651936.44
32.035807	24.623527	26.644867	31.810683	84.461317	34.795722	54.53734	35.261875	88.974838	77.054578
286528.26	234450.86	592218.16	601888.69	577879.18	196630.63	200141.48	233353.47	604918.81	651381.07
1109.8119	728.446	317.31615	154.0959	2321.1412	608.92514	740.65226	663.56437	866.22283	585.03688
286528.26	234450.86	592218.16	601888.69	577879.18	196630.63	200141.48	233353.47	604918.81	651381.07
1267.7026	810.52442	627.36551	216.06477	2761.2155	718.53166	847.96767	786.98093	1374.7367	1205.1336
14908.092	14220.087	15142.589	17301.293	16258.288	8203.0915	7716.1539	7823.3278	17523.519	13335.451
0	2.0519606	0.3460372	1.2393772	2.3175361	1.7397861	0	0	0.3016096	0.9631822
**286880.65**	**234758.65**	**592682.2**	**602248.52**	**578464.49**	**196860.28**	**200357.87**	**233563.44**	**605102.49**	**651839.16**
0	2.0519606	0.3460372	0	1.8025281	1.7397861	0	3.205625	0.9048289	0.5779093
286896.67	234709.41	592648.29	602260.09	578415.3	196818.52	200327.96	233532.99	605099.48	651859.77
0	0	0	0	0.257504	0	1.759269	1.6028125	0.3016096	0
286896.67	234709.41	592648.29	602260.09	578415.3	196818.52	200327.96	233532.99	605099.48	651859.77
20.594447	14.363724	40.832394	8.6756407	59.483427	17.397861	21.111228	17.630937	38.304422	33.904014
286761.66	234623.22	592505.37	602284.05	578248.18	196688.04	200245.28	233467.27	605044.58	651958.78
0	0	0	0	0.257504	0	1.759269	1.6028125	0.3016096	0
9.1530877	22.571566	7.266782	3.7181317	10.300161	13.918289	17.59269	11.219687	9.9531175	3.6600925
407.3124	404.23623	210.39064	140.04963	204.20068	344.47765	362.40942	299.72594	126.67604	102.09732
0	0	0	0	0	0	0	0	0	0.3852729
368.41178	387.82055	196.20311	131.78711	190.81047	327.07979	353.61307	285.30062	117.02453	93.621312
2.2882719	0	0.6920745	0.8262515	2.3175361	0	0	3.205625	0.9048289	1.9263644
405.02413	404.23623	209.69857	137.57087	203.42817	342.73786	362.40942	296.52031	126.07282	101.71204
9.1530877	22.571566	7.266782	3.7181317	10.300161	13.918289	17.59269	11.219687	9.9531175	3.6600925
407.3124	404.23623	210.39064	140.04963	204.20068	344.47765	362.40942	299.72594	126.67604	102.09732
2.2882719	2.0519606	1.0381117	0.4131257	2.5750401	3.4795722	1.759269	4.8084375	5.1273636	1.733728
368.41178	387.82055	196.20311	131.78711	190.81047	327.07979	353.61307	285.30062	117.02453	93.621312
9.1530877	22.571566	7.266782	3.7181317	10.300161	13.918289	17.59269	11.219687	9.9531175	3.6600925
405.02413	404.23623	209.69857	137.57087	203.42817	340.99808	362.40942	296.52031	126.07282	101.71204
4.5765438	6.1558817	2.0762234	1.652503	5.9225923	0	7.0370761	14.425312	3.3177058	2.8895467
173.90867	205.19606	56.750107	40.073198	61.285955	210.51412	232.22351	153.87	39.51086	41.802109
4.5765438	6.1558817	2.0762234	1.652503	5.9225923	0	7.0370761	14.425312	3.3177058	2.8895467
173.90867	205.19606	56.750107	40.073198	61.285955	210.51412	232.22351	153.87	39.51086	41.802109

1210.4958	1003.4087	440.5054	223.0879	2733.4051	939.4845	990.46846	891.16374	3322.8332	1629.3191
3652.082	2981.4987	5998.2095	6095.2573	5367.4137	2326.094	2628.3479	2461.92	6244.8272	3465.1444
1210.4958	1003.4087	440.5054	223.0879	2733.4051	939.4845	990.46846	891.16374	3322.8332	1629.3191
3652.082	2981.4987	5998.2095	6095.2573	5367.4137	2326.094	2628.3479	2461.92	6244.8272	3465.1444
0	0	0	0	0	0	0	0	0	0.1926364
6.8648158	16.415685	31.143351	24.374419	41.973154	41.754867	59.815147	64.112499	31.367401	23.30901
0	0	0	0.4131257	1.8025281	1.7397861	1.759269	1.6028125	1.8096577	1.9263644
6.8648158	16.415685	29.759202	24.374419	40.170626	41.754867	59.815147	64.112499	31.367401	22.923737
0	2.0519606	0	0	2.3175361	1.7397861	1.759269	1.6028125	1.5080481	0.1926364
0	0	0	0	0.772512	0	3.518538	0	1.2064385	1.5410916
2.2882719	4.1039211	2.0762234	1.2393772	6.6951044	15.658075	36.944649	20.836562	4.5241443	6.5496391
189.92657	254.44311	773.04719	828.73025	1621.7603	812.48011	918.33843	1088.3097	779.35926	760.52868
0	4.1039211	4.1524468	1.2393772	20.342817	5.2193583	10.555614	24.042187	14.778871	6.5496391
6.8648158	16.415685	31.143351	24.374419	41.973154	41.754867	59.815147	64.112499	31.367401	23.30901
0	0	0	0	0	0	0	0	0	0
0	2.0519606	6.5747075	4.5443832	12.360193	5.2193583	14.074152	8.0140624	15.98531	3.0821831
0	4.1039211	3.1143351	1.2393772	4.8925763	1.7397861	1.759269	3.205625	3.9209251	2.8895467
6.8648158	16.415685	31.143351	24.787545	41.973154	41.754867	59.815147	64.112499	31.367401	23.30901
0	2.0519606	0	0	0.772512	1.7397861	1.759269	1.6028125	0.9048289	1.3484551
6.8648158	16.415685	29.759202	24.374419	40.170626	41.754867	59.815147	64.112499	31.367401	22.923737
0	4.1039211	3.1143351	1.2393772	4.8925763	1.7397861	1.759269	3.205625	3.9209251	2.8895467
6.8648158	16.415685	31.143351	24.787545	41.973154	41.754867	59.815147	64.112499	31.367401	23.30901
0	4.1039211	5.1905586	2.8918802	2.8325442	5.2193583	7.0370761	0	3.9209251	2.6969102
0	2.0519606	6.5747075	4.5443832	12.360193	5.2193583	14.074152	8.0140624	15.98531	3.0821831
0	0	0	0	0.772512	0	0	0	0	0
6.8648158	16.415685	31.143351	24.374419	41.973154	41.754867	59.815147	64.112499	31.367401	23.30901
0	0	0	0	0	0	0	0	0	0
0	2.0519606	6.5747075	4.5443832	12.360193	5.2193583	14.074152	8.0140624	15.98531	3.0821831
144.16113	92.338225	154.33261	225.97978	148.06481	113.0861	80.926375	155.47281	142.96296	203.23145
6.8648158	2.0519606	1.3841489	0.8262515	1.2875201	8.6989305	3.518538	6.4112499	0	0.9631822
107.54878	86.182344	227.00043	348.26501	263.1691	69.591444	84.444913	70.523749	251.54242	87.842219
16.017903	8.2078423	7.266782	9.5018922	29.355458	13.918289	7.0370761	11.219687	22.017502	9.0539129
22.882719	28.727448	113.15418	90.061413	78.796228	24.357006	31.666842	40.070312	76.608844	57.98357
11.44136	24.623527	29.759202	22.721916	44.033187	6.9591444	19.351959	16.028125	38.002812	52.397113
73.224701	75.922541	141.87527	155.33528	128.4945	73.071017	42.222456	67.318124	87.46679	62.992117
0	0	0	0	0	0	0	0	0	0
22.882719	28.727448	111.77003	89.235162	77.508708	24.357006	31.666842	40.070312	75.402405	56.442478
4.5765438	6.1558817	18.686011	9.5018922	24.720385	5.2193583	12.314883	6.4112499	19.906235	10.402368
107.54878	86.182344	227.00043	348.26501	263.1691	69.591444	84.444913	70.523749	251.54242	87.842219
16.017903	8.2078423	7.266782	9.5018922	29.355458	13.918289	7.0370761	11.219687	22.017502	9.0539129
73.224701	75.922541	142.91338	155.74841	129.26702	73.071017	42.222456	67.318124	87.7684	63.37739
0	0	0	0	0.257504	0	0	0	0.3016096	0.1926364
0	0	2.7682979	1.652503	3.6050562	1.7397861	0	1.6028125	4.2225347	3.467456
0	0	0	0	0	0	0	0	0	0
0	0	0.3460372	1.2393772	1.0300161	0	5.2778071	4.8084375	1.2064385	3.467456
0	0	3.4603724	1.652503	2.3175361	1.7397861	3.518538	4.8084375	2.412877	4.0453653
0	0	2.7682979	1.652503	3.6050562	1.7397861	0	1.6028125	4.2225347	3.467456

2.2882719	0	0	0	0.515008	0	0	1.6028125	0.3016096	0.3852729
0	0	0.3460372	1.2393772	1.0300161	0	5.2778071	4.8084375	1.2064385	3.467456
0	0	0	0	0	0	0	0	0	0.1926364
0	0	0.3460372	1.2393772	1.0300161	0	5.2778071	4.8084375	1.2064385	3.467456
0	0	0	0	0	0	0	0	0	0
0	0	2.7682979	1.652503	3.6050562	1.7397861	0	1.6028125	4.2225347	3.467456
0	0	0	0	0	0	0	0	0	0
0	0	0.3460372	1.2393772	1.0300161	0	5.2778071	4.8084375	1.2064385	3.467456
0	0	0	0	0	0	0	0	0	0.1926364
2.2882719	4.1039211	11.765266	11.567521	7.7251204	6.9591444	5.2778071	6.4112499	8.4450694	12.521369
4.5765438	12.311763	13.841489	9.5018922	18.540289	8.6989305	10.555614	12.8225	14.477262	13.484551
4.5765438	12.311763	13.841489	9.5018922	18.540289	8.6989305	10.555614	12.8225	14.477262	13.484551
0	0	0	0	0	0	0	0	0	0
0	0	0	0	0	0	0	0	0	0
4.5765438	12.311763	13.841489	9.5018922	18.540289	8.6989305	10.555614	12.8225	14.477262	13.484551
2400.3972	2218.1694	3488.7474	3633.441	4294.652	2348.7112	2334.55	2316.064	3367.4714	2410.4598
11.44136	14.363724	6.9207447	6.610012	26.265409	15.658075	8.7963451	17.630937	18.398187	21.575282
2400.3972	2218.1694	3488.7474	3633.441	4294.652	2348.7112	2334.55	2316.064	3367.4714	2410.4598
6.8648158	6.1558817	5.882633	0.4131257	26.780418	6.9591444	7.0370761	16.028125	21.715893	19.456281
17317.642	15674.927	44317.681	39978.179	52978.361	22608.52	22747.348	22205.364	39179.693	31291.479
29.747535	18.467645	40.486357	41.312575	127.72199	33.055936	47.500263	40.070312	125.4696	48.351748
235.69201	254.44311	619.75269	600.27171	769.6795	334.03893	360.65015	351.01593	747.69025	706.59048
4.5765438	6.1558817	10.727154	4.957509	16.480257	5.2193583	7.0370761	4.8084375	13.874043	7.3201849
59.49507	53.350975	106.92551	99.976431	140.33969	53.933369	56.296609	56.098437	117.62775	117.70087
0	0	0	0	0	0	0	0	0	0
425.61858	445.27544	1144.3451	1395.1257	1387.1741	518.45626	633.33685	726.07406	1425.7087	1508.3434
0	0	3.1143351	2.0656287	2.5750401	1.7397861	1.759269	0	3.9209251	1.5410916
423.33031	445.27544	1139.5006	1390.9944	1381.2515	514.97669	626.29977	721.26562	1420.8829	1501.6011
0	2.0519606	1.3841489	1.2393772	4.8925763	5.2193583	0	0	3.0160962	2.3116373
17331.372	15693.394	44389.657	40033.538	53066.17	22658.974	22782.534	22250.243	39263.842	31357.168
6.8648158	8.2078423	22.146383	5.3706347	18.025281	15.658075	19.351959	22.439375	18.096577	15.025643
235.69201	254.44311	619.75269	600.27171	769.6795	334.03893	360.65015	351.01593	747.69025	706.78312
0	4.1039211	11.419229	7.4362635	4.8925763	8.6989305	8.7963451	3.205625	5.1273636	4.8159111
425.61858	445.27544	1144.3451	1395.1257	1387.1741	518.45626	633.33685	726.07406	1425.7087	1508.3434
0	0	3.1143351	2.0656287	2.8325442	1.7397861	1.759269	0	3.9209251	1.5410916
0	0	0	0	0	0	0	0	0	0
226.53892	227.76762	561.61844	538.30285	706.33351	311.42171	327.22404	310.94562	677.41521	626.64636
423.33031	445.27544	1139.5006	1390.9944	1381.2515	514.97669	626.29977	721.26562	1420.8829	1501.6011
0	4.1039211	11.419229	7.4362635	4.8925763	8.6989305	8.7963451	3.205625	5.1273636	4.8159111
226.53892	227.76762	561.61844	538.30285	706.33351	311.42171	327.22404	310.94562	677.41521	626.64636
0	0	0	0	0	0	0	0	0	0
6.8648158	14.363724	1.7301862	0.8262515	1.0300161	1.7397861	0	6.4112499	0	1.3484551
9.1530877	22.571566	13.149415	17.764407	11.330177	5.2193583	3.518538	14.425312	3.3177058	5.3938205
0	6.1558817	2.0762234	2.4787545	1.8025281	0	1.759269	0	0	0.3852729
18.306175	32.831369	16.955825	33.05006	23.175361	19.137647	12.314883	9.6168749	12.667604	17.33728
4.5765438	6.1558817	0	0	3.3475522	0	3.518538	0	0	1.733728
18.306175	32.831369	16.955825	33.05006	23.175361	19.137647	12.314883	9.6168749	12.667604	17.33728

0	2.0519606	0	0	0	0	0	0	0	0
18.306175	32.831369	16.955825	33.05006	23.175361	19.137647	12.314883	9.6168749	12.667604	17.33728
16.017903	8.2078423	15.917713	35.115689	29.355458	19.137647	15.833421	35.261875	14.477262	18.107826
219.6741	215.45586	688.96014	703.14002	747.27665	384.49273	406.39114	378.26375	447.28707	312.26368
6.8648158	20.519606	11.419229	14.046275	12.102689	27.836578	14.074152	17.630937	9.6515079	11.750823
219.6741	219.55978	691.03636	705.20565	749.33668	384.49273	404.63187	378.26375	448.79512	313.03422
16.017903	8.2078423	15.225638	35.115689	29.355458	19.137647	15.833421	35.261875	14.477262	18.107826
219.6741	215.45586	689.65221	703.14002	747.27665	384.49273	406.39114	378.26375	447.28707	312.26368
16.017903	8.2078423	15.225638	35.115689	29.355458	19.137647	15.833421	35.261875	14.477262	18.107826
219.6741	219.55978	691.03636	705.20565	749.33668	384.49273	404.63187	378.26375	448.79512	313.03422
0	0	0	0	0	1.7397861	0	0	0	0
20.594447	18.467645	11.765266	8.262515	12.360193	5.2193583	10.555614	4.8084375	3.3177058	3.6600925
2.2882719	6.1558817	17.993936	15.285653	12.875201	8.6989305	5.2778071	17.630937	10.254727	9.6318222
16.017903	18.467645	34.603724	26.440048	22.917857	19.137647	29.907573	52.892812	31.66901	31.785013
4.5765438	18.467645	3.8064096	2.0656287	5.1500803	1.7397861	1.759269	3.205625	2.7144866	4.2380018
**112.12532**	**143.63724**	**173.36466**	**142.52838**	**143.94474**	**173.97861**	**200.55667**	**225.99656**	**160.45632**	**117.50823**
2.2882719	6.1558817	17.993936	15.285653	12.875201	8.6989305	5.2778071	17.630937	10.254727	9.6318222
16.017903	18.467645	34.603724	26.440048	22.917857	19.137647	29.907573	52.892812	31.66901	31.785013
4.5765438	18.467645	3.8064096	2.0656287	5.1500803	1.7397861	1.759269	3.205625	2.7144866	4.2380018
**112.12532**	**143.63724**	**173.36466**	**142.52838**	**143.94474**	**173.97861**	**200.55667**	**225.99656**	**160.45632**	**117.50823**
25.170991	12.311763	68.169336	55.35885	75.70618	92.208664	59.815147	48.084375	38.907641	39.875744
0	0	0.3460372	0	2.3175361	1.7397861	1.759269	8.0140624	1.5080481	0.7705458
0	0	7.266782	4.957509	3.0900482	8.6989305	8.7963451	6.4112499	3.9209251	1.9263644
0	0	0.3460372	0	0.257504	0	0	0	0	0
25.170991	12.311763	68.169336	55.35885	75.70618	92.208664	59.815147	48.084375	38.907641	39.875744
9.1530877	10.259803	12.803378	1.652503	33.218018	17.397861	12.314883	8.0140624	16.28692	13.677188
0	0	0.6920745	0	0.772512	0	0	0	0.6032192	0
9.1530877	10.259803	12.803378	1.652503	33.218018	17.397861	12.314883	8.0140624	16.28692	13.677188
25.170991	12.311763	66.439149	53.706347	72.873636	86.989305	54.53734	48.084375	37.399593	38.912562
9.1530877	10.259803	12.803378	1.652503	33.218018	17.397861	12.314883	8.0140624	16.28692	13.677188
25.170991	12.311763	68.169336	55.35885	75.70618	92.208664	59.815147	48.084375	38.907641	39.875744
0	0	0.3460372	0	0	0	0	0	0	0
25.170991	12.311763	66.439149	53.706347	72.873636	86.989305	54.53734	48.084375	37.399593	38.912562
0	0	0	0	0	0	0	0	0	0
0	0	7.266782	4.957509	3.6050562	8.6989305	8.7963451	6.4112499	4.5241443	2.8895467
0	0	0.3460372	0	0	0	0	0	0	0
25.170991	12.311763	66.439149	53.706347	72.873636	86.989305	54.53734	48.084375	37.399593	38.912562
9.1530877	10.259803	12.803378	1.652503	33.218018	17.397861	12.314883	8.0140624	16.28692	13.677188
25.170991	12.311763	68.169336	55.35885	75.70618	92.208664	59.815147	48.084375	38.907641	39.875744
9.1530877	10.259803	12.803378	1.652503	33.218018	17.397861	12.314883	8.0140624	16.28692	13.677188
25.170991	12.311763	68.169336	55.35885	75.70618	92.208664	59.815147	48.084375	38.907641	39.875744
0	0	0	0	0	0	0	0	0	0.1926364
338.66424	219.55978	69.553485	68.578874	610.02701	180.93776	197.03813	168.29531	421.65025	186.08681
3544.5332	4136.7525	8548.1579	9063.9789	6670.1265	4869.6613	4647.9887	5789.3587	5639.7983	3582.8452
0	2.0519606	14.187527	8.6756407	7.2101124	3.4795722	1.759269	1.6028125	4.2225347	5.201184
2478.1985	2837.8615	6519.6876	7142.1179	5293.2525	3517.8475	3511.501	3986.1947	5319.1873	4683.3772
0	0	0	0	0	0	0	0	0	0

942.76803	1179.8773	2162.3867	1805.7726	1826.7335	835.09733	855.00474	886.35531	1509.8578	978.20787
0	0	0	0	0	0	0	0	0	0
942.76803	1179.8773	2162.3867	1805.7726	1826.7335	835.09733	855.00474	886.35531	1509.8578	978.20787
338.66424	219.55978	69.553485	68.578874	610.02701	180.93776	197.03813	168.29531	421.65025	186.08681
8443.7234	7945.1913	19181.536	22335.23	19204.907	7971.6999	9276.6255	9025.4371	19933.681	13117.579
0	2.0519606	14.187527	8.6756407	7.2101124	3.4795722	1.759269	1.6028125	4.2225347	5.201184
2478.1985	2837.8615	6519.6876	7142.1179	5293.2525	3517.8475	3511.501	3986.1947	5319.1873	4683.3772
**768.85937**	**707.9264**	**408.66998**	**350.74376**	**242.05377**	**107.86674**	**117.87102**	**137.84187**	**205.69776**	**358.68906**
418.75376	396.02839	584.11086	528.38783	590.45671	248.78941	246.29766	261.25844	475.93998	341.73705
16.017903	6.1558817	8.3048937	4.957509	5.6650883	1.7397861	8.7963451	0	3.9209251	2.8895467
**416.46549**	**398.08035**	**583.76482**	**528.80096**	**590.1992**	**248.78941**	**244.53839**	**261.25844**	**475.93998**	**341.73705**
4.5765438	8.2078423	38.064096	31.810683	36.050562	1.7397861	3.518538	4.8084375	32.272229	39.490471
0	0	0	0	0	0	0	0	0	0
**8873.9185**	**7093.6277**	**24581.793**	**16762.99**	**14119.975**	**6973.0627**	**7262.2625**	**9283.4899**	**18246.779**	**19410.048**
0	0	39.794282	23.961293	29.870466	40.01508	51.018801	41.673125	23.52555	43.150564
**8873.9185**	**7093.6277**	**24581.793**	**16762.99**	**14119.975**	**6973.0627**	**7262.2625**	**9283.4899**	**18246.779**	**19410.048**
0	0	4.1524468	2.0656287	1.2875201	5.2193583	3.518538	4.8084375	0.9048289	2.8895467
61.783342	65.662738	82.7029	59.076982	62.058468	60.892514	36.944649	48.084375	46.749491	60.295207
0	4.1039211	1.3841489	0.4131257	1.8025281	1.7397861	0	1.6028125	1.8096577	1.9263644
**121.27841**	**123.11763**	**70.591596**	**45.856958**	**153.47239**	**142.66246**	**91.481989**	**68.920937**	**130.89858**	**52.975022**
4.5765438	2.0519606	0	0.4131257	5.9225923	5.2193583	0	3.205625	3.9209251	1.1558187
2.2882719	4.1039211	2.7682979	3.305006	3.6050562	5.2193583	3.518538	3.205625	5.7305828	3.0821831
0	0	0	0	0	0	0	0	0	0
9.1530877	14.363724	12.111303	12.393772	23.690369	19.137647	10.555614	14.425312	10.556337	15.218279
0	0	1.3841489	0	0.772512	0	0	0	0	0
2.2882719	12.311763	9.6890426	8.262515	24.720385	3.4795722	31.666842	9.6168749	15.080481	5.9717298
0	0	0	0	0.257504	0	0	0	1.2064385	0
**123.56668**	**123.11763**	**70.937634**	**47.922587**	**153.7299**	**144.40225**	**91.481989**	**68.920937**	**132.70823**	**53.938205**
4.5765438	2.0519606	0	0.4131257	5.9225923	5.2193583	0	3.205625	3.9209251	1.1558187
0	0	0	0.4131257	0.257504	0	0	1.6028125	0.3016096	0
0	0	1.7301862	0.4131257	3.0900482	0	0	4.8084375	2.7144866	1.9263644
9.1530877	14.363724	12.111303	12.393772	23.690369	19.137647	10.555614	14.425312	10.556337	15.218279
0	0	1.3841489	0	0.772512	0	0	0	0	0
2.2882719	12.311763	9.6890426	8.262515	24.720385	3.4795722	31.666842	9.6168749	15.080481	5.9717298
0	0	0	0	0	0	0	0	0	0
1022.8575	993.14891	1663.055	1579.3797	1625.1078	1035.1727	995.74626	1509.8494	1750.2406	1110.1638
2.2882719	2.0519606	1.3841489	0	0.515008	1.7397861	0	3.205625	1.8096577	1.3484551
1194.4779	1025.9803	3831.6703	3005.4898	3121.2062	1769.3625	1592.1385	1490.6156	1524.335	1473.2835
1025.1458	970.57735	1989.022	1607.0592	1081.2594	798.56182	723.05957	735.69093	1007.6777	889.5951
0	0	1.0381117	0	1.0300161	0	0	1.6028125	0.9048289	0.3852729
0	0	1.7301862	0.4131257	0.515008	1.7397861	1.759269	1.6028125	1.2064385	0.7705458
2.2882719	0	5.5365958	7.4362635	5.1500803	3.4795722	1.759269	8.0140624	1.5080481	2.1190009
61.783342	77.974502	81.318751	111.95708	87.808869	107.86674	56.296609	123.41656	38.606032	64.918482
4.5765438	2.0519606	2.0762234	0.4131257	11.330177	10.438717	3.518538	4.8084375	9.3498983	6.3570027
2.2882719	2.0519606	19.032048	18.177533	16.995265	6.9591444	17.59269	16.028125	14.175652	13.484551
0	0	0	0	0.257504	0	0	1.6028125	0	0
61.783342	77.974502	80.280639	111.13083	87.551365	107.86674	56.296609	123.41656	38.606032	64.725845

4.5765438	6.1558817	13.841489	11.154395	16.737761	8.6989305	21.111228	33.659062	9.6515079	14.06246
2.2882719	2.0519606	19.032048	18.177533	16.995265	6.9591444	17.59269	16.028125	14.175652	13.484551
0	0	11.419229	7.0231377	56.908387	24.357006	12.314883	14.425312	29.557743	44.499019
2.2882719	2.0519606	16.955825	21.069413	21.115329	13.918289	5.2778071	8.0140624	11.762775	14.833006
0	0	0	0	0	0	0	0	0	0
9.1530877	0	2.4222607	2.0656287	1.8025281	3.4795722	0	3.205625	1.2064385	1.1558187
1709.3391	1142.942	880.66477	782.87329	949.4173	1205.6718	1169.9139	1075.4872	653.28644	435.16573
3160.1035	2263.3125	1490.0363	1068.3432	1734.8045	3757.938	4653.2665	4148.0787	1138.5763	1263.6951
**798.6069**	**670.99111**	**897.96663**	**1054.2969**	**1482.4506**	**598.48642**	**622.78123**	**538.545**	**1121.083**	**583.49579**
13.729632	4.1039211	12.111303	18.590659	11.330177	3.4795722	1.759269	3.205625	15.080481	5.201184
675.04022	539.66563	1304.2143	1488.4921	2061.5771	781.16396	730.09664	732.48531	1506.8417	909.62929
59.49507	69.766659	39.794282	43.378204	113.55927	38.275294	42.222456	49.687187	107.37303	33.904014
27.459263	18.467645	23.530532	12.393772	47.895747	24.357006	36.944649	22.439375	48.860759	32.362923
0	0	0	0	0	0	0	0	0	0
0	0	6.2286703	4.1312575	11.587681	5.2193583	7.0370761	8.0140624	10.254727	8.0907307
0	0	0	0	0	0	0	0	0	0
6.8648158	18.467645	6.9207447	7.4362635	5.6650883	3.4795722	1.759269	6.4112499	2.412877	6.1643662
9.1530877	14.363724	10.381117	11.980647	7.2101124	3.4795722	0	4.8084375	8.4450694	5.201184
0	0	0	0.8262515	0.257504	0	0	0	0	0.5779093
6.8648158	0	1.0381117	2.4787545	1.5450241	5.2193583	0	1.6028125	1.5080481	0.9631822
27.459263	16.415685	20.07016	16.52503	18.025281	5.2193583	19.351959	9.6168749	17.191748	14.447733
1045.7403	785.9009	2168.2693	1994.9842	1588.7998	831.61776	659.72588	663.56437	1277.6184	814.08162
0	0	2.4222607	0.8262515	4.6350723	5.2193583	1.759269	4.8084375	0.9048289	1.3484551
713.94084	531.45779	1341.2403	1424.4576	1301.9403	664.59829	629.81831	644.33062	899.39989	597.94353
20.594447	4.1039211	26.644867	30.571305	24.205377	8.6989305	15.833421	9.6168749	20.509454	17.915189
713.94084	531.45779	1341.2403	1424.4576	1301.9403	664.59829	629.81831	644.33062	899.39989	597.94353
4.5765438	6.1558817	15.225638	9.0887665	11.072673	0	12.314883	9.6168749	12.365994	11.94346
5230.9896	3711.9967	8469.2614	8003.072	6303.9558	3376.9248	2957.3312	2861.0203	5612.9551	4196.1997
118.99014	123.11763	114.53833	127.65586	202.91316	118.30546	121.38956	136.23906	207.20581	244.64829
36.612351	45.143132	82.356862	57.837605	93.216453	48.714011	51.018801	46.481562	78.116892	102.28995
0	0	5.5365958	3.305006	12.360193	5.2193583	10.555614	1.6028125	10.254727	5.201184
526.30254	391.92447	1065.1026	1314.9793	1313.2705	673.29722	636.85538	567.39562	866.52444	733.36695
572.06798	551.97739	634.63229	634.97428	835.34302	521.93583	511.94728	477.63812	687.97155	546.70223
414.17722	402.18427	945.37373	891.11224	1081.7744	1127.3814	1154.0805	1139.5997	886.73229	910.01457
572.06798	551.97739	634.63229	634.97428	835.34302	521.93583	511.94728	477.63812	687.97155	546.70223
**414.17722**	**402.18427**	**945.37373**	**891.11224**	**1081.7744**	**1127.3814**	**1154.0805**	**1139.5997**	**886.73229**	**910.01457**
16.017903	24.623527	32.181463	28.092551	29.097954	64.372086	42.222456	67.318124	24.12877	117.12296
0	0	0	0	0	0	0	0	0	0
**2583.459**	**1955.5184**	**7680.2965**	**8890.053**	**7850.0099**	**2571.4039**	**2658.2555**	**3024.5072**	**7609.0075**	**5374.9421**
13.729632	16.415685	22.49242	28.505677	37.85309	8.6989305	28.148304	14.425312	38.304422	18.878372
0	0	0	0	0	0	0	0	0	0
0	0	2.0762234	2.8918802	5.1500803	5.2193583	0	3.205625	2.1112673	2.3116373
0	0	0	0	0	0	0	0	0	0
2.2882719	2.0519606	11.419229	11.567521	15.707745	1.7397861	1.759269	6.4112499	7.5402405	8.0907307
**196.79139**	**194.93625**	**505.5604**	**388.3382**	**611.31453**	**358.39594**	**360.65015**	**392.68906**	**652.08**	**491.99348**
0	0	1.0381117	0.4131257	1.0300161	0	0	0	0.6032192	0.1926364
**118.99014**	**158.00096**	**171.98051**	**156.98778**	**184.63038**	**269.66685**	**195.27886**	**251.64156**	**76.608844**	**210.16636**

0	0	0	0	0	0	0	0	0	0
0	0	0	0	0	0	0	0	0	0
2.2882719	6.1558817	4.4984841	3.305006	7.4676164	3.4795722	1.759269	1.6028125	4.8257539	3.6600925
4.5765438	4.1039211	5.5365958	4.5443832	1.2875201	5.2193583	7.0370761	0	0.9048289	4.0453653
6.8648158	0	1.0381117	1.652503	1.2875201	0	5.2778071	3.205625	0.3016096	0.7705458
0	0	0	0	0	0	0	0	0	0
27.459263	16.415685	1.7301862	1.2393772	0	0	0	0	0.6032192	0.3852729
6.8648158	12.311763	11.765266	14.046275	16.480257	5.2193583	8.7963451	12.8225	21.414283	15.796188
0	0	0	0	0	0	0	0	0	0.1926364
6.8648158	12.311763	11.765266	14.046275	16.480257	5.2193583	8.7963451	12.8225	21.414283	15.603552
0	0	0	0.4131257	0	0	0	0	0	0
**135.00804**	**96.442147**	**77.166304**	**71.470755**	**101.19908**	**62.6323**	**54.53734**	**46.481562**	**75.100796**	**54.323477**
0	0	0	2.0656287	0	0	0	0	0.3016096	0.5779093
0	0	0	0	0	0	0	0	0	0
0	0	20.416197	16.111904	31.672994	12.178503	1.759269	6.4112499	26.541647	23.694283
0	0	0	0	0	0	0	0	0	0
0	0	20.416197	16.111904	31.672994	12.178503	1.759269	6.4112499	26.541647	23.694283
0	0	0	0	0	0	0	0	0	0
0	0	20.416197	16.111904	31.672994	12.178503	1.759269	6.4112499	26.541647	23.694283
0	4.1039211	1.0381117	0.8262515	2.0600321	0	7.0370761	1.6028125	1.5080481	1.5410916
**70.93643**	**71.81862**	**193.08878**	**191.27722**	**172.01268**	**107.86674**	**140.74152**	**110.59406**	**142.96296**	**68.000665**
0	0	0	0	0	0	0	0	0	0.1926364
6.8648158	4.1039211	2.4222607	3.7181317	2.8325442	3.4795722	1.759269	1.6028125	3.6193155	1.1558187
0	0	0	0.4131257	0	0	0	0	0	0.3852729
0	0	13.841489	9.0887665	10.815169	5.2193583	0	1.6028125	6.0321924	6.934912
0	0	0.3460372	0	0.515008	0	0	0	0.9048289	0.3852729
0	0	12.457341	9.0887665	8.7551365	3.4795722	3.518538	8.0140624	9.9531175	7.3201849
0	0	0.3460372	0	0.515008	0	0	0	0.9048289	0.3852729
0	0	12.457341	9.0887665	8.7551365	3.4795722	3.518538	8.0140624	9.9531175	7.3201849
0	0	0.3460372	0	0.515008	0	0	0	0.9048289	0.3852729
0	0	12.457341	9.0887665	8.7551365	3.4795722	3.518538	8.0140624	9.9531175	7.3201849
0	0	0.3460372	0	0.515008	0	0	0	0.9048289	0.3852729
0	0	12.457341	9.0887665	8.7551365	3.4795722	3.518538	8.0140624	9.9531175	7.3201849
0	0	0.3460372	0	0.515008	0	0	0	0.9048289	0.3852729
0	0	12.457341	9.0887665	8.7551365	3.4795722	3.518538	8.0140624	9.9531175	7.3201849
0	0	0.3460372	0	0.515008	0	0	0	0.9048289	0.3852729
0	0	12.457341	9.0887665	8.7551365	3.4795722	3.518538	8.0140624	9.9531175	7.3201849
**6.8648158**	**4.1039211**	**35.641835**	**30.984431**	**32.188002**	**12.178503**	**12.314883**	**33.659062**	**34.685106**	**37.178834**
0	0	0	0	0	0	0	0	0	0
0	2.0519606	0	0	0	0	0	0	0	0
2.2882719	0	0	0	0	0	0	0	0	0
0	0	0	0	0	0	0	0	0	0.1926364
0	0	4.8445213	4.5443832	9.2701445	6.9591444	1.759269	9.6168749	6.333802	5.201184
0	0	0	0.4131257	0.257504	1.7397861	1.759269	0	0.3016096	0
0	4.1039211	1.0381117	6.610012	3.3475522	0	1.759269	3.205625	2.412877	2.3116373

4.5765438	14.363724	7.9588564	8.262515	9.7851526	3.4795722	1.759269	8.0140624	8.4450694	9.6318222
0	0	0.3460372	0	0	0	0	0	0	0
0	0	0	0	0	0	0	0	0	0
0	0	1.0381117	0.4131257	1.5450241	0	1.759269	0	2.1112673	2.1190009
4.5765438	8.2078423	3.4603724	4.957509	4.8925763	5.2193583	1.759269	4.8084375	5.1273636	4.4306382
0	0	0.3460372	1.2393772	0.515008	1.7397861	0	3.205625	0.6032192	0.5779093
0	0	1.0381117	1.652503	0.515008	0	1.759269	0	0.9048289	1.1558187
0	0	0	0	0	0	0	0	0	0
0	0	1.0381117	1.652503	0.515008	0	1.759269	0	0.9048289	1.1558187
0	0	0	0	0	0	0	0	0	0
0	0	0.3460372	0	0	0	0	0	0	0
**43.477166**	**55.402935**	**44.638804**	**50.814467**	**64.1185**	**31.31615**	**12.314883**	**32.05625**	**81.434598**	**39.875744**
0	0	1.3841489	0.4131257	1.2875201	0	0	1.6028125	1.8096577	0.3852729
0	2.0519606	1.3841489	1.652503	1.5450241	3.4795722	0	0	1.5080481	0.5779093
0	0	0	0	0	0	0	0	0	0
0	0	0	0	0	0	0	0	0	0
0	0	0.6920745	0	1.5450241	1.7397861	0	0	0.9048289	0.3852729
0	0	18.686011	6.610012	18.797793	5.2193583	0	8.0140624	11.461166	7.7054578
0	0	0	3.305006	2.0600321	0	0	1.6028125	1.2064385	2.1190009
0	2.0519606	14.879601	11.154395	11.845185	5.2193583	8.7963451	8.0140624	10.556337	9.2465494
**22.882719**	**22.571566**	**4.4984841**	**1.2393772**	**1.8025281**	**0**	**0**	**1.6028125**	**2.1112673**	**1.5410916**
0	0	0	0	0	0	0	0	0	0
**27.459263**	**41.039211**	**813.53354**	**835.34026**	**809.33512**	**447.12503**	**358.89088**	**431.15656**	**625.83996**	**409.35245**
0	0	0.3460372	0	0	0	0	0	0	0.1926364
0	0	0	0	0	0	0	0	0	0
0	0	0	0	0	0	0	0	0.3016096	0
0	0	0	0	0	0	0	0	0	0
0	2.0519606	5.1905586	2.0656287	3.3475522	0	0	0	1.8096577	3.467456
0	0	0	0	0	0	0	0	0	0
0	2.0519606	5.1905586	2.0656287	3.3475522	0	0	0	1.8096577	3.467456
0	0	0	0	0	0	0	0	0	0.1926364
0	0	0	0	0	0	0	0	0	0
0	0	0	0	0.515008	0	0	0	0	0.5779093
0	0	0	0	0.257504	0	0	0	0	0
0	4.1039211	8.6509309	7.0231377	6.6951044	5.2193583	1.759269	1.6028125	3.9209251	4.4306382
0	0	0	0	0	0	0	0	0	0
0	2.0519606	3.1143351	5.7837605	6.6951044	0	0	0	6.9370213	2.1190009
6.8648158	2.0519606	2.0762234	0.8262515	8.2401285	0	3.518538	1.6028125	4.8257539	1.5410916
0	0	1.0381117	0	1.5450241	0	1.759269	0	0.6032192	0.7705458
4.5765438	0	1.0381117	0.4131257	1.8025281	1.7397861	1.759269	1.6028125	1.2064385	0.5779093
0	0	1.0381117	0	1.5450241	0	1.759269	0	0.6032192	0.7705458
4.5765438	0	1.0381117	0.4131257	1.8025281	1.7397861	1.759269	1.6028125	1.2064385	0.5779093
0	0	1.0381117	0	1.5450241	0	1.759269	0	0.6032192	0.7705458
4.5765438	0	1.0381117	0.4131257	1.8025281	1.7397861	1.759269	1.6028125	1.2064385	0.5779093
0	0	1.0381117	0	1.5450241	0	1.759269	0	0.6032192	0.7705458
4.5765438	0	1.0381117	0.4131257	1.8025281	1.7397861	1.759269	1.6028125	1.2064385	0.5779093
0	0	1.7301862	0.8262515	1.0300161	1.7397861	0	3.205625	1.2064385	2.8895467

0	0	0	0	0	0	0	0	0	0.1926364
0	0	0.6920745	0.4131257	0.515008	0	0	0	0.6032192	0.5779093
0	0	0	0	0	0	0	0	0	0
4.5765438	6.1558817	3.4603724	2.4787545	3.0900482	0	1.759269	4.8084375	0.9048289	1.733728
0	2.0519606	0.3460372	0.8262515	3.0900482	0	0	1.6028125	0.3016096	1.3484551
**18.306175**	**26.675487**	**44.638804**	**42.138826**	**44.548195**	**27.836578**	**17.59269**	**32.05625**	**57.004218**	**67.808029**
2.2882719	0	1.7301862	3.305006	0.257504	0	0	3.205625	1.2064385	1.9263644
0	0	0	0	0	0	0	0	0	0
0	0	4.4984841	7.8493892	2.3175361	1.7397861	0	3.205625	2.412877	3.8527289
0	2.0519606	1.3841489	0	0.257504	1.7397861	0	0	1.8096577	0
0	0	10.03508	9.0887665	7.9826245	3.4795722	8.7963451	9.6168749	7.8418502	4.4306382

164_R_CK_r	164_R_Pu_i	164_R_Pu_i	164_R_Pu_i	58_R_CK_re	58_R_CK_re	58_R_CK_re	58_R_Pu_in	58_R_Pu_in	58_R_Pu_in
2.4612	3.9034406	0	3.2448869	0.4106411	0.8504772	0.826135	0	0	0
0	0	0	0	0	0	0	0	0	0
380.11867	183.46171	160.06994	154.13213	578.59332	474.56627	423.80728	198.88262	176.81825	214.8363
23.7916	9.7586015	7.0360412	6.4897738	18.47885	25.514315	9.9136206	13.38633	9.6886715	7.3448308
0	0	0	0	0	0	0	0	0	0
50.0444	39.034406	42.216247	34.071313	222.97812	260.67126	232.14395	101.35364	111.41972	108.33625
0	0	0	0	0	0	0	0	0	0
0.5469333	0	0	0	4.9276933	6.8038175	8.2613505	1.9123329	2.4221679	1.8362077
0	0	0	0	0	0	0	0	0	0
0.5469333	0	0	0	4.9276933	6.8038175	8.2613505	1.9123329	2.4221679	1.8362077
37.464933	27.324084	49.252289	30.826426	54.204626	79.944855	43.37209	38.246658	33.91035	60.594854
0.5469333	0	1.7590103	0	1.6425644	1.2757158	1.6522701	1.9123329	0	0
7.6570667	7.8068812	5.2770309	3.2448869	1.2319233	1.2757158	2.0653376	0	2.4221679	3.6724154
0	0	0	0	0	0	0	0	0	0
23.244667	7.8068812	14.072082	9.7346607	1387.5563	1616.7571	1313.1417	694.17685	615.23064	530.66403
0	0	0	0	3.2851289	2.9766701	4.9568103	0	7.2665036	3.6724154
20.51	9.7586015	3.5180206	3.2448869	14.372439	21.26193	20.240309	7.6493317	7.2665036	3.6724154
0	0	0	0	0	0	0	0	0	0
0	0	0	0	0	0	0	0	0	0
5.1958667	3.9034406	1.7590103	0	4.5170522	6.3785789	6.6090804	0	0	0
26590.258	10240.676	13009.64	10748.688	25569.39	31524.212	22717.475	13363.382	11645.783	12438.471
12.852933	9.7586015	10.554062	17.846878	25.049108	35.294803	20.653376	13.38633	9.6886715	27.543116
1071.4424	484.02663	423.92148	452.66172	938.72557	1034.1803	781.93682	447.4859	440.83455	422.32777
0	0	0	0	0	0	0	0	0	0
6.2897333	3.9034406	14.072082	6.4897738	2.4638466	2.9766701	2.0653376	1.9123329	7.2665036	5.5086231
0	0	0	0	0	0	0	0	0	0
0.2734667	0	0	0	0.4106411	0	0	0	0	1.8362077
3.5550667	1.9517203	3.5180206	1.6224435	13.551157	12.757158	14.044296	1.9123329	2.4221679	0
16.681467	21.468923	29.903175	11.357104	17.657568	23.388122	25.197119	19.123329	29.066014	22.034492
0	0	0	0	0	0	0	0	0	0
77.664533	68.31021	47.493278	50.295747	146.18823	187.53022	124.33332	110.91531	87.198043	77.120724
334.7232	316.17869	170.624	222.27475	1011.409	748.84516	931.88033	489.55723	486.85574	471.90538
0.2734667	0	0	0	0	0.4252386	0.4130675	0	0	0
2.4612	1.9517203	0	0	7.3915399	5.1028631	6.1960128	1.9123329	2.4221679	3.6724154
27.346667	68.31021	47.493278	30.826426	7.802181	19.560975	17.761904	42.071324	29.066014	53.250023
976.82293	1405.2386	724.71225	1114.6187	1017.9793	1269.3372	1132.6311	1000.1501	607.96414	756.51757
61.256533	97.586015	93.227546	48.673304	63.238731	115.6649	75.178289	116.65231	108.99755	286.4484
1191.4943	1325.2181	934.03447	1052.9658	2002.6967	2011.8038	1827.8238	1701.9763	1642.2298	1920.6733
77.664533	68.31021	47.493278	50.295747	146.18823	187.53022	124.33332	110.91531	87.198043	77.120724
335.81707	320.08213	170.624	223.8972	1017.5687	756.49945	940.14168	491.46956	489.27791	471.90538
2489.0936	1104.6737	1447.6655	1297.9548	2797.6979	3110.6203	2419.7496	1091.9421	959.17848	1004.4056
184.86347	185.41343	103.78161	121.68326	597.89345	427.79002	605.14392	328.92126	230.10595	266.25012
15.861067	21.468923	28.144165	9.7346607	16.015003	22.112407	21.892579	17.210996	24.221679	20.198285
184.86347	185.41343	103.78161	121.68326	597.89345	427.79002	605.14392	328.92126	230.10595	266.25012
27.346667	68.31021	47.493278	30.826426	7.802181	19.560975	17.761904	42.071324	29.066014	53.250023
976.82293	1405.2386	724.71225	1114.6187	1017.9793	1269.3372	1132.6311	1000.1501	607.96414	756.51757

61.803467	99.537735	93.227546	48.673304	63.238731	115.6649	75.178289	116.65231	108.99755	286.4484
90.790933	140.52386	68.601402	97.346607	124.42426	240.2598	132.59468	89.879647	94.464547	110.17246
0.8204	3.9034406	1.7590103	0	3.2851289	6.3785789	4.9568103	13.38633	14.533007	3.6724154
2.4612	1.9517203	0	0	7.3915399	5.1028631	6.1960128	1.9123329	2.4221679	3.6724154
0	0	0	0	0	0	0	0	0	0
4.6489333	1.9517203	1.7590103	0	12.319233	14.883351	12.805093	7.6493317	9.6886715	12.853454
61.256533	97.586015	93.227546	48.673304	63.238731	115.6649	75.178289	116.65231	108.99755	286.4484
1191.4943	1325.2181	934.03447	1052.9658	2002.6967	2011.8038	1827.8238	1701.9763	1642.2298	1920.6733
77.664533	68.31021	47.493278	50.295747	146.18823	187.53022	124.33332	110.91531	87.198043	77.120724
334.7232	316.17869	170.624	222.27475	1011.409	748.84516	931.88033	489.55723	486.85574	471.90538
218.49987	216.64095	126.64874	128.17303	875.89748	980.17495	1047.9523	493.38189	484.43357	519.64678
184.86347	185.41343	103.78161	121.68326	597.89345	427.79002	605.14392	328.92126	230.10595	266.25012
218.49987	216.64095	126.64874	126.55059	872.20171	978.89924	1043.4086	489.55723	482.01141	517.81057
184.86347	185.41343	103.78161	121.68326	597.89345	427.79002	605.14392	328.92126	230.10595	266.25012
0	0	0	0	0.4106411	0.4252386	1.2392026	0	2.4221679	0
334.7232	316.17869	170.624	222.27475	1011.409	748.84516	931.88033	489.55723	486.85574	471.90538
0	0	0	0	0.4106411	0.4252386	1.2392026	0	2.4221679	0
334.7232	316.17869	170.624	222.27475	1011.409	748.84516	931.88033	489.55723	486.85574	471.90538
5.4693333	5.8551609	1.7590103	6.4897738	9.4447455	8.0795332	8.2613505	0	0	3.6724154
334.7232	316.17869	170.624	222.27475	1011.409	748.84516	931.88033	489.55723	486.85574	471.90538
3.8285333	5.8551609	3.5180206	3.2448869	18.889491	23.388122	20.653376	7.6493317	7.2665036	11.017246
184.59	185.41343	103.78161	121.68326	598.30409	427.79002	605.14392	328.92126	230.10595	266.25012
3.5550667	1.9517203	0	1.6224435	11.497951	14.883351	11.978958	7.6493317	9.6886715	11.017246
0	0	0	0	0	0	0	0	0	0
20804.25	12266.562	9062.4211	8644.3787	25904.473	17792.833	23874.064	13277.327	12677.627	10486.582
176.11253	93.682574	38.698227	84.36706	360.54289	361.02756	230.07861	246.69095	193.77343	187.29319
63.1708	13.662042	5.2770309	8.1122173	92.394249	77.818662	55.351048	13.38633	14.533007	22.034492
0	0	0	0	0	0	0	0	0	0
20804.25	12266.562	9062.4211	8644.3787	25904.473	17792.833	23874.064	13277.327	12677.627	10486.582
176.11253	93.682574	38.698227	84.36706	360.54289	361.02756	230.07861	246.69095	193.77343	187.29319
9.0244	5.8551609	10.554062	6.4897738	9.8553866	8.9300104	13.631228	3.8246658	7.2665036	9.1810385
181.58187	316.17869	299.03175	180.09122	337.95763	527.72109	387.45734	273.46361	312.45966	302.97427
26.2528	11.710322	14.072082	29.203982	24.227825	26.364793	19.414174	7.6493317	19.377343	20.198285
184.04307	314.22697	300.79076	180.09122	343.29597	532.82395	392.41415	275.37594	310.03749	306.64669
17.2284	9.7586015	5.2770309	16.224435	16.836285	17.860021	16.935768	11.473998	14.533007	20.198285
181.58187	316.17869	299.03175	180.09122	337.95763	527.72109	387.45734	273.46361	312.45966	302.97427
12.852933	7.8068812	0	1.6224435	20.532055	25.089077	16.522701	11.473998	7.2665036	7.3448308
184.04307	314.22697	300.79076	180.09122	343.29597	532.82395	392.41415	275.37594	310.03749	306.64669
1264.5099	784.59156	860.15604	858.27259	1840.0828	2584.1749	1615.9202	965.72813	1114.1972	1076.0177
103.91733	76.117092	75.637443	61.652851	98.964507	76.968185	106.57142	66.931652	77.509372	62.431062
1264.5099	784.59156	860.15604	858.27259	1840.0828	2584.1749	1615.9202	965.72813	1114.1972	1076.0177
103.91733	76.117092	75.637443	61.652851	98.964507	76.968185	106.57142	66.931652	77.509372	62.431062
0	0	0	0	10.676669	4.2523859	11.565891	0	0	0
210.8428	152.23418	156.55192	124.92815	368.75571	343.16754	417.1982	219.91829	273.70497	247.88804
246.66693	171.75139	133.68478	118.43837	429.11996	361.87804	567.96784	256.25261	295.50448	266.25012
211.93667	152.23418	158.31093	124.92815	368.75571	342.7423	418.02433	219.91829	276.12714	247.88804
0	0	0	0	0.4106411	0.4252386	0	0	0	0

210.8428	152.23418	156.55192	124.92815	368.75571	343.16754	417.1982	219.91829	273.70497	247.88804
0.5469333	1.9517203	0	1.6224435	0.4106411	0.8504772	0.4130675	0	2.4221679	1.8362077
0.5469333	0	0	4.8673304	0.8212822	2.126193	0.4130675	0	0	0
6.2897333	3.9034406	5.2770309	0	8.2128221	8.9300104	8.674418	7.6493317	0	0
0.5469333	0	0	0	0	0	0	0	0	0
246.66693	171.75139	133.68478	118.43837	429.11996	361.87804	567.96784	256.25261	295.50448	266.25012
211.6632	152.23418	158.31093	124.92815	368.75571	342.7423	418.02433	219.91829	276.12714	247.88804
0	0	0	0	0.4106411	0.4252386	0	0	0	0
210.8428	152.23418	156.55192	124.92815	368.75571	343.16754	417.1982	219.91829	273.70497	247.88804
0.2734667	0	0	0	0.4106411	0.8504772	0.826135	0	0	0
4.3754667	3.9034406	8.7950516	6.4897738	15.604362	12.757158	15.696566	5.7369988	4.8443357	3.6724154
649735.2	253637.76	255434.68	215721.7	614365.91	535829.97	645724.5	293828.04	244607.47	233778.62
1178.6413	427.42674	404.57237	464.01883	4490.7711	3177.3828	3865.0728	1845.4013	1160.2184	1149.466
**649800.83**	**253784.14**	**255563.09**	**215869.35**	**615269.73**	**536662.16**	**646597.31**	**294218.16**	**244881.17**	**234021**
10.118267	1.9517203	7.0360412	8.1122173	11.497951	11.906681	14.044296	15.298663	24.221679	12.853454
649400.75	253756.82	255445.24	215721.7	615370.75	536666.84	646533.29	294208.6	244924.77	234009.98
52.232133	44.889567	42.216247	25.959095	91.572967	159.03923	59.894791	47.808323	58.132029	69.775893
649735.2	253637.76	255434.68	215721.7	614365.91	535829.97	645724.5	293828.04	244607.47	233778.62
681.20547	290.80632	277.92363	347.2029	3802.9473	2622.0212	3198.7949	1684.7653	978.55582	971.35387
649735.2	253637.76	255434.68	215721.7	614365.91	535829.97	645724.5	293828.04	244607.47	233778.62
1186.2984	429.37846	409.8494	464.01883	4501.8585	3191.4156	3875.3995	1847.3136	1167.4849	1158.6471
13455.927	9327.2713	6684.2392	6426.4985	25565.283	25357.402	24064.901	13172.149	12171.394	11579.126
1.3673333	0	0	1.6224435	2.8744878	5.9533403	2.8914727	1.9123329	2.4221679	0
**649800.83**	**253784.14**	**255563.09**	**215869.35**	**615269.73**	**536662.16**	**646597.31**	**294218.16**	**244881.17**	**234021**
1.0938667	0	0	0	8.2128221	6.3785789	10.326688	7.6493317	9.6886715	1.8362077
649821.07	253733.4	255515.6	215840.14	615186.78	536572.86	646536.59	294181.82	244835.15	233967.75
0	0	0	0	0.8212822	0	1.2392026	0	2.4221679	1.8362077
649821.07	253733.4	255515.6	215840.14	615186.78	536572.86	646536.59	294181.82	244835.15	233967.75
27.346667	17.565483	8.7950516	11.357104	73.915399	63.36055	50.394238	30.597327	16.955175	29.379323
649421.8	253706.07	255397.74	215694.12	615289.03	536580.09	646472.98	294172.26	244881.17	233956.73
0	0	0	0	0.8212822	0	1.2392026	0	2.4221679	1.8362077
8.7509333	9.7586015	1.7590103	8.1122173	14.78308	25.514315	19.001106	24.860328	9.6886715	25.706908
137.28027	333.74417	297.27274	277.43783	181.09273	298.09225	161.09633	286.84994	346.37001	317.66393
0	0	0	0	0	0.4252386	0	0	0	0
124.15387	322.03385	286.71868	261.2134	165.07773	276.40508	149.53044	277.28827	326.99266	301.13806
0.2734667	1.9517203	0	0	2.0532055	2.9766701	2.4784051	0	0	0
137.0068	333.74417	299.03175	277.43783	181.50337	298.94273	160.68327	286.84994	346.37001	319.50014
8.7509333	9.7586015	1.7590103	8.1122173	14.78308	25.514315	19.001106	24.860328	9.6886715	25.706908
137.28027	333.74417	297.27274	277.43783	181.09273	298.09225	161.09633	286.84994	346.37001	317.66393
3.0081333	0	5.2770309	1.6224435	4.9276933	3.4019087	1.2392026	0	2.4221679	5.5086231
124.15387	322.03385	286.71868	261.2134	165.07773	276.40508	149.53044	277.28827	326.99266	301.13806
8.7509333	9.7586015	1.7590103	8.1122173	14.78308	25.514315	19.001106	24.860328	9.6886715	25.706908
137.0068	333.74417	299.03175	277.43783	181.50337	298.94273	160.68327	286.84994	346.37001	319.50014
1.6408	3.9034406	1.7590103	1.6224435	6.5702577	3.4019087	2.8914727	1.9123329	2.4221679	5.5086231
44.575067	177.60655	186.45509	146.01991	54.615267	62.084834	42.545955	139.6003	208.30644	176.27594
1.6408	3.9034406	1.7590103	1.6224435	6.5702577	3.4019087	2.8914727	1.9123329	2.4221679	5.5086231
44.575067	177.60655	186.45509	146.01991	54.615267	62.084834	42.545955	139.6003	208.30644	176.27594

3131.7403	1092.9634	886.5412	911.81322	3747.5107	3725.0901	3684.5623	1177.9971	1450.8786	1421.2248
6079.7109	3778.5305	3196.1217	1888.5242	7557.4389	6695.3816	9006.1112	3361.8813	3538.7873	3079.3203
3131.7403	1092.9634	886.5412	911.81322	3747.5107	3725.0901	3684.5623	1177.9971	1450.8786	1421.2248
6079.7109	3778.5305	3196.1217	1888.5242	7557.4389	6695.3816	9006.1112	3361.8813	3538.7873	3079.3203
0	0	0	0	0	0.8504772	0	0	0	0
30.901733	25.372364	38.698227	42.18353	23.817184	33.593849	21.479511	21.035662	12.110839	25.706908
1.0938667	3.9034406	0	1.6224435	13.961798	8.5047718	10.326688	3.8246658	16.955175	9.1810385
30.3548	25.372364	38.698227	40.561086	22.585261	28.490986	19.001106	21.035662	12.110839	25.706908
0.8204	0	0	3.2448869	0.4106411	0.4252386	0.826135	0	0	0
0.5469333	0	1.7590103	3.2448869	0.4106411	1.7009544	2.4784051	0	0	0
4.102	25.372364	14.072082	27.581539	2.8744878	4.2523859	1.6522701	13.38633	7.2665036	11.017246
917.75413	495.73695	504.83596	833.93594	854.1335	1187.6914	760.04424	600.47254	663.674	784.06069
16.408	9.7586015	3.5180206	27.581539	0	0	0	0	0	0
30.901733	25.372364	38.698227	42.18353	23.817184	33.593849	21.892579	21.035662	12.110839	25.706908
0	0	0	0	0	0	0.826135	0	0	0
6.0162667	9.7586015	12.313072	4.8673304	11.08731	14.458112	2.4784051	9.5616646	4.8443357	9.1810385
5.1958667	0	3.5180206	1.6224435	4.9276933	2.9766701	4.9568103	3.8246658	7.2665036	7.3448308
30.901733	25.372364	38.698227	42.18353	23.817184	34.019087	21.479511	21.035662	12.110839	25.706908
1.3673333	0	0	1.6224435	0.8212822	2.126193	0.826135	0	2.4221679	1.8362077
30.3548	25.372364	38.698227	40.561086	22.585261	28.490986	19.001106	21.035662	12.110839	25.706908
5.1958667	0	3.5180206	1.6224435	4.9276933	2.9766701	4.9568103	3.8246658	7.2665036	7.3448308
30.901733	25.372364	38.698227	42.18353	23.817184	34.019087	21.479511	21.035662	12.110839	25.706908
2.1877333	3.9034406	7.0360412	3.2448869	2.4638466	2.9766701	1.6522701	3.8246658	7.2665036	3.6724154
6.0162667	9.7586015	12.313072	4.8673304	11.08731	14.458112	2.4784051	9.5616646	4.8443357	9.1810385
0.2734667	0	0	1.6224435	0	0.4252386	0	0	0	0
30.901733	25.372364	38.698227	42.18353	23.817184	33.593849	21.479511	21.035662	12.110839	25.706908
0	0	0	0	0	0	0.826135	0	0	0
6.0162667	9.7586015	12.313072	4.8673304	11.08731	14.458112	2.4784051	9.5616646	4.8443357	9.1810385
151.50053	113.19978	94.986557	121.68326	172.46927	270.45174	175.96676	126.21397	89.620211	167.0949
1.0938667	1.9517203	1.7590103	1.6224435	2.0532055	2.5514315	4.9568103	17.210996	9.6886715	12.853454
114.30907	87.827413	61.565361	69.765069	199.16094	198.16118	213.14284	65.019319	82.353708	67.939685
10.118267	13.662042	8.7950516	9.7346607	17.246927	33.593849	21.066444	7.6493317	16.955175	12.853454
71.648267	60.503329	26.385155	45.428417	39.421546	66.762459	28.501659	21.035662	36.332518	40.396569
46.215867	3.9034406	24.626144	40.561086	25.87039	32.318133	17.348836	11.473998	16.955175	27.543116
73.289067	56.599889	52.770309	48.673304	171.23734	302.34464	166.46621	70.756318	123.53056	71.6121
0	0	0	0	0	0	0	0	0	0
68.9136	60.503329	26.385155	43.805973	39.010905	65.061504	28.088592	21.035662	36.332518	38.560362
22.697733	5.8551609	3.5180206	0	13.551157	8.9300104	4.9568103	1.9123329	2.4221679	1.8362077
114.30907	87.827413	61.565361	69.765069	199.16094	198.16118	213.14284	65.019319	82.353708	67.939685
10.118267	13.662042	8.7950516	9.7346607	17.246927	33.593849	21.066444	7.6493317	16.955175	12.853454
73.289067	56.599889	52.770309	48.673304	171.64798	303.19512	167.29235	70.756318	123.53056	71.6121
0	0	0	0	0.4106411	0.4252386	0	0	0	0
2.7346667	3.9034406	5.2770309	8.1122173	2.0532055	6.8038175	1.2392026	5.7369988	9.6886715	3.6724154
0	0	0	0	0	0	0	0	0	0
0.2734667	1.9517203	0	3.2448869	0.4106411	0.8504772	1.2392026	0	0	1.8362077
3.0081333	0	1.7590103	3.2448869	4.5170522	2.126193	1.2392026	1.9123329	0	0
2.7346667	3.9034406	5.2770309	8.1122173	2.0532055	6.8038175	1.2392026	5.7369988	9.6886715	3.6724154

1.3673333	1.9517203	0	0	0	0.4252386	0.4130675	1.9123329	0	1.8362077
0.2734667	1.9517203	0	3.2448869	0.4106411	0.8504772	1.2392026	0	0	1.8362077
0	0	0	0	0	0	0	0	0	0
0.2734667	1.9517203	0	3.2448869	0.4106411	0.8504772	1.2392026	0	0	1.8362077
0	0	0	0	0	0	0	0	0	0
2.7346667	3.9034406	5.2770309	8.1122173	2.0532055	6.8038175	1.2392026	5.7369988	9.6886715	3.6724154
0	0	0	0	0	0	0	0	0	0
0.2734667	1.9517203	0	3.2448869	0.4106411	0.8504772	1.2392026	0	0	1.8362077
0	0	0	0	0	0	0	0	0	0
10.6652	3.9034406	8.7950516	3.2448869	6.1596166	7.229056	7.4352154	1.9123329	2.4221679	3.6724154
12.306	7.8068812	7.0360412	8.1122173	6.1596166	14.032873	6.1960128	3.8246658	4.8443357	7.3448308
12.306	7.8068812	7.0360412	8.1122173	6.1596166	14.032873	6.1960128	3.8246658	4.8443357	7.3448308
0	0	0	0	0	0	0	0	0	0
0	0	0	0	0	0	0	0	0	0
12.306	7.8068812	7.0360412	8.1122173	6.1596166	14.032873	6.1960128	3.8246658	4.8443357	7.3448308
3306.7589	2351.823	2274.4003	2143.2478	3908.8927	4438.6404	3894.4006	2191.5335	2424.59	2275.0613
13.1264	9.7586015	7.0360412	6.4897738	30.798083	28.490986	29.327794	11.473998	9.6886715	11.017246
3306.7589	2351.823	2274.4003	2143.2478	3908.8927	4438.6404	3894.4006	2191.5335	2424.59	2275.0613
10.118267	5.8551609	3.5180206	8.1122173	27.102313	17.860021	24.784051	11.473998	19.377343	5.5086231
38401.283	19037.08	17785.353	18478.009	44848.169	44907.747	40963.08	24363.121	22925.819	21419.363
96.8072	23.420644	15.831093	37.316199	98.553866	164.1421	99.962341	17.210996	50.865525	38.560362
712.9276	333.74417	327.17592	342.33557	721.49643	709.72321	591.92576	409.23925	380.28036	365.40533
12.579467	15.613762	5.2770309	8.1122173	16.015003	23.813361	21.892579	13.38633	4.8443357	5.5086231
122.2396	46.841287	42.216247	58.407964	129.35195	130.12301	128.05093	80.317983	62.976365	71.6121
0	0	0	0	0	0.4252386	0	0	0	0
1240.7183	579.66093	626.20767	610.03874	1441.7609	1502.3679	1313.1417	958.07879	644.29665	800.58656
1.9142667	1.9517203	3.5180206	0	1.6425644	2.9766701	0.826135	1.9123329	2.4221679	9.1810385
1233.3347	579.66093	620.93064	605.17141	1433.9587	1495.9894	1304.8803	952.3418	644.29665	798.75035
1.0938667	0	1.7590103	3.2448869	2.8744878	9.355249	3.7176077	1.9123329	2.4221679	1.8362077
38481.136	19072.211	17824.051	18499.1	44926.601	44972.808	41025.453	24397.543	22964.574	21448.742
16.681467	9.7586015	14.072082	8.1122173	31.619365	96.103922	25.197119	9.5616646	24.221679	36.724154
712.9276	333.74417	327.17592	342.33557	721.49643	709.72321	591.92576	409.23925	380.28036	365.40533
3.2816	3.9034406	1.7590103	3.2448869	6.5702577	5.5281017	3.7176077	7.6493317	0	11.017246
1240.7183	579.66093	626.20767	610.03874	1441.7609	1502.3679	1313.1417	958.07879	644.29665	802.42277
1.9142667	1.9517203	3.5180206	0	1.6425644	2.9766701	0.826135	1.9123329	2.4221679	9.1810385
0	0	0	0	0	0	0	0	0	0
656.59347	292.75804	290.2367	301.77448	664.00667	592.35736	524.59575	369.08025	348.79217	330.51739
1233.3347	579.66093	620.93064	605.17141	1433.9587	1495.9894	1304.8803	952.3418	644.29665	798.75035
3.2816	3.9034406	1.7590103	3.2448869	6.5702577	5.5281017	3.7176077	7.6493317	0	11.017246
656.59347	292.75804	290.2367	301.77448	664.00667	592.35736	524.59575	369.08025	348.79217	330.51739
0	0	0	0	0	0	0	0	0	0
0.8204	1.9517203	5.2770309	1.6224435	10.676669	7.6542946	6.1960128	1.9123329	4.8443357	11.017246
5.7428	3.9034406	5.2770309	1.6224435	66.9345	47.626722	52.046508	26.772661	9.6886715	40.396569
0.8204	3.9034406	0	3.2448869	11.908592	14.032873	18.588039	5.7369988	7.2665036	22.034492
14.220267	13.662042	22.867134	16.224435	156.04362	177.32449	114.00664	82.230316	70.242868	117.51729
0.2734667	0	1.7590103	6.4897738	4.9276933	5.9533403	12.805093	3.8246658	7.2665036	3.6724154
14.220267	13.662042	22.867134	16.224435	156.04362	177.32449	114.00664	82.230316	70.242868	117.51729

0	0	0	0	1.2319233	0.4252386	2.0653376	0	0	1.8362077
14.220267	13.662042	22.867134	16.224435	156.04362	177.32449	114.00664	82.230316	70.242868	117.51729
10.938667	13.662042	21.108124	14.601991	11.497951	21.26193	9.500553	17.210996	36.332518	27.543116
367.81267	197.12375	228.67134	201.18299	476.75433	565.99256	554.74968	426.45024	353.63651	354.38809
13.1264	29.275804	26.385155	22.714208	25.459749	29.341463	18.174971	11.473998	21.799511	7.3448308
370.0004	199.07547	228.67134	202.80543	477.57561	565.56733	554.74968	426.45024	353.63651	354.38809
10.938667	13.662042	21.108124	14.601991	11.497951	21.26193	9.500553	17.210996	36.332518	27.543116
367.81267	199.07547	228.67134	201.18299	477.57561	565.99256	555.16275	426.45024	353.63651	354.38809
10.938667	13.662042	21.108124	14.601991	11.497951	21.26193	9.500553	17.210996	36.332518	27.543116
370.0004	199.07547	228.67134	202.80543	477.57561	565.56733	554.74968	426.45024	353.63651	354.38809
0	0	0	0	0	0	0	0	0	0
6.2897333	1.9517203	8.7950516	1.6224435	8.6234633	10.205726	9.9136206	17.210996	4.8443357	7.3448308
7.1101333	1.9517203	10.554062	16.224435	10.676669	48.477199	8.674418	11.473998	9.6886715	16.525869
31.448667	15.613762	33.421196	35.693756	41.474752	76.117708	25.197119	30.597327	19.377343	25.706908
3.0081333	1.9517203	0	0	4.9276933	8.5047718	6.6090804	1.9123329	7.2665036	1.8362077
**150.1332**	**144.4273**	**181.17806**	**217.40742**	**243.51018**	**438.42099**	**207.77296**	**252.42795**	**220.41728**	**286.4484**
7.1101333	1.9517203	10.554062	16.224435	10.676669	48.477199	8.674418	11.473998	9.6886715	16.525869
31.448667	15.613762	33.421196	35.693756	41.474752	76.117708	25.197119	30.597327	19.377343	25.706908
3.0081333	1.9517203	0	0	4.9276933	8.5047718	6.6090804	1.9123329	7.2665036	1.8362077
**150.1332**	**144.4273**	**181.17806**	**217.40742**	**243.51018**	**438.42099**	**207.77296**	**252.42795**	**220.41728**	**286.4484**
117.59067	37.082686	33.421196	42.18353	24.227825	11.906681	13.218161	3.8246658	9.6886715	20.198285
2.1877333	0	3.5180206	0	0.8212822	0.4252386	0	0	0	0
8.4774667	7.8068812	0	1.6224435	2.8744878	3.4019087	2.0653376	0	4.8443357	7.3448308
0.2734667	0	0	1.6224435	0	0	0	0	0	1.8362077
117.59067	37.082686	33.421196	42.18353	24.227825	11.906681	13.218161	3.8246658	9.6886715	20.198285
52.232133	5.8551609	14.072082	19.469321	16.836285	6.3785789	6.1960128	5.7369988	2.4221679	12.853454
0.8204	0	0	0	0	0	0	0	0	0
52.232133	5.8551609	14.072082	19.469321	16.836285	6.3785789	6.1960128	5.7369988	2.4221679	12.853454
114.58253	37.082686	31.662186	42.18353	22.995902	11.056203	13.218161	3.8246658	9.6886715	20.198285
52.232133	5.8551609	14.072082	19.469321	16.836285	6.3785789	6.1960128	5.7369988	2.4221679	12.853454
117.59067	37.082686	33.421196	42.18353	24.227825	11.906681	13.218161	3.8246658	9.6886715	20.198285
0.5469333	0	0	0	1.2319233	0	0	0	0	0
114.30907	37.082686	31.662186	42.18353	22.995902	11.481442	13.218161	3.8246658	9.6886715	20.198285
0	0	0	0	0	0	0	0	0	0
11.4856	9.7586015	0	1.6224435	2.8744878	3.4019087	2.0653376	0	4.8443357	7.3448308
0.2734667	0	0	0	0.4106411	0	0	0	0	1.8362077
114.58253	37.082686	31.662186	42.18353	22.995902	11.481442	13.218161	3.8246658	9.6886715	20.198285
52.232133	5.8551609	14.072082	19.469321	16.836285	6.3785789	6.1960128	5.7369988	2.4221679	12.853454
117.86413	37.082686	33.421196	42.18353	24.227825	11.906681	13.218161	3.8246658	9.6886715	20.198285
52.232133	5.8551609	14.072082	19.469321	16.836285	6.3785789	6.1960128	5.7369988	2.4221679	12.853454
117.59067	37.082686	33.421196	42.18353	24.227825	11.906681	13.218161	3.8246658	9.6886715	20.198285
0.8204	0	0	3.2448869	0	0	0	0	0	0
336.63747	181.50999	119.6127	188.20344	490.71612	437.99575	548.96674	309.79793	239.79462	211.16389
3659.5309	4695.839	4754.6049	3851.6808	7452.3148	10866.122	5908.5179	5056.2082	5132.5737	6156.8044
4.9224	1.9517203	15.831093	3.2448869	6.9808988	14.883351	7.8482829	5.7369988	4.8443357	9.1810385
4443.8333	4079.0954	4439.742	2702.9908	5240.1912	7785.2681	4431.8015	3901.1592	3790.6927	4436.2778
0.2734667	0	0	0	0	0.4252386	0	0	0	0

1004.4431	880.22585	902.37229	733.34444	2973.0416	2188.703	1129.7397	764.93317	971.28932	1171.5005
0.2734667	0	0	0	0	0.4252386	0	0	0	0
1004.4431	880.22585	902.37229	733.34444	2973.0416	2188.703	1129.7397	764.93317	971.28932	1171.5005
336.63747	181.50999	119.6127	188.20344	490.71612	437.99575	548.96674	309.79793	239.79462	211.16389
13691.382	12879.402	11437.085	7614.1271	17566.405	22564.435	12983.538	10676.555	10911.866	13441.04
4.9224	1.9517203	15.831093	3.2448869	6.9808988	14.883351	7.8482829	5.7369988	4.8443357	9.1810385
4443.8333	4079.0954	4439.742	2702.9908	5240.1912	7785.2681	4431.8015	3901.1592	3790.6927	4436.2778
**250.76893**	**126.86182**	**170.624**	**76.254842**	**2264.6857**	**2589.2778**	**1936.4605**	**965.72813**	**1024.577**	**1072.3453**
534.0804	337.64761	339.48899	308.26426	802.39272	1394.3573	778.21921	600.47254	448.10106	543.51748
1.6408	0	8.7950516	3.2448869	12.319233	19.135737	5.7829453	3.8246658	4.8443357	3.6724154
**534.0804**	**335.69589**	**339.48899**	**306.64181**	**802.80336**	**1392.6564**	**777.39308**	**600.47254**	**448.10106**	**541.68127**
32.542533	11.710322	5.2770309	9.7346607	32.851289	40.822905	58.655588	3.8246658	4.8443357	9.1810385
0	0	0	0	0	0	0	0	0	0
**17558.474**	**10063.07**	**10780.974**	**12200.775**	**15305.005**	**20193.305**	**12711.74**	**9659.1936**	**12023.641**	**11009.901**
30.3548	40.986126	52.770309	17.846878	1.6425644	1.7009544	0.4130675	0	4.8443357	5.5086231
**17558.474**	**10063.07**	**10780.974**	**12200.775**	**15305.005**	**20193.305**	**12711.74**	**9659.1936**	**12023.641**	**11009.901**
0.5469333	5.8551609	5.2770309	1.6224435	2.0532055	2.9766701	2.4784051	1.9123329	4.8443357	11.017246
71.921733	54.648168	45.734268	60.030408	42.706675	63.785789	29.740862	26.772661	62.976365	27.543116
0.8204	1.9517203	1.7590103	8.1122173	0.8212822	3.4019087	2.0653376	0	0	0
**115.6764**	**185.41343**	**205.80421**	**120.06082**	**73.504758**	**145.4316**	**72.286817**	**89.879647**	**145.33007**	**128.53454**
1.6408	7.8068812	3.5180206	1.6224435	1.2319233	2.126193	2.4784051	0	2.4221679	7.3448308
5.1958667	9.7586015	10.554062	9.7346607	5.3383344	8.0795332	1.6522701	9.5616646	12.110839	18.362077
0	0	0	0	0.4106411	0.4252386	0	0	0	0
12.306	9.7586015	7.0360412	19.469321	11.497951	20.411452	24.784051	17.210996	14.533007	18.362077
0	0	0	0	0.8212822	0.8504772	0.4130675	0	0	0
13.399867	21.468923	33.421196	22.714208	7.3915399	9.355249	4.1306752	7.6493317	38.754686	9.1810385
0.5469333	0	0	0	0	0	0	0	0	0
**118.68453**	**189.31687**	**209.32223**	**121.68326**	**75.147323**	**146.28208**	**72.286817**	**91.79198**	**140.48574**	**137.71558**
1.6408	7.8068812	3.5180206	1.6224435	1.2319233	2.126193	2.4784051	0	2.4221679	7.3448308
0	0	0	0	0.4106411	1.7009544	0	0	0	0
3.8285333	0	10.554062	1.6224435	1.6425644	1.7009544	1.6522701	0	0	1.8362077
12.306	9.7586015	7.0360412	19.469321	11.497951	20.411452	24.784051	17.210996	14.533007	18.362077
0	0	0	0	0.8212822	0.8504772	0.4130675	0	0	0
13.399867	21.468923	33.421196	22.714208	7.3915399	9.355249	4.1306752	7.6493317	38.754686	9.1810385
0.5469333	0	0	1.6224435	0	0	0	0	2.4221679	0
1468.7895	950.48778	1036.0571	1734.3921	1040.9752	2110.4591	1584.527	1397.9154	1133.5746	1311.0523
0.8204	3.9034406	1.7590103	0	0.4106411	1.2757158	0.826135	0	2.4221679	3.6724154
1939.1521	1504.7763	1208.4401	1442.3522	2146.8317	3108.9193	2083.9257	1667.5543	2526.3211	2045.5354
1344.3621	1151.515	816.18078	1030.2516	1204.821	1179.1866	877.35542	841.42649	1109.3529	964.00904
0.5469333	0	1.7590103	0	1.6425644	1.7009544	2.4784051	1.9123329	0	1.8362077
0.5469333	0	0	0	3.69577	3.4019087	4.5437428	1.9123329	2.4221679	0
2.4612	3.9034406	1.7590103	1.6224435	6.1596166	2.5514315	6.6090804	11.473998	2.4221679	7.3448308
48.677067	87.827413	45.734268	111.9486	43.117316	108.0106	64.438534	36.334326	58.132029	56.922439
12.032533	7.8068812	0	6.4897738	0.4106411	1.2757158	0.4130675	0	0	5.5086231
9.8448	3.9034406	3.5180206	8.1122173	16.425644	19.135737	9.9136206	3.8246658	4.8443357	7.3448308
0	0	0	0	0	0	0	0	0	0
48.4036	87.827413	45.734268	110.32615	42.706675	106.30965	64.438534	36.334326	58.132029	56.922439

10.391733	13.662042	5.2770309	1.6224435	11.908592	11.906681	8.2613505	5.7369988	7.2665036	14.689662
9.8448	3.9034406	3.5180206	8.1122173	16.425644	19.135737	9.9136206	3.8246658	4.8443357	7.3448308
25.4324	11.710322	14.072082	17.846878	13.140515	16.584305	22.718714	9.5616646	12.110839	14.689662
10.6652	0	3.5180206	8.1122173	18.068209	20.836691	12.805093	5.7369988	0	5.5086231
0	0	0	0	0	0	0	0	0	0
0.8204	1.9517203	3.5180206	1.6224435	2.0532055	2.5514315	5.3698778	9.5616646	0	0
500.71747	628.45394	585.75043	415.34552	1318.5686	930.42204	1549.8293	1011.6241	1005.1997	1077.8539
1060.2303	2088.3407	1889.1771	1043.2311	2305.3392	1676.7158	2362.3332	3767.2959	2763.6935	4965.1056
**719.21733**	**523.06104**	**443.2706**	**332.60091**	**1341.1539**	**1628.6638**	**1368.4927**	**560.31355**	**724.22819**	**585.75026**
12.032533	3.9034406	8.7950516	3.2448869	16.425644	14.032873	21.066444	11.473998	12.110839	3.6724154
980.65147	692.8607	531.22111	392.63132	1537.0297	1951.8451	1412.6909	659.75486	758.13854	751.00895
80.3992	62.455049	26.385155	47.05086	143.72439	107.16012	154.48725	93.704313	79.93154	58.758646
40.473067	29.275804	28.144165	17.846878	39.010905	49.327677	44.198225	47.808323	29.066014	58.758646
0	0	0	0	0	0	0	0	0	0
8.4774667	9.7586015	7.0360412	11.357104	9.4447455	8.5047718	5.7829453	7.6493317	4.8443357	7.3448308
0	0	0	0	0	0	0	0	0	0
5.7428	3.9034406	1.7590103	1.6224435	30.798083	36.570519	30.980064	17.210996	7.2665036	20.198285
16.408	3.9034406	7.0360412	11.357104	36.547059	36.14528	36.349942	11.473998	21.799511	11.017246
0.8204	0	1.7590103	1.6224435	1.2319233	2.9766701	0.826135	0	0	0
1.0938667	1.9517203	1.7590103	4.8673304	7.3915399	6.8038175	3.3045402	1.9123329	2.4221679	3.6724154
13.399867	13.662042	12.313072	3.2448869	26.281031	39.547189	23.544849	19.123329	33.91035	36.724154
1489.026	985.61875	696.56808	507.8248	2115.623	1492.1622	2160.3431	973.37746	1128.7302	910.75902
1.9142667	1.9517203	0	0	5.7489755	12.331919	8.2613505	3.8246658	7.2665036	11.017246
911.19093	663.5849	446.78862	433.1924	1054.1157	860.68291	1157.4152	646.36853	617.65281	554.53473
21.603867	11.710322	12.313072	4.8673304	32.030006	38.271473	41.71982	11.473998	29.066014	14.689662
911.19093	663.5849	446.78862	433.1924	1054.1157	860.68291	1157.4152	646.36853	617.65281	554.53473
10.938667	3.9034406	10.554062	0	6.1596166	10.205726	6.1960128	1.9123329	16.955175	3.6724154
5603.6055	4147.4056	2918.1981	2337.941	7853.9218	5296.7719	7293.5333	4004.4251	4241.2159	3593.4585
179.12067	140.52386	153.0339	196.31566	152.34785	267.04984	130.9424	170.19763	130.79707	156.07765
50.317867	54.648168	54.52932	58.407964	66.113218	153.08589	59.481723	57.369988	62.976365	58.758646
8.204	3.9034406	5.2770309	8.1122173	11.08731	8.0795332	9.0874855	9.5616646	9.6886715	11.017246
779.38	458.65427	439.75258	480.24326	1079.5755	1133.6861	1314.3809	623.42053	697.58435	525.1554
545.01907	513.30244	453.82466	431.56996	1261.9001	1393.5069	763.76185	686.52752	670.9405	672.05202
733.16413	870.46725	823.21683	1189.2511	824.1567	1604	817.04756	1015.4488	930.11246	875.87107
545.01907	513.30244	453.82466	431.56996	1261.9001	1393.5069	763.76185	686.52752	670.9405	672.05202
**733.16413**	**868.51553**	**823.21683**	**1189.2511**	**823.74606**	**1603.5747**	**817.04756**	**1015.4488**	**930.11246**	**875.87107**
30.901733	46.841287	40.457237	66.520182	17.246927	48.051961	9.9136206	32.50966	19.377343	38.560362
0	1.9517203	0	0	0	0.4252386	0	0	0	0
**7610.3039**	**3413.5588**	**4367.6226**	**2597.532**	**5427.8542**	**6313.0921**	**5355.8335**	**3428.8129**	**2640.163**	**2978.3289**
33.089467	21.468923	10.554062	22.714208	27.923595	34.444326	18.174971	17.210996	19.377343	31.215531
0	0	0	0	0	0	0	0	0	0
3.5550667	3.9034406	0	3.2448869	4.9276933	12.757158	7.4352154	3.8246658	2.4221679	1.8362077
0	0	0	0	0	0	0	0	0	0
7.6570667	1.9517203	3.5180206	4.8673304	11.497951	12.331919	9.0874855	3.8246658	7.2665036	7.3448308
**463.79947**	**368.87514**	**422.16247**	**433.1924**	**255.82941**	**315.95227**	**182.98891**	**112.82764**	**104.15322**	**156.07765**
0.5469333	0	0	0	0.8212822	0.4252386	0.826135	0	0	0
**58.795333**	**119.05494**	**200.52718**	**131.41792**	**133.04772**	**278.10604**	**117.72424**	**244.77861**	**247.06112**	**246.05183**

0	0	0	0	0	0	0	0	0	0
0.2734667	0	0	0	0	0	0	0	0	0
3.8285333	0	0	1.6224435	2.4638466	3.4019087	0.4130675	1.9123329	2.4221679	0
1.0938667	0	5.2770309	1.6224435	2.8744878	8.9300104	5.7829453	1.9123329	2.4221679	1.8362077
0.5469333	1.9517203	3.5180206	0	1.6425644	2.126193	0	1.9123329	2.4221679	1.8362077
0	0	0	0	0	0	0	0	0	0
0	0	0	0	39.421546	34.019087	29.327794	22.947995	16.955175	20.198285
8.4774667	11.710322	8.7950516	11.357104	0	0	0	0	0	0
0	0	0	0	0	0	0	0	0	0
8.4774667	11.710322	8.7950516	11.357104	0	0	0	0	0	0
0	0	0	0	0	0	0	0	0	0
**95.439867**	**46.841287**	**49.252289**	**42.18353**	**19.710773**	**27.640508**	**28.088592**	**21.035662**	**14.533007**	**11.017246**
0.2734667	0	0	1.6224435	0.4106411	0	0	0	0	0
0	0	0	0	0	0	0	0	0	0
31.448667	5.8551609	21.108124	9.7346607	0	0	0	1.9123329	0	0
0	0	0	0	0	0	0	0	0	0
31.448667	5.8551609	21.108124	9.7346607	0	0	0	1.9123329	0	0
0	0	0	0	0	0	0	0	0	0
31.448667	5.8551609	21.108124	9.7346607	0	0	0	1.9123329	0	0
0	9.7586015	0	0	0.4106411	1.2757158	3.7176077	0	7.2665036	3.6724154
**100.0888**	**85.875693**	**87.950516**	**95.724164**	**142.49246**	**130.97349**	**86.74418**	**82.230316**	**70.242868**	**112.00867**
0.5469333	0	0	0	0.4106411	0	1.2392026	0	0	0
2.4612	1.9517203	0	0	0	0.4252386	0	0	0	0
0	0	0	0	0.4106411	0.4252386	0	0	0	0
4.6489333	1.9517203	1.7590103	0	3.69577	2.9766701	2.0653376	0	2.4221679	5.5086231
0.2734667	0	0	0	0.4106411	0	0.4130675	0	0	0
10.391733	1.9517203	3.5180206	4.8673304	4.1064111	9.355249	7.4352154	5.7369988	4.8443357	11.017246
0.2734667	0	0	0	0.4106411	0	0.4130675	0	0	0
10.391733	1.9517203	3.5180206	4.8673304	4.1064111	9.355249	7.4352154	5.7369988	4.8443357	11.017246
0.2734667	0	0	0	0.4106411	0	0.4130675	0	0	0
10.391733	1.9517203	3.5180206	4.8673304	4.1064111	9.355249	7.4352154	5.7369988	4.8443357	11.017246
0.2734667	0	0	0	0.4106411	0	0.4130675	0	0	0
10.391733	1.9517203	3.5180206	4.8673304	4.1064111	9.355249	7.4352154	5.7369988	4.8443357	11.017246
0.2734667	0	0	0	0.4106411	0	0.4130675	0	0	0
10.391733	1.9517203	3.5180206	4.8673304	4.1064111	9.355249	7.4352154	5.7369988	4.8443357	11.017246
0.2734667	0	0	0	0.4106411	0	0.4130675	0	0	0
10.391733	1.9517203	3.5180206	4.8673304	4.1064111	9.355249	7.4352154	5.7369988	4.8443357	11.017246
**33.362933**	**19.517203**	**31.662186**	**22.714208**	**0**	**0**	**0**	**0**	**0**	**0**
0	0	0	0	0	0	0	0	0	0
0	0	0	0	25.049108	49.752915	12.805093	9.5616646	2.4221679	1.8362077
0	0	0	0	0.8212822	2.5514315	0.4130675	0	0	0
0	0	0	0	0	0	0	0	0	0
3.5550667	3.9034406	8.7950516	3.2448869	2.4638466	8.5047718	4.9568103	3.8246658	2.4221679	1.8362077
0.2734667	0	0	0	1.2319233	0.8504772	1.2392026	0	2.4221679	0
2.1877333	5.8551609	1.7590103	1.6224435	3.69577	2.9766701	4.5437428	3.8246658	0	5.5086231

5.1958667	5.8551609	3.5180206	6.4897738	14.78308	22.962884	12.805093	5.7369988	9.6886715	9.1810385
0.2734667	0	0	0	0	0	0	1.9123329	0	0
0	0	0	1.6224435	0	0	0	0	0	0
1.3673333	0	0	0	2.0532055	0.8504772	0.4130675	1.9123329	0	1.8362077
3.2816	3.9034406	3.5180206	1.6224435	2.0532055	5.1028631	6.1960128	0	7.2665036	3.6724154
0.8204	1.9517203	0	0	1.2319233	1.7009544	0.4130675	3.8246658	0	1.8362077
0.2734667	0	0	1.6224435	10.266028	11.481442	9.0874855	5.7369988	4.8443357	1.8362077
0	0	0	0	0	0	0	0	0	0
0.2734667	0	0	1.6224435	10.266028	11.481442	9.0874855	5.7369988	4.8443357	1.8362077
0	0	0	0	0	0	0	0	0	0
0	0	0	0	0.8212822	1.2757158	1.2392026	0	2.4221679	0
**60.983067**	**68.31021**	**40.457237**	**16.224435**	**91.983608**	**156.06256**	**93.35326**	**80.317983**	**65.398533**	**67.939685**
1.9142667	1.9517203	0	0	1.6425644	0.4252386	0.826135	1.9123329	0	1.8362077
1.6408	0	1.7590103	3.2448869	3.2851289	1.2757158	3.3045402	3.8246658	0	3.6724154
0	0	0	0	0	0.4252386	0.826135	0	0	0
0	0	0	0	5.3383344	3.8271473	6.1960128	3.8246658	0	1.8362077
1.9142667	0	0	1.6224435	1.6425644	0.8504772	0.4130675	1.9123329	0	0
10.938667	3.9034406	5.2770309	1.6224435	11.08731	12.331919	16.935768	3.8246658	9.6886715	3.6724154
0.5469333	1.9517203	1.7590103	0	0.8212822	2.9766701	1.2392026	1.9123329	0	0
10.938667	9.7586015	3.5180206	12.979548	11.08731	30.19194	9.500553	3.8246658	4.8443357	7.3448308
**1.3673333**	**0**	**1.7590103**	**0**	**42.296034**	**42.09862**	**30.980064**	**38.246658**	**31.488182**	**31.215531**
0	0	0	0	0.4106411	0	0	0	4.8443357	0
**558.96587**	**446.94395**	**392.2593**	**311.50914**	**57.489755**	**53.580062**	**40.480617**	**57.369988**	**48.443357**	**49.577608**
0	0	0	0	0	0	0	0	0	0
0	0	0	0	0	0	0	0	0	0
0	0	0	0	39.832187	44.650052	28.088592	28.684994	16.955175	22.034492
0	0	0	0	0	0	0	0	0	0
3.0081333	0	1.7590103	4.8673304	13.140515	5.5281017	7.0221479	5.7369988	2.4221679	5.5086231
0	0	0	0	0	0	0	0	0	0
3.0081333	0	1.7590103	4.8673304	13.140515	5.5281017	7.0221479	5.7369988	2.4221679	5.5086231
0	0	0	0	45.991804	51.028631	67.743074	38.246658	31.488182	16.525869
0	0	0	0	5.3383344	5.1028631	2.8914727	1.9123329	0	0
0	0	0	1.6224435	0.8212822	0.4252386	0	0	0	0
0	0	0	0	0	0	0	0	0	0
4.3754667	7.8068812	7.0360412	8.1122173	7.802181	5.1028631	9.0874855	3.8246658	7.2665036	11.017246
0	0	0	0	0.4106411	0	0	0	0	0
6.2897333	0	1.7590103	1.6224435	6.1596166	4.2523859	1.6522701	1.9123329	0	0
3.0081333	5.8551609	0	1.6224435	0	0	0	0	0	0
1.3673333	0	0	0	1.6425644	1.2757158	3.3045402	0	0	1.8362077
1.0938667	0	0	0	4.1064111	2.126193	6.1960128	3.8246658	2.4221679	1.8362077
1.3673333	0	0	0	1.6425644	1.2757158	3.3045402	0	0	1.8362077
1.0938667	0	0	0	4.1064111	2.126193	6.1960128	3.8246658	2.4221679	1.8362077
1.3673333	0	0	0	1.6425644	1.2757158	3.3045402	0	0	1.8362077
1.0938667	0	0	0	4.1064111	2.126193	6.1960128	3.8246658	2.4221679	1.8362077
1.3673333	0	0	0	1.6425644	1.2757158	3.3045402	0	0	1.8362077
1.0938667	0	0	0	4.1064111	2.126193	6.1960128	3.8246658	2.4221679	1.8362077
1.0938667	0	0	1.6224435	1.2319233	1.2757158	0	0	0	1.8362077

0	0	0	0	0	0	0	0	0	0
1.0938667	0	0	0	0.8212822	0	0.826135	0	0	1.8362077
0	0	0	0	0	0	0	0	0	0
2.1877333	3.9034406	3.5180206	4.8673304	0	0.4252386	0	0	0	0
0.2734667	1.9517203	1.7590103	1.6224435	0	0.4252386	0	0	0	0
**39.926133**	**39.034406**	**40.457237**	**38.938643**	**6.1596166**	**6.8038175**	**4.1306752**	**3.8246658**	**0**	**5.5086231**
1.3673333	1.9517203	1.7590103	4.8673304	0.4106411	0.4252386	0	1.9123329	0	0
0	0	0	0	0	0	0	0	0	0
1.0938667	1.9517203	1.7590103	1.6224435	0.4106411	0.4252386	0	0	0	0
0.5469333	0	0	0	1.2319233	0.8504772	0.4130675	1.9123329	0	1.8362077
6.2897333	3.9034406	3.5180206	3.2448869	11.908592	9.7804876	9.500553	9.5616646	4.8443357	9.1810385

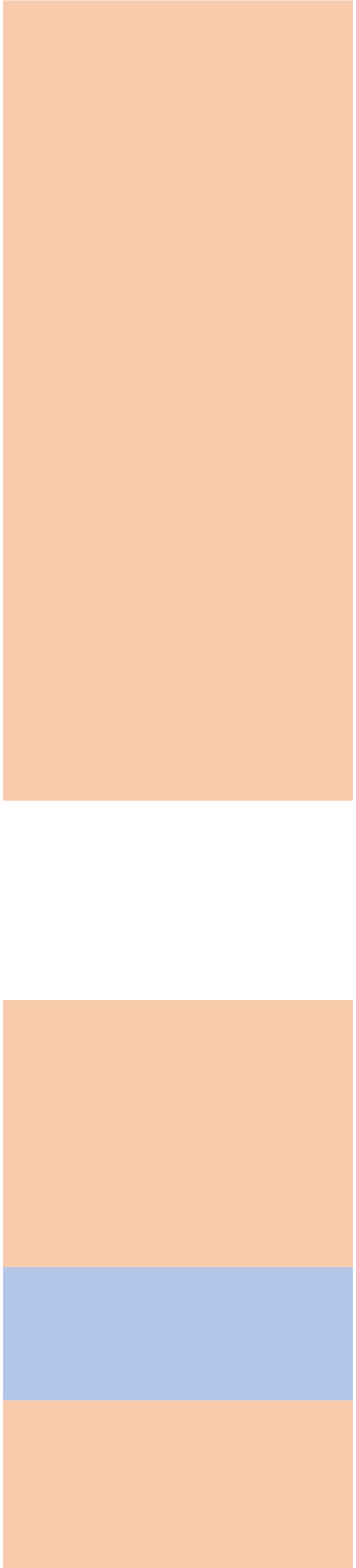

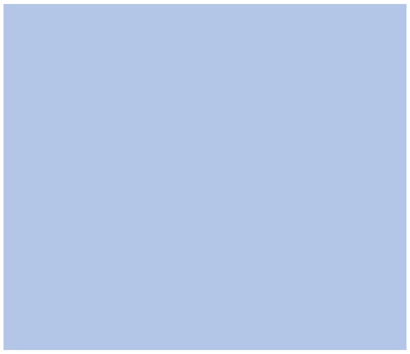

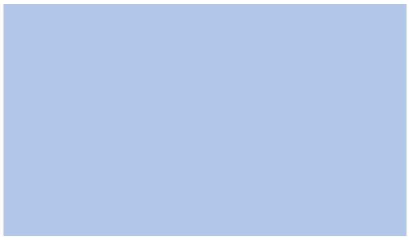

See Hunt 2019
