# Supplementary material for: Laccase Directed Lignification Is One of the Major Processes Associated With the Defense Response Against Pythium ultimum Infection in Apple Roots
Source: Front Plant Sci. 2021 Sep 7;12:629776. doi: 10.3389/fpls.2021.629776 (PMC8453155; doi:10.3389/fpls.2021.629776)
Supplement: Supplementary file 2 [file Data_Sheet_2.PDF]

miRNA	Target gene ID	Cleavage site	miRNA sequence
miR10986	HF12097-RA	1692	UGGCACCAAAGUCACCACCCG
miR10986	HF12099-RA	1692	UGGCACCAAAGUCACCACCCG
miR10986	HF12100-RA	930	UGGCACCAAAGUCACCACCCG
miR10986	HF12105-RA	1686	UGGCACCAAAGUCACCACCCG
miR10986	HF17936-RA	1752	UGGCACCAAAGUCACCACC-CG
miR10986	HF17941-RA	1767	UGGCACCAAAGUCACCACC-CG
miR1511a	HF21591-RA	509	ACCUAGCUCUGAUACCAUAAA
miR1511e	HF13566-RA	160	ACCUAGCUCUGAUACCAUGAU
miR156a	HF26192-RA	714	UGACAGAAGAAAGUGAGCAC
miR156c	HF07614-RA	1086	UGACAGAAGAGAGUGAGCACA
miR156d	HF05339-RA	784	UUGACAGAAGAGAGAGAGCACA
miR156d	HF08888-RA	967	UUGACAGAAGAGAGAGAGCACA
miR156e	HF39039-RA	793	CUGACAGAAGAUAGAGAGCAC
miR156f	HF20344-RA	805	UUGACAGAAGAUAGAGAGCAC
miR156f	HF26180-RA	1192	UUGACAGAAGAUAGAGAGCAC
miR156f	HF42804-RA	1165	UUGACAGAAGAUAGAGAGCAC
miR156i	HF08888-RA	966	UGACAGAAGAGAGUGAGCACA
miR156k	HF32947-RA	781	AUGACAGAAGAUAGAGAGUAC
miR156l	HF05261-RA	480	GCUUUCUCUUCUUCUGUCAUC
miR156n	HF35297-RA	1192	UUGACAGAAGAUAGAGAGCAC
miR156o	HF42324-RA	1086	UGACAGAAGAGAGUGAGCACA
miR156u	HF06428-RA	1173	UGACAGAAGAGAGUGAGCACA
miR156u	HF09374-RA	987	UGACAGAAGAGAGUGAGCACA
miR156u	HF41522-RA	966	UGACAGAAGAGAGUGAGCACA
miR156v	HF42804-RA	1164	UGACAGAAGAGAGGGAGCAU
miR159a	HF03195-RA	763	UUUGGAUUGAAGGGAGCUCUA
miR159a	HF03914-RA	775	UUUGGAUUGAAGGGAGCUCUA
miR159b	HF17403-RA	610	CUUGGAUCGAAUGGAGCUCC
miR159c	HF06666-RA	970	UUUGGAUUGAAGGGAGCUCUA
miR159c	HF12011-RA	1958	UUUGGAUUGA-AGGGAGC-UCUA
miR160a	HF06172-RA	1370	UGCCUGGCUCCCUGUAUGCCA
miR160a	HF40525-RA	1367	UGCCUGGCUCCCUGUAUGCCA
miR160a	HF44485-RA	1313	UGCCUGGCUCCCUGUAUGCCA
miR160d	HF00444-RA	767	UGC-CUGGCUCCCUG-UAUGCCA
miR160d	HF01570-RA	1370	UGCCUGGCUCCCUGUAUGCCA
miR160d	HF04836-RA	1370	UGCCUGGCUCCCUGUAUGCCA
miR160d	HF07133-RA	1337	UGCCUGGCUCCCUGUAUGCCA
miR160d	HF24306-RA	1334	UGCCUGGCUCCCUGUAUGCCA
miR160d	HF25001-RA	1352	UGCCUGGCUCCCUGUAUGCCA
miR162b	HF05621-RA	3225	UCGAUAA-ACCUCUGCAUCCAG
miR164a	HF09293-RA	655	UGGAGAAGCAGGGCACGUGCA
miR164a	HF24823-RA	700	UGGAGAAGCAGGGCACGUGCA
miR164h	HF11267-RA	637	UGGAGAAGCAGGGCACAUGCC
miR164h	HF16100-RA	3288	UGGAGAAGCAGGGCA-CAUGCC
miR164h	HF22809-RA	628	UGGAGAAGCAGGGCACAUGCC

miR166a	HF00268-RA	562	UCGGACCAGGCUUCAUUCCCC
miR166a	HF12939-RA	577	UCGGACCAGGCUUCAUUCCCC
miR166a	HF21765-RA	586	UCGGACCAGGCUUCAUUCCCC
miR166a	HF40749-RA	1012	UCGGACCAGGCUUCA-UUCCCC
miR166j	HF06176-RA	571	UCGGACCAGGCUUCAUUCCCC
miR166j	HF11732-RA	598	UCGGACCAGGCUUCAUUCCCC
miR166j	HF23204-RA	598	UCGGACCAGGCUUCAUUCCCC
miR166j	HF28547-RA	562	UCGGACCAGGCUUCAUUCCCC
miR166j	HF40517-RA	571	UCGGACCAGGCUUCAUUCCCC
miR167a	HF32107-RA	2414	UGAAGCUGCCAGCAUGAUCUA
miR167f	HF37005-RA	2258	UGAAGCUGCCAGCAUGAUCUA
miR167g	HF11771-RA	2429	UGAAGCUGCCAGCAUGAUCU
miR167g	HF34014-RA	2933	UGAAGCUGCCAGCAUGAUCU
miR167h	HF18290-RA	2423	UGAAGCUGCCAGCAUGAUCU
miR167h	HF29114-RA	3116	UGAAGCUGCCAGCAUGAUCU
miR168b	HF09608-RA	550	UCGCUUGGUGCAGGUCGGGAA
miR168b	HF36007-RA	285	UCGCU--UGGUGCAGG-UCGGGAA
miR169d	HF19226-RA	1388	UAGCCAGGGAUGACUUGC-CU
miR171a	HF07177-RA	454	UGAGCCGAACCAAUAUCACUC
miR171a	HF44555-RA	451	UGAGCCGAACCAAUAUCACUC
miR171b	HF00464-RA	5091	UUGAGCCGCGCCAAUAUCACU
miR171b	HF27082-RA	1241	UUGAGCCGCGCCAAUAUCACU
miR171b	HF37501-RA	1373	UUGAGCCGCGCCAAUAUCACU
miR171c	HF10118-RA	940	UUGAGCCGUGCCAAUAUCACA
miR171g	HF01470-RA	1364	UUGAGCCGCGUCAAUAUCUCC
miR172a	HF01637-RA	1378	AGAAUCUUGAUGAUGCUGCAG
miR172a	HF27401-RA	1651	AGAAUCUUGAUGAUGCUGCAG
miR172d	HF40428-RA	1270	AGAAUCUUGAUGAUGCUGCAU
miR172f	HF04766-RA	1273	AGAAUCUUGAUGAUGCUGCAG
miR172f	HF31388-RA	1561	AGAAUCUUGAUGAUGCUGCAG
miR172g	HF06289-RA	1282	AGAAUCUUGAUGAUGCUGCAU
miR172g	HF24247-RA	1276	AGAAUCUUGAUGAUGCUGCAU
miR172g	HF25060-RA	1270	AGAAUCUUGAUGAUGCUGCAU
miR2111c	HF32972-RA	248	UAAUCUGCAUCCUGAGGUUUA
miR2118b	HF07401-RA	1006	CUACCGAU-GCCACUAAGUCCCA
miR2118b	HF17618-RA	158	CUACCGAUGCCACUA-AGUCCCA
miR319c	HF16566-RA	964	UUUGGACUGAAGGGAGCUCC
miR319f	HF03499-RA	1099	CUUGGAUUGAAGGGAGCUCC
miR390a	HF43010-RA	372	AAGCUCAGGAGGGAUAG-CGCC
miR395a	HF01798-RA	345	CUGAAGUGUUUGGGGGAACUC
miR395a	HF07090-RA	101	CUGAAGUGUUUGGGGGAACUC
miR395a	HF13989-RA	732	CUGAAGUGUUUGGGGGAACUC
miR395b	HF35421-RA	140	CUGAAGUGUUUGGGGGAACCC
miR395e	HF09045-RA	619	UGAAGUGUUUGGGGGAACUC
miR396a	HF00473-RA	359	UUCCACAG-CUUUCUUGAACUG
miR396a	HF07761-RA	2112	UUCCACAGCUUUCUUGAACUG
miR396a	HF21477-RA	818	UUCCACAG-CUUUCUUGAACUG

miR396a	HF27291-RA	761	UUCCACAG-CUUUCUUGAACUG
miR396a	HF30789-RA	362	UUCCACAG-CUUUCUUGAACUG
miR396a	HF42039-RA	69	UUCCACAGCUUUCUUGAACUG
miR396b	HF08723-RA	689	UUCCACAG-CUUUCUUGAACUU
miR396b	HF16426-RA	338	UUCCACA-GCUUUCUUGAACUU
miR396b	HF26054-RA	545	UUCCACA-GCUUUCUUGAACUU
miR396b	HF27171-RA	485	UUCCACA-GCUUUCUUGAACUU
miR396b	HF36486-RA	557	UUCCACAG-CUUUCUUGAACUU
miR396c	HF02750-RA	525	UUCCACGG-CUUUCUUGAACUG
miR396c	HF41682-RA	758	UUCCAC-GGCUUUCUUGAACUG
miR396d	HF19974-RA	557	UUCCAC-GGCUUUCUUGAACUG
miR396e	HF10798-RA	564	UUCCACAGCUUUCUUGAACAG
miR396e	HF41728-RA	868	UUCCACAGCUUUCUUGAACAG
miR396f	HF10150-RA	347	UUCCACA-GCUUUCUUGAACUU
miR396f	HF13165-RA	737	UUCCACAG-CUUUCUUGAACUU
miR396f	HF19000-RA	2702	UUCCACAG-CUUUCUUGAACUU
miR396f	HF31510-RA	782	UUCCACAG-CUUUCUUGAACUU
miR397b	HF23917-RA	713	UUGAGUGCAGCGUUGAUGAAA
miR397b	HF26400-RA	662	UUGAGUGCAGCGUUGAUGAAA
miR397b	HF27792-RA	647	UUGAGUGCAGCGUUGAUGAAA
miR397b	HF40034-RA	779	UUGAGUGCAGCGUUGAUGAAA
miR398a	HF42086-RA	999	UGUGUUCUCAGGUUGCCCCUG
miR398b	HF25617-RA	44	UGUGUUC-UCAGGUCGCCCCUG
miR398c	HF06452-RA	44	UGUGUUC-UCAGGUCGCCCCUG
miR398c	HF30403-RA	941	UGUGUUC--------UCAGGUCGCCCCUG
miR398d	HF01373-RA	728	UGUGUUCUCAGGUCACC--------CCUU
miR398d	HF08261-RA	740	UGUGUUCUCAGGUCACCCCUU
miR398d	HF29451-RA	609	UGUGUUCUCAGGUCACCCCUU
miR398d	HF40618-RA	440	UGUGUU-CUCAGGUCACCCCUU
miR398d	HF41261-RA	543	UGUGUUCUCAGGUCACCCCUU
miR399g	HF05992-RA	514	CUGCCAAAGGAGAUCUGCUCAG
miR408	HF17186-RA	16	AUGCACUGCCUCUUCCCU-GGC
miR408	HF20292-RA	585	AUGCAC-UGCCUCUUCCCUGGC
miR477a	HF05240-RA	768	ACUCUCCCUCAAGGGCUUCGAC
miR477b	HF41450-RA	798	UCCCUCAAGGGCUUCCAAUAUU
miR482a	HF00026-RA	417	UCUUUCCUAUCCCUUCCAUUCC
miR482a	HF32497-RA	426	UCUUUCCUAUCCCUUCCAUUCC
miR482c	HF43810-RA	655	UCUUUCCUAUCCCUCCCAUUCC
miR482d	HF01646-RA	727	UCUUUCCUAACCCUCCCAUUCC
miR482d	HF04762-RA	622	UCUUUCCUAACCCUCCCAUUCC
miR482d	HF07514-RA	455	UCUUUCCUAACCCUCCCAUUCC
miR482d	HF40153-RA	583	UCUUUCCUAACCCUCCCAUUCC
miR5139	HF09572-RA	1018	CGAAACCUGGCUCUGAUACC
miR535b	HF02059-RA	220	UGACAAGGAGAGAGAGCACGC
miR535d	HF32583-RA	76	UGACGACGAGAGAGAGCACGC
miR7122b	HF44234-RA	281	UUAUACAGAGAAAUCACGGUCG
miR7125	HF11513-RA	144	CGAACUUAUUGCAACUAGCUU

miR7782	HF21767-RA	147	ACUUGGCUCUGAUACCAUGAA
miR858	HF00466-RA	240	UUCGUUGUCUGUUCGACCUGA
miR858	HF08482-RA	300	UUCGUUGUCUGUUCGACCUGA
miR858	HF13276-RA	300	UUCGUUGUCUGUUCGACCUGA
miR858	HF13279-RA	300	UUCGUUGUCUGUUCGACCUGA
miR858	HF16086-RA	216	UUCGUUGUCUGUUCGACCUGA
miR858	HF18993-RA	300	UUCGUUGUCUGUUCGACCUGA
miR858	HF21423-RA	300	UUCGUUGUCUGUUCGACCUGA
miR858	HF21717-RA	357	UUCGUUGUCUGUUCGACCUGA
miR858	HF24028-RA	354	UUCGUUGUCUGUUCGACCUGA
miR858	HF28765-RA	303	UUCGUUGUCUGUUCGACCUGA
miR858	HF29485-RA	300	UUCGUUGUCUGUUCGACCUGA
miR858	HF30785-RA	300	UUCGUUGUCUGUUCGACCUGA
miRN14	HF23616-RA	1416	CACAUGUCAAUUGAUUAGGUG
miRN28	HF05256-RA	992	UCAUUUG-GCAUCUCUUUUUCGU
miRN39	HF24680-RA	316	CCGUCUCCC-ACUCUUCUUCUU

Target site sequence	Degradome ca	Degradome p-value	Tag abundance
UUGGUGGUGACUUUGGUGCCG	0	0.001679179	92
UUAGUGGUGACUUUGGUGCCA	0	0.005029084	1417
UUGGUGGUGACUUUGGUGCCA	0	0.000839942	1390
UUGGUAGUGACUUUGGUGCCG	0	0.013769199	146
CGUGGUGGUGACUUUGGUGCCC	0	0.000420059	105
UGUGGUGGUGACUUUAGUGCCC	1	0.026270605	3
UCUGUGGUAUCAGGGCGAGAG	0	0.038724165	7
AUCAUGAUAUCAGAGC-GGGU	0	0.043960264	2
GUGCUCUCUCUUCUGUCA	0	0.002517711	75
UGUGCUCUCUCUUCUGUCA	0	0.001259649	1096
UGUGCUCUCUCUCUUCUGUCAA	0	0.002098533	89
UGUGCUCUCUCUUCUGUCAU	2	0.028858663	22
GUGCUCUCUAUCUUCUGUCAU	2	0.028858663	7
GUGCUCUCUCUCUUCUGUCAA	0	0.000839942	79
GUGCUCUCUCUCUUCUGUCAA	0	0.001679179	47
GUGCUCUCUCUCUUCUGUCAA	0	0.001259649	48
UGUGCUCUCUCUCUUCUGUCA	0	0.002098533	61
GUGCCCUCUAUCUUCUGUCAU	0	0.002936713	20
GA-GAUAGAAGGAGGGGGAGC	0	0.036297841	10
GUGCUCUCUCUCUUCUGUCAA	0	0.001679179	56
UGUGCUCUCUCUCUUCUGUCA	0	0.001259649	124
UGUGCUCGCUCUCUUCUGUCA	0	0.000420059	157
UGUGCUCUCUCUCUUCUGUCA	0	0.002517711	153
UGUGCUCUCUCUCUUCUGUCA	0	0.002098533	36
GUGCUCUCUCUCUUCUGUCA	2	0.028858663	27
UAGAGCCCCCUUCAAACCAAA	0	0.009200662	32
UAGAGCCCCCUUCAAACCAAA	0	0.009616856	42
GGAGCUCCAUUCACUCCAAU	0	0.008367748	6
UGGAGCUCCCUUCACUCCAAU	0	0.000420059	119
GGGAGGCUCCCAGUCGAUCCAGA	1	0.02682199	6
AGGCAUACAGGGAGCCAGGCA	0	0.001259649	47
AGGCAUACAGGGAGCCAGGCA	0	0.001679179	48
GGGCAUGCAGGGAGCCAGGCA	0	0.000839942	1056
UGGCAGUUCAGGGAGUCAGUGCA	0	0.010448721	12
UGGCAUGCAGGGAGCCAGGCA	0	0.002098533	65
UGGCAUGCAGGGAGCCAGGCA	0	0.003355539	64
GGGCAUGCAGGGAGCCAGGCA	0	0.002936713	1026
AGGCAUACAGGGAGCCAGGCA	0	0.000420059	84
AGGCAUACAGGGAGCCAGGCA	0	0.002517711	69
CUGGAUGCAGAGGUGUUAUCGA	0	0.000420059	61
AUUACGUGCCCUGCUUCUCCA	0	0.001259649	653
AUUACGUGCCCUGCUUCUCCA	0	0.001679179	642
AGCAAGUGCCCUGCUUCUCCA	0	0.000420059	588
GGCAUGUUGUGCUCCUUCUCCA	0	0.039531581	5
AGCAAGUGCCCUGCUUCUCCA	0	0.000839942	559

CUGGAAUGAAGCCUGGUCCGG	0	0.000839942	174
CUGGGAUGAAGCCUGGUCCGG	0	0.001259649	192
CUGGGAUGAAGCCUGGUCCGG	0	0.001679179	174
GAGGAUUUGAAGCCUGGUCCGG	0	0.007534133	19
CUGGGAUGAAGCCUGGUCCGG	0	0.002517711	182
UUGGGAUGAAGCCUGGUCCGG	0	0.003355539	119
UUGGGAUGAAGCCUGGUCCGG	0	0.002098533	114
CUGGAAUGAAGCCUGGUCCGG	0	0.000420059	207
CUGGGAUGAAGCCUGGUCCGG	0	0.002936713	446
UAGAUCAGGCUGGCAGCUUGU	0	0.000839942	620
UAGAUCAGGCUGGCAGCUUGU	0	0.000420059	648
AGAUCAGGCUGGCAGCUUGU	0	0.001679179	95
AGAUCAGGCUGGCAGCUUGU	0	0.001259649	95
AGAUCAGGCUGGCAGCUUGU	0	0.002517711	85
AGAUCAGGCUGGCAGCUUGU	0	0.002098533	78
UUCCCGACCUGCACCAAGCGA	0	0.000420059	422
UUCCGGGUCCUGCACCACCGGCGA	0	0.014183474	5
GGAGCAAGUCGUCCCUGGUGA	1	0.001132203	2
GGGUGAUAUUGGUUCGGCUCA	0	0.000420059	12
GGGUGAUAUUGUUUCGGCUCA	0	0.000839942	11
UGUGAUAUUGGUGGUGCUCAU	0	0.023253639	4
AGGGAUAUUGGCGCGGCUCAA	0	0.001259649	990
AGGGAUAUUGGCGCGGCUCAA	0	0.000839942	15
UGUGAUAA-GGCGAGGCUCAA	0	0.017078535	4
AGGGAUAUUGGCGCGGCUCAA	0	0.000420059	15
CUGCAGCAUCAUCAGGAUUCC	0	0.000839942	18
CUGCAGCAUCAUCAGGAUUCC	0	0.001259649	7
CUGCAGCAUCAUCAGGAUUCU	0	0.000839942	453
CUGCAGCAUCAUCAGGAUUCC	0	0.000420059	3
CUGCAGCAUCAUCAGGAUUCC	0	0.002098533	13
CUGCAGCAUCAUCAGGAUUCU	0	0.000420059	467
UUGCAGCAUCAUCAGGAUUCC	0	0.003355539	61
UUGCAGCAUCAUCAGGAUUCC	0	0.002517711	91
UAAACCGCAGGAUGCAGAUUA	0	0.000420059	85
GGGGACAUGGUGGUGGUCGGUGC	1	0.04213467	3
UGGGGCGGUGGUGGUAGUGGUGG	0	0.016665476	7
GGAGCUCCCUUCACUCCAAU	0	0.000420059	118
GGAGCUCCCUUCAAACCAAG	0	0.000420059	4
CUUGUCUAUCCCUCCUGAGCUG	0	0.005029084	31
GAGUUCCUCCAAACUCUUCAU	0	0.001679179	223
GAGUUCACCCAAACACUUCAA	0	0.000839942	3
GAGUUCCUCCAAACUCUUCAU	0	0.001259649	105
AGGUUCCACCAAACACUUCAA	0	0.005864802	12
GAGCUCCCCCAGGCACUUCG	2	0.028858663	2
CCGUUCAAGAAAGCCUGUGGAA	0	0.002517711	2
CGGUUGAAGACUGCUGUGGAA	0	0.016665476	21
CCGUUCAAGAAAGCCUGUGGAA	0	0.003355539	170

UCGUUCAAGAAAGCCUGUGGAA	0	0.002098533	113
CCGUUCAAGAAAGCCUGUGGAA	0	0.000839942	73
CAGAAGAAGAAAGCUGUGGAG	0	0.020788266	3
CCGUUCAAGAAAGCCUGUGGAA	0	0.005029084	24
UCGUUCAAGAAAGCUUGUGGAA	0	0.005447031	87
CCGUUCAAGAAAGCAUGUGGAA	1	0.001132203	2
GCGUUCAAGAAAGCAUGUGGAA	0	0.001679179	20
CCGUUCAAGAAAGCCUGUGGAA	0	0.001259649	244
AAGUUCGAGAAGGCCCGUGGAA	2	0.028858663	42
CCGUUCAAGAAAGCCUGUGGAU	0	0.005864802	5
CCGUUCAAGAAAGCCUGCGGAA	0	0.031019776	17
GUGUUCGAGAAAGCUGCAGAA	0	0.047968617	14
CUAUUCAAGGAAGCUGUGGAU	0	0.006282398	4
UCGUUCAAGAAAGCUUGUGGAA	0	0.004610961	182
CCGUUCAAGAAAGCCUGUGGAA	0	0.002098533	201
CCGUUCAAGAAAGCCUGUGGAA	0	0.000420059	39
UCGUUCAAGAAAGCCUGUGGAA	0	0.004192663	108
AGUCAUCAACGCUGCACUCAA	2	0.028858663	2
CCUAAUCAACGCUGCACUCAA	0	0.004192663	30
CCUAAUCAACGCUGCACUCAA	0	0.003355539	45
AGUCGUCAACUCUGCACUCAA	0	0.011695208	15
CUGGGGCCAUCUGAGAUCACA	0	0.034676891	24
CAAGGGCGACCUGACGAACACA	0	0.001679179	110
CAAGGGCGACCUGACGAACACA	0	0.002098533	92
CAGGGGCGACCUGACCUUCAAAGAACAC/	0	0.000420059	193
AAGGAGCCGCUUGGUGACCUGGGAACAC	0	0.005029084	283
GCUUGGUGACCUGGGAACACU	0	0.005447031	290
AAGGGAGGACCUGAGAACUCU	0	0.011279887	12
UGCGGGUGACCUGGGAAACAUA	0	0.009616856	17
AAGAGGUGA-CUGAGAACACA	0	0.037511768	64
UUGAGCAGGUCUCCAUGGGCAA	1	0.002263124	4
GCUAAGGGAAGAGGCAGUGCAG	1	0.001132203	2
ACCAGGGAAGAAGCAUGUGCAG	1	0.047005251	3
AGAGAGGCCCCGGAGGGAGAGA	0	0.005447031	7
AAUAUUGGAAGCCUUUGAGGGA	0	0.000420059	3
GGAAUGGAUGGGAUGGGAAGGA	0	0.000839942	194
GGAAUGGAUGGGAUGGGAAGGA	0	0.000420059	191
GGAAUGGGAGGCAUAGGCAAGA	0	0.025303373	9
GGAAUGGGAGGCUUGGGAAAAA	0	0.011695208	4
GGAAUGGGAGGCCUGGGCAAGA	0	0.045565626	11
GGAAGGGGAGGGUUCCGAAAGA	0	0.042352202	16
GGGAUGGGCGGUUUGGGGAAGA	0	0.035082384	4
GGUGUU-GAGUCAGGUGGCG	0	0.049167842	10
GCGUGCUCUCUCUCCUCGUCU	1	0.000566262	2
UCGUGUUCUCUCUCGUCCAGU	0	0.010032876	17
CGGCCGUGAUUUCUUUGUAUAA	0	0.000420059	167
AAGCUAGUUGCAAUAAGUUCA	0	0.000420059	189

GCGAUGGUAUCAGAGCCAUAC	0	0.012940125	8
CCAGGCCGAACAGACAAUGAA	0	0.004610961	56
CCGGGAAGAACAGACAACGAG	1	0.019074097	3
CCGGGGCGAACAGACAAUGAA	0	0.010448721	62
CCAGGACGAACAGACAAUGAA	0	0.007117064	49
CCAGGCAGGACAGACAACGAA	0	0.006699818	18
CCAGGGCGAACAGACAAUGAA	0	0.005029084	41
CCGGGGCGAACAGACAAUGAA	0	0.009616856	63
CCGGGUCGAACUGACAACGCG	0	0.016252243	15
CCAGGUAGAACAGAUAACGGA	1	0.021294053	4
CCAGGCCGAACAGACAAUGAA	0	0.004192663	35
CCAGGAAGGACAGACAAUGAA	0	0.021199593	2
CCAGGGCGAACAGACAAUGAA	0	0.005447031	44
UACCUGAUCAAUUGGCUGGUG	2	0.028858663	3
CAGAAAAAGAGAUGUACAGAUGG	0	0.045164539	63
AGGAGGAGGAGUGGGGGGACGG	0	0.000839942	16

Gene annotation
Polyphenol oxidase, chloroplastic OS=Malus domestica PE=2 SV=1
Polyphenol oxidase, chloroplastic OS=Malus domestica PE=2 SV=1
Polyphenol oxidase, chloroplastic OS=Malus domestica PE=2 SV=1
Polyphenol oxidase, chloroplastic OS=Malus domestica PE=2 SV=1
Polyphenol oxidase, chloroplastic OS=Malus domestica PE=2 SV=1
Polyphenol oxidase, chloroplastic OS=Malus domestica PE=2 SV=1
Probable carbohydrate esterase At4g34215 OS=Arabidopsis thaliana GN=At4g34215 PE=1 SV=2
Protein GrpE OS=Xylella fastidiosa (strain Temecula1 / ATCC 700964) GN=grpE PE=3 SV=2
Teosinte glume architecture 1 OS=Zea mays GN=TGA1 PE=3 SV=1
Squamosa promoter-binding-like protein 6 OS=Arabidopsis thaliana GN=SPL6 PE=1 SV=2
Squamosa promoter-binding-like protein 17 OS=Oryza sativa subsp. japonica GN=SPL17 PE=2 SV=2
Squamosa promoter-binding-like protein 13B OS=Arabidopsis thaliana GN=SPL13B PE=3 SV=1
Squamosa promoter-binding-like protein 13B OS=Arabidopsis thaliana GN=SPL13B PE=3 SV=1
Squamosa promoter-binding-like protein 14 OS=Oryza sativa subsp. japonica GN=SPL14 PE=2 SV=1
Squamosa promoter-binding-like protein 12 OS=Oryza sativa subsp. indica GN=SPL12 PE=2 SV=1
Squamosa promoter-binding-like protein 12 OS=Oryza sativa subsp. indica GN=SPL12 PE=2 SV=1
Squamosa promoter-binding-like protein 13B OS=Arabidopsis thaliana GN=SPL13B PE=3 SV=1
Squamosa promoter-binding-like protein 16 OS=Oryza sativa subsp. japonica GN=SPL16 PE=2 SV=1
Probable polyribonucleotide nucleotidyltransferase 1, chloroplastic OS=Oryza sativa subsp. japonica GN=PNP1 PE=
Squamosa promoter-binding-like protein 12 OS=Oryza sativa subsp. indica GN=SPL12 PE=2 SV=1
Squamosa promoter-binding-like protein 6 OS=Arabidopsis thaliana GN=SPL6 PE=1 SV=2
Squamosa promoter-binding-like protein 6 OS=Arabidopsis thaliana GN=SPL6 PE=1 SV=2
Squamosa promoter-binding-like protein 6 OS=Arabidopsis thaliana GN=SPL6 PE=1 SV=2
Squamosa promoter-binding-like protein 13B OS=Arabidopsis thaliana GN=SPL13B PE=3 SV=1
Squamosa promoter-binding-like protein 12 OS=Oryza sativa subsp. indica GN=SPL12 PE=2 SV=1
Protein SPEAR1 OS=Arabidopsis thaliana GN=SPEAR1 PE=1 SV=1
Protein SPEAR1 OS=Arabidopsis thaliana GN=SPEAR1 PE=1 SV=1
Transcription factor GAMYB OS=Oryza sativa subsp. japonica GN=GAMYB PE=1 SV=1
Transcription factor GAMYB OS=Oryza sativa subsp. japonica GN=GAMYB PE=1 SV=1
Receptor-like protein kinase HSL1 OS=Arabidopsis thaliana GN=HSL1 PE=2 SV=1
Auxin response factor 18 OS=Oryza sativa subsp. japonica GN=ARF18 PE=2 SV=1
Auxin response factor 18 OS=Oryza sativa subsp. japonica GN=ARF18 PE=2 SV=1
Auxin response factor 17 OS=Arabidopsis thaliana GN=ARF17 PE=2 SV=1
Solute carrier family 25 member 44 OS=Pongo abelii GN=SLC25A44 PE=2 SV=2
Auxin response factor 18 OS=Oryza sativa subsp. japonica GN=ARF18 PE=2 SV=1
Auxin response factor 18 OS=Oryza sativa subsp. japonica GN=ARF18 PE=2 SV=1
Auxin response factor 17 OS=Arabidopsis thaliana GN=ARF17 PE=2 SV=1
Auxin response factor 18 OS=Oryza sativa subsp. japonica GN=ARF18 PE=2 SV=1
Auxin response factor 18 OS=Oryza sativa subsp. japonica GN=ARF18 PE=2 SV=1
Endoribonuclease Dicer homolog 1 OS=Arabidopsis thaliana GN=DCL1 PE=1 SV=2
NAC domain-containing protein 100 OS=Arabidopsis thaliana GN=NAC100 PE=2 SV=1
NAC domain-containing protein 100 OS=Arabidopsis thaliana GN=NAC100 PE=2 SV=1
NAC domain-containing protein 21/22 OS=Arabidopsis thaliana GN=NAC021 PE=1 SV=2
U-box domain-containing protein 35 OS=Arabidopsis thaliana GN=PUB35 PE=2 SV=2
NAC domain-containing protein 21/22 OS=Arabidopsis thaliana GN=NAC021 PE=1 SV=2

Homeobox-leucine zipper protein ATHB-15 OS=Arabidopsis thaliana GN=ATHB-15 PE=1 SV=1
Homeobox-leucine zipper protein ATHB-8 OS=Arabidopsis thaliana GN=ATHB-8 PE=1 SV=1
Homeobox-leucine zipper protein ATHB-8 OS=Arabidopsis thaliana GN=ATHB-8 PE=1 SV=1
Glucan endo-1,3-beta-glucosidase 14 OS=Arabidopsis thaliana GN=At2g27500 PE=1 SV=2
Homeobox-leucine zipper protein REVOLUTA OS=Arabidopsis thaliana GN=REV PE=1 SV=2
Homeobox-leucine zipper protein HOX32 OS=Oryza sativa subsp. japonica GN=HOX32 PE=2 SV=1
Homeobox-leucine zipper protein HOX32 OS=Oryza sativa subsp. japonica GN=HOX32 PE=2 SV=1
Homeobox-leucine zipper protein ATHB-15 OS=Arabidopsis thaliana GN=ATHB-15 PE=1 SV=1
Homeobox-leucine zipper protein REVOLUTA OS=Arabidopsis thaliana GN=REV PE=1 SV=2
Auxin response factor 8 OS=Arabidopsis thaliana GN=ARF8 PE=1 SV=2
Auxin response factor 8 OS=Arabidopsis thaliana GN=ARF8 PE=1 SV=2
Auxin response factor 6 OS=Arabidopsis thaliana GN=ARF6 PE=1 SV=2
Auxin response factor 6 OS=Arabidopsis thaliana GN=ARF6 PE=1 SV=2
Auxin response factor 6 OS=Arabidopsis thaliana GN=ARF6 PE=1 SV=2
Auxin response factor 6 OS=Arabidopsis thaliana GN=ARF6 PE=1 SV=2
Protein argonaute 1 OS=Arabidopsis thaliana GN=AGO1 PE=1 SV=1
Thaumatin-like protein 1 OS=Arabidopsis thaliana GN=TLP1 PE=2 SV=1
Pentatricopeptide repeat-containing protein At2g17670 OS=Arabidopsis thaliana GN=At2g17670 PE=2 SV=1
Nodulation-signaling pathway 2 protein OS=Medicago truncatula GN=NSP2 PE=1 SV=1
Nodulation-signaling pathway 2 protein OS=Medicago truncatula GN=NSP2 PE=1 SV=1
Phosphopentomutase OS=Shewanella baltica (strain OS195) GN=deoB PE=3 SV=1
Scarecrow-like protein 6 OS=Arabidopsis thaliana GN=SCL6 PE=1 SV=1
Scarecrow-like protein 6 OS=Arabidopsis thaliana GN=SCL6 PE=1 SV=1
Alkylated DNA repair protein alkB homolog 8 OS=Xenopus tropicalis GN=alkbh8 PE=2 SV=2
Scarecrow-like protein 6 OS=Arabidopsis thaliana GN=SCL6 PE=1 SV=1
Floral homeotic protein APETALA 2 OS=Arabidopsis thaliana GN=AP2 PE=1 SV=1
Floral homeotic protein APETALA 2 OS=Arabidopsis thaliana GN=AP2 PE=1 SV=1
Ethylene-responsive transcription factor RAP2-7 OS=Arabidopsis thaliana GN=RAP2-7 PE=2 SV=2
Floral homeotic protein APETALA 2 OS=Arabidopsis thaliana GN=AP2 PE=1 SV=1
Floral homeotic protein APETALA 2 OS=Arabidopsis thaliana GN=AP2 PE=1 SV=1
Ethylene-responsive transcription factor RAP2-7 OS=Arabidopsis thaliana GN=RAP2-7 PE=2 SV=2
Ethylene-responsive transcription factor RAP2-7 OS=Arabidopsis thaliana GN=RAP2-7 PE=2 SV=2
Ethylene-responsive transcription factor RAP2-7 OS=Arabidopsis thaliana GN=RAP2-7 PE=2 SV=2
F-box/kelch-repeat protein At3g27150 OS=Arabidopsis thaliana GN=At3g27150 PE=2 SV=1
Protein JINGUBANG OS=Arabidopsis thaliana GN=JGB PE=1 SV=1
Protein MARD1 OS=Arabidopsis thaliana GN=MARD1 PE=2 SV=2
Transcription factor GAMYB OS=Oryza sativa subsp. japonica GN=GAMYB PE=1 SV=1
Transcription factor MYB101 OS=Arabidopsis thaliana GN=MYB101 PE=2 SV=1
COBRA-like protein 4 OS=Arabidopsis thaliana GN=COBL4 PE=2 SV=2
ATP sulfurylase 1, chloroplastic OS=Arabidopsis thaliana GN=APS1 PE=1 SV=1
Low affinity sulfate transporter 3 OS=Stylosanthes hamata GN=ST3 PE=2 SV=1
ATP sulfurylase 1, chloroplastic OS=Arabidopsis thaliana GN=APS1 PE=1 SV=1
Sulfate transporter 2.1 OS=Arabidopsis thaliana GN=SULTR2;1 PE=2 SV=1
Probable pectate lyase 22 OS=Arabidopsis thaliana GN=At5g63180 PE=2 SV=1
Growth-regulating factor 5 OS=Arabidopsis thaliana GN=GRF5 PE=1 SV=1
Caffeoylshikimate esterase OS=Arabidopsis thaliana GN=CSE PE=1 SV=1
Growth-regulating factor 6 OS=Oryza sativa subsp. japonica GN=GRF6 PE=2 SV=2

Growth-regulating factor 1 OS=Arabidopsis thaliana GN=GRF1 PE=1 SV=1
Growth-regulating factor 5 OS=Arabidopsis thaliana GN=GRF5 PE=1 SV=1
Dynein light chain, cytoplasmic OS=Dictyostelium discoideum GN=dlcB PE=3 SV=1
Growth-regulating factor 8 OS=Arabidopsis thaliana GN=GRF8 PE=2 SV=1
Growth-regulating factor 5 OS=Oryza sativa subsp. japonica GN=GRF5 PE=2 SV=1
Growth-regulating factor 7 OS=Arabidopsis thaliana GN=GRF7 PE=2 SV=1
Growth-regulating factor 12 OS=Oryza sativa subsp. japonica GN=GRF12 PE=2 SV=1
Growth-regulating factor 4 OS=Arabidopsis thaliana GN=GRF4 PE=1 SV=1
60S ribosomal protein L18-2 OS=Arabidopsis thaliana GN=RPL18B PE=1 SV=2
Growth-regulating factor 8 OS=Arabidopsis thaliana GN=GRF8 PE=2 SV=1
Growth-regulating factor 4 OS=Arabidopsis thaliana GN=GRF4 PE=1 SV=1
E3 ubiquitin-protein ligase CHIP OS=Arabidopsis thaliana GN=CHIP PE=1 SV=1
Transcription factor LHW OS=Arabidopsis thaliana GN=LHW PE=1 SV=1
Growth-regulating factor 4 OS=Oryza sativa subsp. japonica GN=GRF4 PE=2 SV=1
Growth-regulating factor 6 OS=Oryza sativa subsp. japonica GN=GRF6 PE=2 SV=2
Growth-regulating factor 5 OS=Arabidopsis thaliana GN=GRF5 PE=1 SV=1
Growth-regulating factor 1 OS=Arabidopsis thaliana GN=GRF1 PE=1 SV=1
Laccase-5 OS=Arabidopsis thaliana GN=LAC5 PE=2 SV=1
Laccase-7 OS=Arabidopsis thaliana GN=LAC7 PE=2 SV=1
Laccase-7 OS=Arabidopsis thaliana GN=LAC7 PE=2 SV=1
Laccase-3 OS=Arabidopsis thaliana GN=LAC3 PE=2 SV=2
Multicopper oxidase LPR2 OS=Arabidopsis thaliana GN=LPR2 PE=2 SV=1
Copper transporter 6 OS=Arabidopsis thaliana GN=COPT6 PE=2 SV=1
Copper transporter 6 OS=Arabidopsis thaliana GN=COPT6 PE=2 SV=1
Umecyanin OS=Armoracia rusticana PE=1 SV=1
Copper chaperone for superoxide dismutase, chloroplastic/cytosolic OS=Arabidopsis thaliana GN=CCS PE=1 SV=1
Copper chaperone for superoxide dismutase, chloroplastic/cytosolic OS=Arabidopsis thaliana GN=CCS PE=1 SV=1
Dehydration-responsive element-binding protein 2A OS=Arabidopsis thaliana GN=DREB2A PE=1 SV=1
Superoxide dismutase [Cu-Zn], chloroplastic OS=Solidago canadensis var. scabra GN=SODCP PE=2 SV=1
LIM domain-containing protein WLIM1 OS=Arabidopsis thaliana GN=WLIM1 PE=1 SV=1
Heme-binding protein 2 OS=Homo sapiens GN=HEBP2 PE=1 SV=1
Basic blue protein OS=Arabidopsis thaliana GN=ARPN PE=2 SV=2
Nudix hydrolase 23, chloroplastic OS=Arabidopsis thaliana GN=NUDT23 PE=1 SV=2
DTW domain-containing protein 2 OS=Macaca fascicularis GN=DTWD2 PE=2 SV=1
DELLA protein GAI1 OS=Vitis vinifera GN=GAI1 PE=2 SV=1
Chaperone protein dnaJ 8, chloroplastic OS=Arabidopsis thaliana GN=ATJ8 PE=2 SV=1
Chaperone protein dnaJ 8, chloroplastic OS=Arabidopsis thaliana GN=ATJ8 PE=2 SV=1
Disease resistance protein At4g27190 OS=Arabidopsis thaliana GN=At4g27190 PE=2 SV=1
Putative disease resistance protein RGA1 OS=Solanum bulbocastanum GN=RGA1 PE=2 SV=2
Putative disease resistance protein RGA3 OS=Solanum bulbocastanum GN=RGA3 PE=2 SV=2
Probable apyrase 6 OS=Arabidopsis thaliana GN=APY6 PE=2 SV=2
Putative disease resistance protein At1g50180 OS=Arabidopsis thaliana GN=At1g50180 PE=3 SV=2
Dihydropyrimidine dehydrogenase (NADP(+)), chloroplastic OS=Arabidopsis thaliana GN=PYD1 PE=1 SV=1
Ribosome maturation factor RimM OS=Magnetospirillum magneticum (strain AMB-1 / ATCC 700264) GN=rimM PE
Pyrophosphate-energized vacuolar membrane proton pump OS=Vigna radiata var. radiata PE=1 SV=4
Putative pentatricopeptide repeat-containing protein At1g12700, mitochondrial OS=Arabidopsis thaliana GN=At1[illegible]
Zinc transporter 1 OS=Arabidopsis thaliana GN=ZIP1 PE=2 SV=1

Thioredoxin-related transmembrane protein 2 OS=Xenopus tropicalis GN=tmx2 PE=2 SV=1
Transcription repressor MYB4 OS=Arabidopsis thaliana GN=MYB4 PE=1 SV=1
Transcription factor MYB26 OS=Arabidopsis thaliana GN=MYB26 PE=2 SV=1
Anthocyanin regulatory C1 protein OS=Zea mays GN=C1 PE=2 SV=1
Transcription factor MYB3 OS=Arabidopsis thaliana GN=MYB3 PE=1 SV=1
Transcription factor MYB15 OS=Arabidopsis thaliana GN=MYB15 PE=1 SV=1
Anthocyanin regulatory C1 protein OS=Zea mays GN=C1 PE=2 SV=1
Anthocyanin regulatory C1 protein OS=Zea mays GN=C1 PE=2 SV=1
Transcription factor MYB44 OS=Arabidopsis thaliana GN=MYB44 PE=1 SV=1
Transcription factor MYB1 OS=Arabidopsis thaliana GN=MYB1 PE=2 SV=1
Transcription repressor MYB4 OS=Arabidopsis thaliana GN=MYB4 PE=1 SV=1
Transcription factor MYB102 OS=Arabidopsis thaliana GN=MYB102 PE=2 SV=1
Transcription factor TT2 OS=Arabidopsis thaliana GN=TT2 PE=1 SV=1
Molybdenum cofactor sulfurase OS=Solanum lycopersicum GN=FLACCA PE=2 SV=1
Probable galactinol--sucrose galactosyltransferase 2 OS=Arabidopsis thaliana GN=RFS2 PE=2 SV=2
Probable glucose uptake protein GlcU OS=Staphylococcus epidermidis (strain ATCC 12228) GN=glcU PE=3 SV=1

P-value
0
0
1.38E-96
0
0
0
7.3E-86
5.3
5.49E-47
4.01E-42
1.9E-52
5.71E-69
6.8E-40
9.69E-65
1.29E-75
1.21E-73
5.71E-69
1.09E-40
1.8E-09
1.29E-75
8.78E-34
6.09E-44
1.09E-35
5.58E-61
1.21E-73
1.2
0.38
7.18E-22
2.12E-125
0
0
0
7.43E-164
5.99E-30
0
0
3.78E-160
0
0
0
2.86E-146
1.09E-143
4.62E-113
0
5.67E-113

0
0
0
0
0
0
0
0
0
0
0
0
0
0
0
0
1.2E-94
0
0
0
0.59
6.77E-123
2.49E-118
6.65E-42
5.03E-122
3.72E-80
3.34E-105
4.11E-96
3.7E-90
1.83E-111
2.25E-104
3.32E-67
2.17E-64
2.07E-06
8.75E-57
2.39E-07
5.66E-124
5.15E-64
0.93
0
0
0
0
0
1.72E-58
6.77E-46
3.68E-80

3.33E-104
7.99E-51
3.13E-20
2.02E-42
3.35E-46
2.16E-32
2.77E-36
7.73E-74
2.83E-121
4.67E-38
4.04E-81
8.08E-136
1.51E-67
6.24E-56
8.32E-77
9.85E-45
3.99E-102
0
0
0
0
0
1.39E-16
1.79E-17
1.44E-24
9.47E-128
1.52E-116
2.61E-28
1.27E-102
1.28E-90
2.34E-21
1.26E-37
3.4E-116
2.66E-20
3.24E-78
2.38E-35
3.37E-40
1.31E-85
9.94E-78
4.06E-81
0
1.36E-62
0
0.37
0
2.19E-10
9.95E-121

6.67E-18
1.13E-40
1.25E-61
1.17E-61
2.16E-60
7.07E-40
6.44E-61
5.37E-62
2.89E-54
1.53E-80
1.38E-60
9.88E-98
3.07E-62
5.97E-12
1.12E-180
0.3
